# Isoquercitrin ameliorates chondrocyte dysfunction in osteoarthritis by targeting AKT1-mediated mitochondrial dynamics regulation

**DOI:** 10.1371/journal.pone.0357989

**Published:** 2026-09-17

**Authors:** Yuan Ding, Xiang Wang, Shihua Zong, Qiong Wen, Luyang Zhang

**Affiliations:** 1 Department of Rheumatology and Immunology, Yantaishan Hospital, Yantai, Shandong Province, China; 2 Department of Nephrology, Yantaishan Hospital, Yantai, Shandong Province, China; University College London, UNITED KINGDOM OF GREAT BRITAIN AND NORTHERN IRELAND

## Abstract

Osteoarthritis (OA), a prevalent degenerative joint disorder, is driven by chondrocyte anabolic-catabolic imbalance, aberrant inflammation, and mitochondrial dysfunction. Disease-modifying therapies targeting these pathological processes remain scarce, limiting effective clinical intervention. Herein, we demonstrate an isoquercitrin (IQ)-based chondroprotective strategy that rectifies osteoarthritis-related chondrocyte impairment by targeting *AKT1*. IQ first reverses IL-1β-induced chondrocyte proliferation arrest, shifts metabolic homeostasis toward anabolism, and mitigates pro-inflammatory responses. It simultaneously restores mitochondrial homeostasis by normalizing dynamic balance. Mechanistically, IQ binds to AKT1 with high affinity, and *AKT1* overexpression abolishes its protective effects—confirming *AKT1* as a key mediator. This approach re-establishes chondrocyte functional homeostasis by coupling anti-inflammatory, metabolic-regulatory, and mitochondrial-stabilizing activities. This work highlights IQ as a promising candidate for osteoarthritis intervention and provides a mechanistic framework linking *AKT1* targeting to chondroprotection.

## Introduction

Osteoarthritis (OA) is one of the most widespread whole-joint degenerative diseases globally, imposing a substantial socioeconomic burden due to chronic joint pain and locomotor disability in aging populations [1–3]. Contemporary pathological consensus has advanced beyond the outdated “single-cartilage wear” view, defining OA as a heterogeneous whole-joint disorder characterized by abnormal subchondral bone remodelling, progressive meniscal matrix deterioration, persistent synovial inflammation, and inflammatory infiltration of the infrapatellar fat pad [4–7]. Major predisposing risk factors include advanced age, obesity, mechanical overloading, articular trauma, and systemic metabolic dysfunction [8]. Importantly, the traditional wear-driven theory fails to account for progressive cartilage loss in a significant subset of OA patients without excessive joint loading, suggesting that non-mechanical inflammatory and intracellular pathological cascades play a dominant role in disease progression [9,10].

The core pathological hallmark of OA lies in chondrocyte anabolic-catabolic dysregulation: marked by diminished extracellular matrix (ECM) production and heightened secretion of matrix-degrading enzymes [11–13]. Crucially, this dysregulation is exacerbated by the inflamed infrapatellar fat pad (IFP) and synovial membrane, which secrete a plethora of pro-inflammatory cytokines, such as interleukin-1β (IL-1β) and tumor necrosis factor-α (TNF-α), into the joint cavity [14,15]. Parallel to this, pro-inflammatory cytokines perpetuate this imbalance by stimulating catabolic signaling cascades, while disrupted mitochondrial dynamics further worsen chondrocyte dysfunction via excessive reactive oxygen species (ROS) generation [16,17]. Mitochondrial dysfunction therefore acts as a critical downstream effector linking multi-tissue lesions of whole joint to irreversible chondrocyte injury. Despite these insights, disease-modifying OA therapies remain scarce. Thus, identifying agents that simultaneously reestablish chondrocyte metabolic stability, curb inflammation, and reverse mitochondrial dysfunction addresses a pressing unmet clinical need.

Given this therapeutic gap, nutraceutical-derived small natural molecules have emerged as promising disease-modifying OA drug (DMOAD) candidates due to their superior biosafety and multitarget anti-inflammatory properties [18,19]. For instance, the combinatorial low-dose administration of β-caryophyllene, ascorbic acid, and D-glucosamine has been shown to alleviate inflammatory chondrocyte injury [20]. Isoquercitrin (IQ), a naturally occurring flavonol glycoside, exhibits potent antioxidant and anti-inflammatory capacities and protects against various pathological states (e.g., ischemia-reperfusion injury, neurodegeneration) [21–25]. IQ also known as quercetin-3-O-glucoside, is an extensively distributed natural flavonol glycoside that accumulates in the roots, stems, leaves, and fruits of diverse plant species—including *Mangifera indica*, *Rheum nobile*, *Annona squamosa*, and *Camellia sinensis* [26,27]. IQ has better bioavailability than its parent molecule, yet its function in OA has been scarcely investigated [28–30]. Early in vitro studies hint that IQ may regulate cytokine-driven inflammation, but its chondroprotective potential in OA, along with the associated molecular targets and mechanisms, remain uncharacterized. Notably, RAC-alpha serine/threonine-protein Kinase (*AKT1*) is a master upstream modulator governing chondrocyte mitochondrial dynamics and metabolic homeostasis [31–33]. Persistent IL-1β stimulation triggers pathological sustained hyperactivation of AKT1, inducing excessive mitochondrial fission [32]. Whether IQ directly targets AKT1 as an allosteric buffer to normalize its overactive signaling and alleviate IL-1β-mediated mitochondrial and cartilage catabolic defects has not been experimentally verified.

To fill the above-mentioned research gap that the direct interaction between IQ and *AKT1* as well as the downstream chondroprotective cascade remains unclarified in OA pathogenesis, the present study was designed to explore the chondroprotective efficacy and underlying molecular mechanism of IQ against OA progression. Specifically, we will employ an IL-1β-stimulated OA-like chondrocyte model to evaluate the impact of IQ on metabolic homeostasis, inflammatory responses, and mitochondrial integrity. Furthermore, we will utilize molecular docking and dynamic simulation analyses to identify high-affinity targets of IQ, followed by gain-of-function experiments to validate *AKT1* as a key mediator. Ultimately, we seek to elucidate the mechanistic link between *AKT1* inhibition, restoration of mitochondrial dynamics, and rebalancing of chondrocyte metabolism. Our findings are expected to establish IQ as a DMOAD candidate and provide a novel mechanistic framework for OA therapeutic intervention.

## Materials and methods

### Materials

C28/I2 human chondrocyte was purchased from Shanghai Yaji Biotechnology (Shanghai, China). Isoquercitrin (IQ, Cat.SM2187, ≥ 98% purity) was obtained from Beyotime Biotechnology (Shanghai, China). Recombinant human IL-1β was sourced from R&D Systems (MN, USA). DMEM medium, FBS and penicillin-streptomycin were supplied by Gibco (USA); trypsin, Polybrene and Triton X-100 were from Sigma-Aldrich (USA). CCK-8, EdU, JC-1 (Cat.D-9113), RIPA lysis buffer and DAPI staining kits were purchased from Dojindo, Beyotime and Bioss Biotechnology (Beijing, China), respectively. SDS-PAGE kit, BCA quantification kit and PVDF membrane were from Epizyme and Millipore (USA). All primary antibodies were acquired from Proteintech, MedChemExpress and Abcam; HRP-conjugated secondary antibodies were obtained from Jackson ImmunoResearch (USA). RNA extraction, reverse transcription and qPCR related kits were products of Takara (Japan) and GenStar (China). Lentiviruses for *AKT1* overexpression and empty vector were constructed by Shanghai Jima Pharmaceutical Technology (Shanghai, China). GraphPad Prism, R v4.3.3, Cytoscape 3.8.2 and Gromacs 2022 software were used for statistical calculation, network construction and molecular dynamics simulation.

### Cell culture and treatment

C28/I2 human chondrocytes were procured from Shanghai Yaji Biotechnology Co., LTD and cultured in high-glucose dulbecco’s modified eagle’s medium (DMEM) with 10% fetal bovine serum (FBS) and 1% penicillin-streptomycin at 37 °C in a 5% CO_2_ atmosphere. Cells were divided into control, IL-1β-treated, and IQ-treated groups at 70% confluence. The IL-1β group was incubated with 10 ng/mL IL-1β for 24 h, whereas the IQ group was co-incubated with 10 ng/mL IL-1β and 40 μM IQ for 24 h.

### Cell counting kit‑8 (CCK-8) assay for IQ treatment concentration

C28/I2 cells were seeded at a density of 3 × 10^3^ cells per well in 96-well plates containing 100 μL medium and incubated for 24 h. Cells were then exposed to IQ (Beyotime, SM2187) at concentrations of 0, 5, 10, 20, 40, 80 and 160 μM for another 24 h. Subsequently, 10 μL of CCK-8 reagent was supplemented to each well; blank control wells contained only CCK-8 reagent without cells. After 2–4 h of incubation in the dark, optical density (OD) values were measured at 490 nm via a Multiskan FC microplate reader (Thermo Fisher Scientific, USA).

Two parallel CCK‑8 assays were performed separately: First, cytotoxic screening of IQ without IL‑1β stimulation: cells were exposed to IQ at concentrations of 0, 20, 40, 60, 80, 100, 120, 140, 160 μM for another 24 h to calculate the IC₅₀ value of IQ under normal culture conditions. Second, dose–response detection under IL‑1β-induced inflammatory injury: chondrocytes were co-incubated with 10 ng/mL IL‑1β and serial IQ concentrations (0, 5, 10, 20, 40, 80 μM) for 24 h. Subsequently, 10 μL of CCK-8 reagent was supplemented to each well. After 2–4 h of incubation in the dark, OD values were measured at 490 nm via a Multiskan FC microplate reader (Thermo Fisher Scientific, USA). Cell viability was calculated using the formula:


$$\begin{array}{c} \text{Viability(\textbackslash{}\%)}=\left[\frac{OD_{\text{Treated}}-OD_{\text{Blank}}}{OD_{\text{Control}}-OD_{\text{Black}}}\right] \end{array}$$ (1)

### EdU staining for cell proliferation

C28/I2 cells were seeded in 96-well plates at 3 × 10^3^ cells/well and treated with IL-1β and/or IQ at 70% confluence for 24 h per group allocation. After incubating with EdU working solution for 2–4 h, cells were fixed with 4% paraformaldehyde (PFA, 15 min), washed three times with phosphate-buffered saline (PBS), and permeabilized with 0.3% Triton X-100 (15 min, room temperature). Following an additional PBS wash, the click reaction was performed using the kit-provided reaction mixture according to the manufacturer’s instructions for 30 min at room temperature in the dark. Nuclei were counterstained with DAPI for 10 min in the dark. Fluorescence images were captured using an inverted fluorescence microscope (DMIL LED, Leica Microsystems, Wetzlar, Germany) equipped with a 10 × objective lens. Cell proliferation was quantified by counting the number of EdU‑positive cells per field with ImageJ (National Institutes of Health, Bethesda, MD, USA; https://imagej.net/ij/) [34]; 3 random non‑overlapping fields were counted per sample in a blinded manner.

### Western blotting

Cells from each treatment group were collected, and total protein was extracted. Total protein was isolated using radio-immunoprecipitation assay (RIPA) lysis buffer containing protease and phosphatase inhibitor cocktails. C28/I2 chondrocytes were collected, rinsed with cold PBS, lysed on ice, and centrifuged to harvest supernatants. Protein concentration was quantified via the bicinchoninic acid (BCA) assay. Equal amounts of 30 µg protein per sample were separated on two distinct SDS-PAGE gels according to target molecular weights: 6% constant gels for high-molecular-weight proteoglycans (e.g., aggrecan (ACAN)), and 12% constant gels for low-molecular-weight proteins. After transfer to polyvinylidene fluoride (PVDF) membranes, membranes were blocked with 5% (w/v) bovine serum albumin (BSA) dissolved in TBST for 1 hour at room temperature, rinsed with TBST, and incubated overnight at 4 °C with primary antibodies (β-actin: 66009–1-Ig, 1:10000, Proteintech; matrix metallopeptidase 1 (MMP-1): HY-P80754, 1:1000, MedChemExpress; matrix metallopeptidase 13 (MMP-13): YA3143, 1:1000, MedChemExpress; SRY-box transcription factor 9 (SOX9): HY-P80355, 1:500, MedChemExpress; type II collagen (COL2): HY-P81047, 1:2000, MedChemExpress; ACAN: ab3778, 1:1000, Abcam; interleukin 6 (IL-6): ab233706, 1:1000, Abcam; interleukin 1 beta (IL-1β): HY-P80503, 1:1000, MedChemExpress; tumor necrosis factor alpha (TNF-α): HY-P80914, 1:800, MedChemExpress; optic atrophy 1 (OPA1): ab42364, 1:1000, Abcam; mitofusin 1 (MFN1): ab221661, 1:1000, Abcam; mitofusin 2 (MFN2): ab56889, 1:1000, Abcam; mitochondrial fission 1 protein (FIS1): ab156865, 1:10000, Abcam; dynamin-related protein 1 (DRP1): 12957–1-AP, 1:5000, Proteintech; DRP1 (phospho S616): ab314755, 1:1000, Abcam). Then, membranes were incubated with horseradish peroxidase (HRP)-conjugated anti-rabbit or anti-mouse secondary antibodies (1:5000) for 1 h at room temperature. Finally, the membranes were observed by an enhanced chemiluminescence (ECL) kit (Millipore, Billerica, MA, USA). Unmodified original blot images were quantified with ImageJ software (National Institutes of Health, Bethesda, MD, USA; https://imagej.net/ij/) [34]: images were converted to grayscale, background was subtracted uniformly, and relative protein expression was calculated as the integrated density of target band divided by that of β‑actin (internal control).

### Quantitative real-time polymerase chain reaction (qRT-PCR)

Total RNA was isolated from treated cells using the Universal RNA Extraction Kit (Takara, Cat. No. 9767). RNA concentration and purity were measured with a Multiscan Sky spectrophotometer (ThermoFisher Scientific Inc., USA). Complementary DNA (cDNA) was generated via the PrimeScript 1st Strand cDNA Synthesis Kit (Takara, Cat. No. 6110A), and qRT-PCR was conducted with SYBR Premix (GenStar, Cat. No. A304-10) on the QuantStudio 3 real-time PCR system (ThermoFisher Scientific Inc., USA). The amplification protocol consisted of initial denaturation at 95 °C for 30 s, followed by 40 cycles of 95 °C for 5 s and 60 °C for 35 s. The relative gene expression was calculated by the 2^(-ΔΔCt) method and normalized to *GAPDH*. The primer sequences used for qRT-PCR are provided in Table 1.

**Table 1:** All primer sequences were used for qRT-PCR in this work.

Name	Forward	Reverse
*MMP1*	5’-AAAATTACACGCCAGATTTGCC-3’	5’-GGTGTGACATTACTCCAGAGTTG-3’
*MMP13*	5’-ACTGAGAGGCTCCGAGAAATG-3’	5’-GAACCCCGCATCTTGGCTT-3’
*SOX9*	5’-AGCGAACGCACATCAAGAC-3’	5’-CTGTAGGCGATCTGTTGGGG-3’
*COL2*	5’-TGGACGATCAGGCGAAACC-3’	5’-GCTGCGGATGCTCTCAATCT-3’
*ACAN*	5’-GTGCCTATCAGGACAAGGTCT-3’	5’-GATGCCTTTCACCACGACTTC-3’
*IL6*	5’-ACTCACCTCTTCAGAACGAATTG-3’	5’-CCATCTTTGGAAGGTTCAGGTTG-3’
*IL1B*	5’-ATGATGGCTTATTACAGTGGCAA-3’	5’-GTCGGAGATTCGTAGCTGGA-3’
*TNF*	5’-CCTCTCTCTAATCAGCCCTCTG-3’	5’-GAGGACCTGGGAGTAGATGAG-3’
*OPA1*	5’-TGTGAGGTCTGCCAGTCTTTA-3’	5’-TGTCCTTAATTGGGGTCGTTG-3’
*MFN1*	5’-TGGCTAAGAAGGCGATTACTGC-3’	5’-GCAGGCTGGTGGTATTCTTG-3’
*MFN2*	5’-CTCTCGATGCAACTCTATCGTC-3’	5’-TCCTGTACGTGTCTTCAAGGAA-3
*FIS1*	5’-GTCCAAGAGCACGCAGTTTG-3’	5’-GCTGGTGATGGTCTTGGTG-3’
*GAPDH*	5’-GGAGCGAGATCCCTCCAAAAT-3’	5’-GGCTGTTGTCATACTTCTCATGG-3’

### JC-1 staining

C28/I2 cells were seeded in 24-well plates at 1 × 10^5^ cells per well and treated with IL-1β and/or IQ at 70% confluence for 24 h. JC-1 staining solution was prepared per the kit instructions (D-9113, Bioss). The working solution was prepared by diluting the JC-1 stock (200×) with ultrapure water and JC-1 staining buffer (5×) according to the standard protocol. Post-treatment, cells were rinsed with pre-warmed PBS, incubated with JC-1 working solution at 37 °C for 20 min in the dark, then washed twice with ice-cold JC-1 staining buffer (1×). Fluorescence signals were visualized using an inverted fluorescence microscope (Leica DMIL LED, Leica Microsystems, Wetzlar, Germany), with red fluorescence (JC-1 aggregates) and green fluorescence (JC-1 monomers) detected at excitation/emission wavelengths of 525/590 nm and 490/530 nm, respectively.

### Immunofluorescence staining for TOM20 and 8-OHdG

C28/I2 were seeded on glass coverslips and treated with vehicle (Con), IL-1β (10 ng/mL), IL-1β + IQ (40 μM), or IL-1β + IQ + *AKT1* overexpression. After treatment, cells were fixed with 4% paraformaldehyde for 15 min, permeabilized with 0.5% Triton X-100 in PBS for 10 min, and blocked with 5% bovine serum albumin (BSA) for 1 h at room temperature. Cells were then incubated overnight at 4°C with primary antibodies: rabbit anti-TOM20 (1:200, Abcam) and mouse anti-8-OHdG (1:500, Abcam). After washing, cells were incubated with Alexa Fluor 555-conjugated anti-rabbit IgG (1:500, Thermo Fisher) and Alexa Fluor 488-conjugated anti-mouse IgG (1:500, Thermo Fisher) for 1 h at room temperature. Nuclei were counterstained with DAPI (1 μg/mL) for 5 min. Images were acquired using an inverted fluorescence microscope (Leica DMIL LED, Leica Microsystems, Wetzlar, Germany). The mean fluorescence intensity (MFI) of 8-OHdG was quantified using ImageJ software (National Institutes of Health, Bethesda, MD, USA; https://imagej.net/ij/) [34].

### Target collection of IQ

The chemical structure of IQ was retrieved from the PubChem database (CID: 5280804). Five online databases were used to predict potential human targets of isoquercitrin with specific screening thresholds: bioinformatics analysis tool for molecular mechanism of traditional chinese medicine (BATMAN-TCM) (http://bionet.ncpsb.org.cn/batman-tcm/) (score cutoff > 0.84), SwissTargetPrediction (http://www.swisstargetprediction.ch/) (probability > 0), a high‑throughput experiment- and reference-guided database of traditional Chinese medicine (HERB) (http://herb.ac.cn/), PharmMapper (PM, https://lilab-ecust.cn/pharmmapper/submitfile.html), and TargetNet (http://targetnet.scbdd.com/calcnet/index/).

In total, 4, 22, 15, 287 and 59 putative targets were obtained from BATMAN-TCM, SwissTargetPrediction, HERB, PharmMapper and TargetNet, respectively. All target genes were unified into standard human gene symbols. After data integration and manual deduplication, a total of 347 non-redundant targets of isoquercitrin were acquired for subsequent bioinformatics analysis.

### Collection of OA and mitochondrial function-related genes

Genes associated with OA were retrieved from GeneCards (https://www.genecards.org/) and online mendelian inheritance in man (OMIM) (https://omim.org/) databases using the keyword “osteoarthritis”. The acquired gene sets were merged and deduplicated to generate a comprehensive list of OA-related genes. Mitochondrial function-related genes were compiled by combining all mitochondrial-localized protein genes from the MitoCarta3.0 database (https://www.broadinstitute.org/mitocarta) with genes associated with “mitochondrial function” (relevance score ≥ 10) from the GeneCards database. After merging, duplicate entries were eliminated to establish the final set of mitochondrial function-related genes.

### Protein-protein interaction (PPI) network construction

The intersection of IQ, OA, and mitochondrial function-related gene sets was used to screen overlapping targets, and a Venn diagram was plotted with R software (v4.3.3, VennDiagram package). These shared targets were designated as potential mediators of IQ’s OA-modulating effects via mitochondrial function. A PPI network of these targets was built in search tool for the retrieval of interacting genes/proteins (STRING) (https://string-db.org/) and visualized in Cytoscape 3.8.2 (https://cytoscape.org/), with node attributes (size, font size, color depth) proportional to the Degree value to reflect node importance.

### Compound-target-disease network construction

A compound-target-disease network was built in Cytoscape 3.8.2 (https://cytoscape.org/) by integrating IQ, overlapping genes, osteoarthritis, and mitochondrial function data. The maximum clique centrality (MCC) algorithm in CytoHubba was applied to pick out the top 30 core targets of the network. Moreover, molecular complex detection (MCODE) plugin-based module analysis was carried out on the PPI network, with the top three prominent modules selected for in-depth visual assessment.

### Gene ontology (GO) and genes and genomes (KEGG) enrichment analysis

GO and KEGG pathway enrichment analyses were conducted on overlapping targets. GO analysis covered three categories: Biological process (BP), cellular component (CC), and molecular function (MF). The top 10 most significant terms (P < 0.05, ranked by P-value) from each GO category and KEGG analysis were selected for visualization. In the plots, circle size or bar length denotes gene count per term/pathway, and color reflects enrichment statistical significance.

### Molecular dynamics (MD) simulation

Molecular dynamics simulations were performed with Gromacs 2022 (https://www.gromacs.org/). System force field parameters were generated via Gromacs’pdb2gmx tool and the AutoFF web server (https://autoff.chem.ucalgary.ca); the assisted model building with energy refinement 14sb (AMBER14sb) force field was applied to the receptor protein, and general amber force field version 2 (GAFF2) to the ligand. The system was solvated in a cubic transferable intermolecular potential 3-Point (TIP3P) water box with a 1 nm solute margin, and charge neutralized using the gmx genion tool. Long-range electrostatics were treated by the particle mesh ewald (PME) method with a 1.0 nm cutoff, and all bonds constrained via the linear constraint solver (LINCS) algorithm. MD runs used the Verlet leap-frog algorithm with a 2 fs time step. Before production simulation, the system was energy-minimized sequentially: 3000 steepest descent steps and 2000 conjugate gradient steps, with constraints on the solute, then counter-ions, and finally no constraints. Production MD was carried out under a constant number of particles, constant pressure, constant temperature (NPT) ensemble (310 K, 1 bar) for 100 ns. Trajectory analyses were performed using Gromacs tools to compute root-mean-square deviation (RMSD), root-mean-square fluctuation (RMSF), hydrogen bond count, radius of gyration (Rg), and solvent-accessible surface area (SASA).

### Lentiviral transduction

The pLVX-puro empty control vector and pLVX-puro-*AKT1* overexpression lentivirus were synthesized by Shanghai Jima Pharmaceutical Technology Co., Ltd. C28/I2 cells were detached with trypsin-EDTA, resuspended, and seeded in 24-well plates at 1 × 10^5^ cells/well with 500 μL complete medium, then incubated at 37°C, 5% CO_2_ for 16–24 h to reach 40–60% confluence for transduction. For infection, the experimental group was treated with 500 μL fresh complete medium containing lentivirus and Polybrene at a final concentration of 5 μg/mL, while control groups received equal volumes of empty vector lentivirus suspension or uninfected complete medium. After 8–12 h of incubation, virus-containing medium was aspirated and replaced with 1 mL fresh complete medium. At 72 h post-infection, transduced cells were subjected to selection to establish stable cell lines for subsequent assays.

### Statistical analysis

Three biological replicates were set for all experiments, and data were shown as mean ± standard deviation (SD). One-way analysis of variance (ANOVA) combined with Tukey’s post hoc test was applied for multiple group comparisons using GraphPad Prism 9.5.0 for Windows (GraphPad Software, Inc., San Diego, CA, USA; https://www.graphpad.com); significance was defined as *P < 0.05, **P < 0.01, ***P < 0.001, ****P < 0.0001 and #P < 0.05, ##P < 0.01, ###P < 0.001, ####P < 0.0001.

## Results

### IQ rescued IL-1β-impaired chondrocyte proliferation

**Fig 1:**
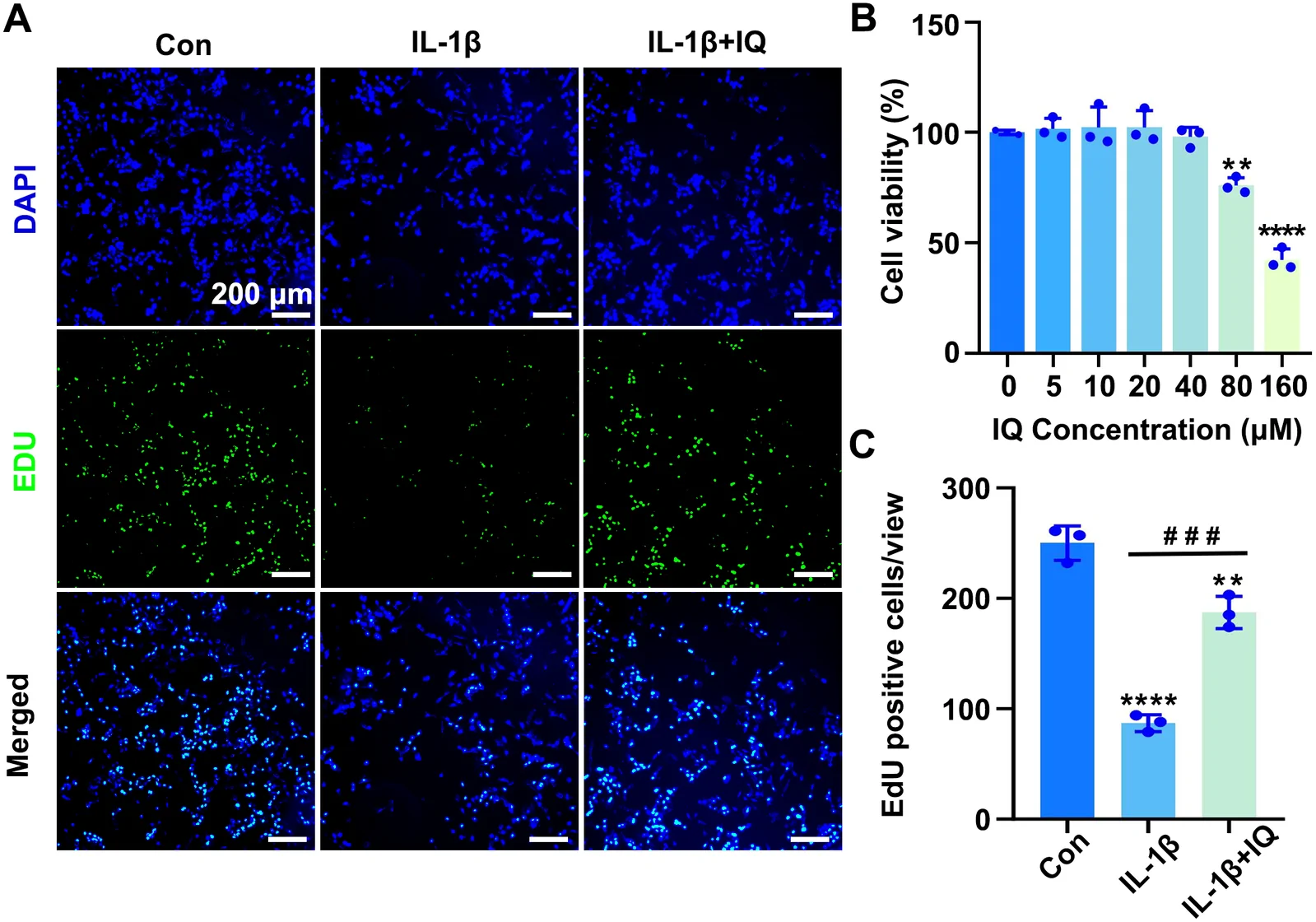
Preliminary chondroprotective evaluation of IQ. **A.** DAPI/EDU co-staining of C28/I2 chondrocytes. DAPI (blue) stains cell nuclei, and EDU (green) labels proliferating cells (scale bar: 200 μm). **B.** Cell viability of C28/I2 chondrocytes treated with gradient concentrations of IQ without IL-1β stimulation. **p < 0.01, ****p < 0.0001 vs. 0 μM group. **C.** Quantification of EdU-positive cells. Data are presented as mean ± standard deviation (SD). **p < 0.01, ****p < 0.0001 vs. Con; ###p < 0.001 vs. IL-1β group. One-way ANOVA with Tukey’s post hoc test was used for multiple comparisons.

To explore the influence of IQ on the proliferation of OA-like chondrocytes, C28/I2 cells were stimulated with 10 ng/mL IL-1β (a standard concentration used in prior studies) to construct an in vitro model [35,36]. EdU immunofluorescence staining was subsequently performed: EdU labeled proliferative cells and DAPI stained cell nuclei. Representative micrographs revealed that IL-1β stimulation led to a dramatic reduction in the number of EdU-positive chondrocytes compared to the control group, while co-treatment with IQ notably reversed this proliferation suppression (Fig 1A).

Prior to functional assays, two independent CCK‑8 viability tests were conducted to identify the optimal IQ dosage. First, broad-range gradient treatment without IL‑1β was used to evaluate intrinsic IQ cytotoxicity. Four‑parameter nonlinear regression fitted an IC₅₀ of 91.76 μM for resting chondrocytes; mild growth inhibition emerged above 80 μM, while concentrations ≤40 μM exerted negligible toxic effects on normal chondrocytes (Fig 1B and S1A, B Fig). Second, we performed a targeted dose–response assay under standardized 10 ng/mL IL‑1β inflammatory stimulation (**S1C Fig**). IL‑1β alone drastically reduced chondrocyte viability. Co‑administration of IQ produced gradual, concentration‑dependent rescue of cell viability from 5 μM up to 40 μM, which yielded the maximum protective effect. However, the protective capacity declined sharply at 80 μM IQ, demonstrating obvious cytotoxicity in the inflammatory microenvironment. Collectively, the dual CCK‑8 datasets confirm that 40 μM IQ achieves maximal chondroprotection without observable toxicity under IL‑1β injury, justifying the use of this concentration for all subsequent functional experiments. Quantitative analysis of EdU-positive cells further validated that IQ significantly attenuated IL-1β-induced proliferation inhibition in a statistically significant manner (Fig 1C). Collectively, these data confirm that the plant-derived, low-toxicity natural compound IQ protects chondrocytes from IL-1β-mediated proliferation impairment, highlighting its promising potential as a safe and effective chondroprotective agent for OA research.

### IQ restores chondrocyte anabolic-catabolic balance and suppresses inflammation under IL-1β stimulation

**Fig 2:**
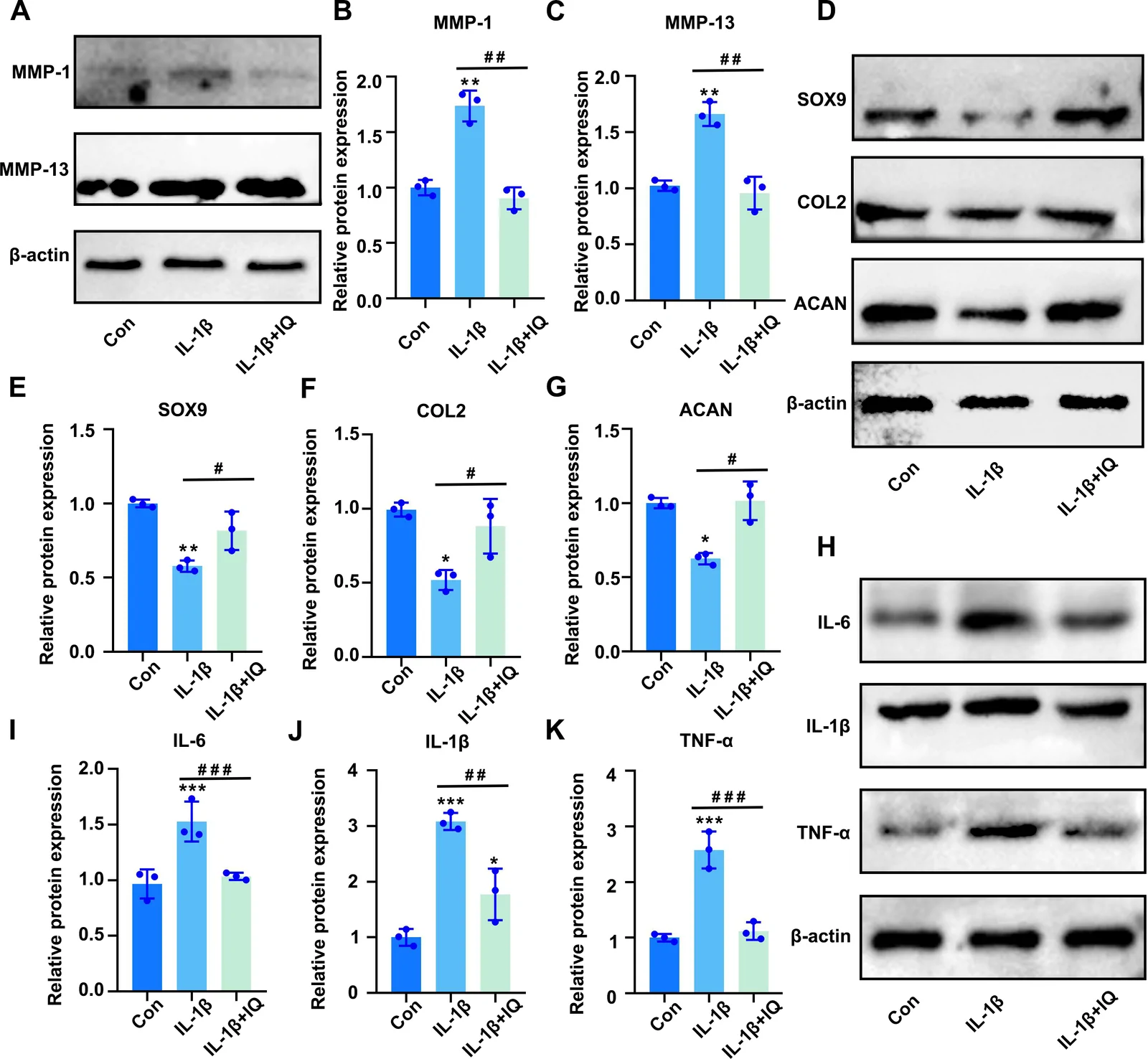
IQ regulates catabolic, anabolic, and inflammatory protein expression in IL-1β-stimulated chondrocytes. **A.** Representative Western blot bands of catabolic enzymes (MMP-1, MMP-13). **B-C.** Quantitative analysis of MMP-1 and MMP-13 protein expression based on Western blot results. **D.** Representative Western blot bands of anabolic matrix markers (SOX9, COL2, ACAN) **E-G.** Quantitative analysis of SOX9, COL2, and ACAN protein expression based on Western blot results. **H.** Representative Western blot bands of pro-inflammatory cytokines (IL-6, IL-1β, TNF-α); β-actin serves as the loading control. **I-K.** Quantitative analysis of IL-6, IL-1β, and TNF-α protein expression based on Western blot results. Data are mean ± SD (n = 3 independent experiments). *p < 0.05, **p < 0.01, ***p < 0.001 vs. Con group; #p < 0.05, ##p < 0.01, ###p < 0.001 vs. IL-1β group. One-way ANOVA with Tukey’s post hoc test was used for multiple comparisons.

Anabolic-catabolic imbalance and excessive inflammation are core drivers of cartilage degradation in OA, with IL-1β acting as a pivotal pro-inflammatory mediator that amplifies catabolic activity while suppressing matrix synthesis [37,38]. We first assessed IQ’s effect on catabolic enzyme expression: Western blotting showed that IL-1β stimulation markedly upregulated matrix metalloproteinase (MMP-1, MMP-13) protein levels—key ECM-degrading enzymes—by ~1.7-fold and ~1.6-fold relative to the control (Con) group, respectively (Figs 2A**-**2C). Notably, IQ co-treatment reversed this induction, reducing MMP-1 and MMP-13 protein levels by ~47.9% and ~42.4% compared to the IL-1β group (approaching control levels). Complementary qRT-PCR (S2A and B Fig) confirmed that IQ similarly downregulated MMP-1/MMP-13 mRNA expression, validating transcriptional regulation of these catabolic factors.

We next evaluated IQ’s impact on anabolic matrix markers critical for cartilage integrity: IL-1β stimulation reduced protein levels of the chondrogenic transcription factor SOX9, COL2 and ACAN by ~42.2%, ~ 47.7%, and ~37.4% relative to the control group, respectively (Figs 2D-2G). In contrast, IQ co-treatment rescued their expression: SOX9, COL2, and ACAN protein levels increased by ~41.4%, ~ 70.0%, and ~62.2% compared to the IL-1β group, restoring near-physiological levels. qRT-PCR further confirmed that IQ upregulated SOX9/COL2/ACAN mRNA expression (S3A-C Fig), consistent with protein-level changes.

Finally, we examined IQ’s effect on pro-inflammatory cytokine production: IL-1β stimulation elevated IL-6, IL-1β, and TNF-α protein levels by ~1.5-fold, ~ 3.1-fold, and ~2.6-fold relative to the control group (Figs 2H-2K). IQ co-treatment significantly suppressed this upregulation, reducing cytokine protein levels by ~78.0%, ~ 42.6%, and ~56.5% compared to the IL-1β group. qRT-PCR (S4A-C Fig) corroborated these findings, showing reduced cytokine mRNA expression in IQ-treated cells. These results demonstrate that IQ exerts dual chondroprotective effects: blunting IL-1β-induced catabolic enzyme and pro-inflammatory cytokine expression, while rescuing anabolic matrix marker levels—supported by both protein and mRNA evidence—highlighting its potential to target multiple pathological pathways in OA.

### IQ restores mitochondrial dynamic balance and preserves membrane potential in IL-1β-stimulated chondrocytes

**Fig 3:**
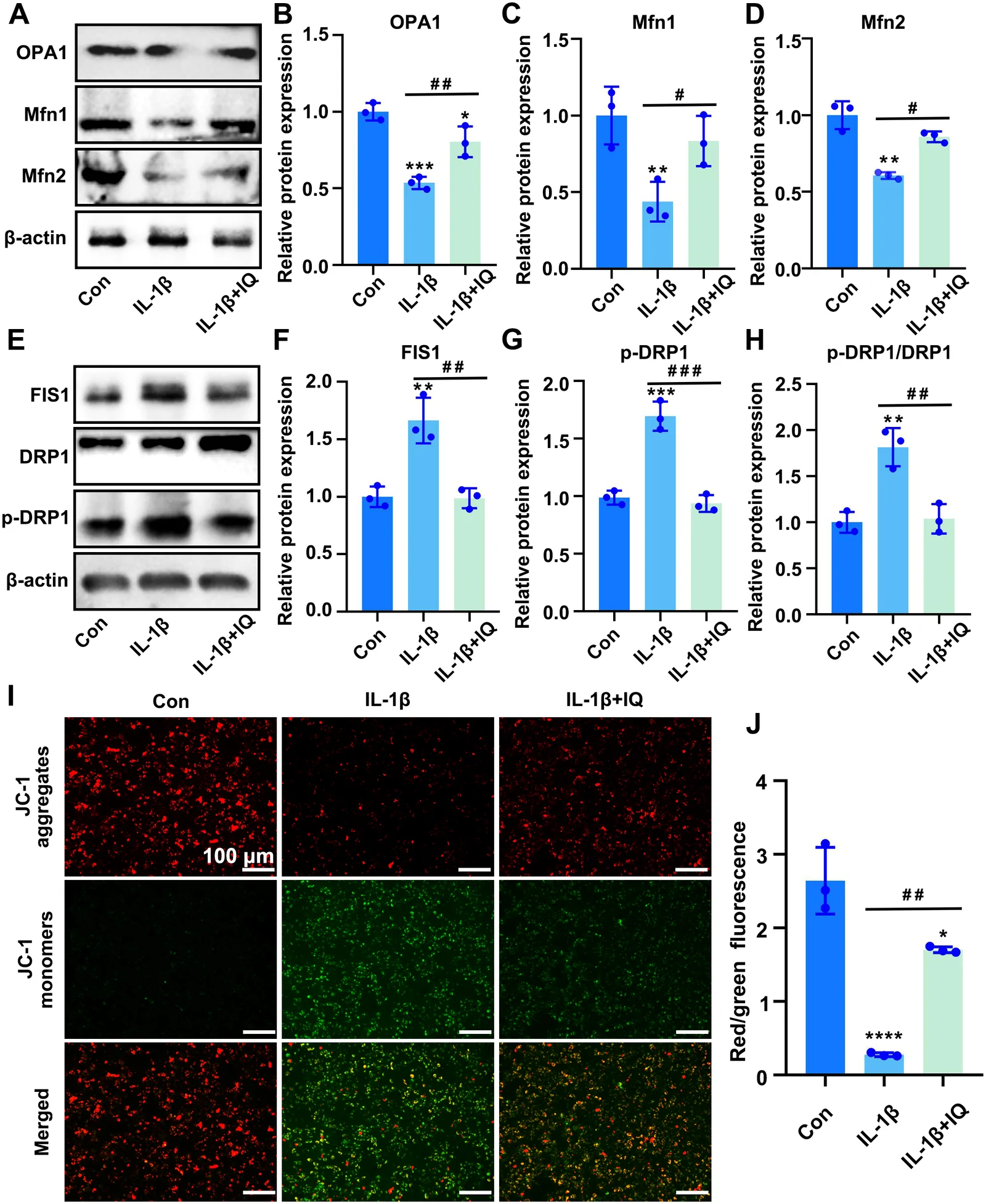
IQ restores mitochondrial dynamics and membrane potential in IL-1β-stimulated chondrocytes. **A.** Representative Western blot bands of mitochondrial fusion proteins (OPA1, Mfn1, Mfn2). **B-D.** Quantitative analysis of OPA1, Mfn1, and Mfn2 protein expression based on Western blot results. **E.** Representative Western blot bands of mitochondrial fission-related proteins (FIS1, DRP1, p-DRP1); **F-H.** Quantitative analysis of FIS1, DRP1 protein expression, and the p-DRP1/DRP1 ratio based on Western blot results. **I.** JC-1 staining of C28/I2 chondrocytes to detect mitochondrial membrane potential (scale bar: 100 μm). Red fluorescence indicates JC-1 aggregates, and green fluorescence indicates JC-1 monomers. **J.** Quantitative analysis of the red/green fluorescence ratio based on JC-1 staining results. Data are mean ± SD (n = 3 independent experiments). *p < 0.05, **p < 0.01, ***p < 0.001, ****p < 0.0001 vs. Con group; #p < 0.05, ##p < 0.01, ###p < 0.001 vs. IL-1β group. One-way ANOVA with Tukey’s post hoc test was used for multiple comparisons.

Mitochondrial dynamic dysregulation drives chondrocyte metabolic dysfunction in OA, with IL-1β acting as a key inducer of this pathological state. We first assessed IQ’s effect on mitochondrial fusion regulators: Western blotting showed that IL-1β stimulation reduced protein levels of the inner-membrane fusion protein OPA1 and outer-membrane fusion proteins Mfn1/Mfn2 by ~46.4%, ~ 56.1%, and ~39.4% relative to the control (Con) group, respectively (Figs 3A**-**3D). Notably, IQ co-treatment rescued these fusion-related proteins: OPA1, Mfn1, and Mfn2 levels increased by ~50.2%, ~ 90.3%, and ~42.9% compared to the IL-1β group, approaching baseline control levels. Complementary qRT-PCR (S5A-C Fig) confirmed that IQ upregulated OPA1/Mfn1/Mfn2 mRNA expression, validating transcriptional control of these fusion mediators.

We next evaluated IQ’s effect on mitochondrial fission factors. IL-1β significantly upregulated the fission proteins FIS1 and p-DRP1, and increased DRP1 phosphorylation (p-DRP1/DRP) by ~1.7–1.8-fold (Figs 3E-H). IQ markedly reversed these changes, reducing FIS1, p-DRP1, and p-DRP1/DRP1 levels by ~40.6%, ~ 42.8%, and ~44.8%, respectively. qRT-PCR confirmed IQ-mediated downregulation of FIS1 mRNA (S5D Fig), indicating that IQ suppresses excessive mitochondrial fission in inflamed chondrocytes. To further clarify the optimal working concentration of IQ in regulating mitochondrial fission, we established a 3-point dose-response model under IL‑1β stimulation (S6 Fig). Treatment with IQ dose-dependently suppressed IL‑1β-triggered DRP1 activation. The maximal inhibitory effect was observed at 40 μM IQ, whereas the protective effect was partially weakened at 80 μM IQ. Consistent with our CCK‑8 cell viability data, these results confirm that 40 μM IQ is the optimal concentration to restrain excessive DRP1 phosphorylation.

To directly assess mitochondrial functional integrity, we measured mitochondrial membrane potential (ΔΨm), a core indicator of mitochondrial health via JC-1 staining (red aggregates = intact ΔΨm; green monomers = depolarized ΔΨm). Representative images showed that IL-1β stimulation drastically reduced JC-1 aggregates (and increased green monomers) relative to the control group, indicating severe mitochondrial depolarization (Fig 3I). IQ co-treatment restored JC-1 aggregate signals, and quantitative analysis of the red/green fluorescence ratio confirmed a significant recovery of ΔΨm (Fig 3J). These results demonstrate that IQ rebalances mitochondrial dynamics in OA chondrocytes: rescuing fusion-related protein expression while suppressing fission factor activation, thereby preserving mitochondrial membrane potential. This mitochondrial protective effect further supports IQ’s role in mitigating chondrocyte metabolic dysfunction in OA.

### Bioinformatic analysis identifies *AKT1* as a key target linking IQ to OA and mitochondrial function

**Fig 4:**
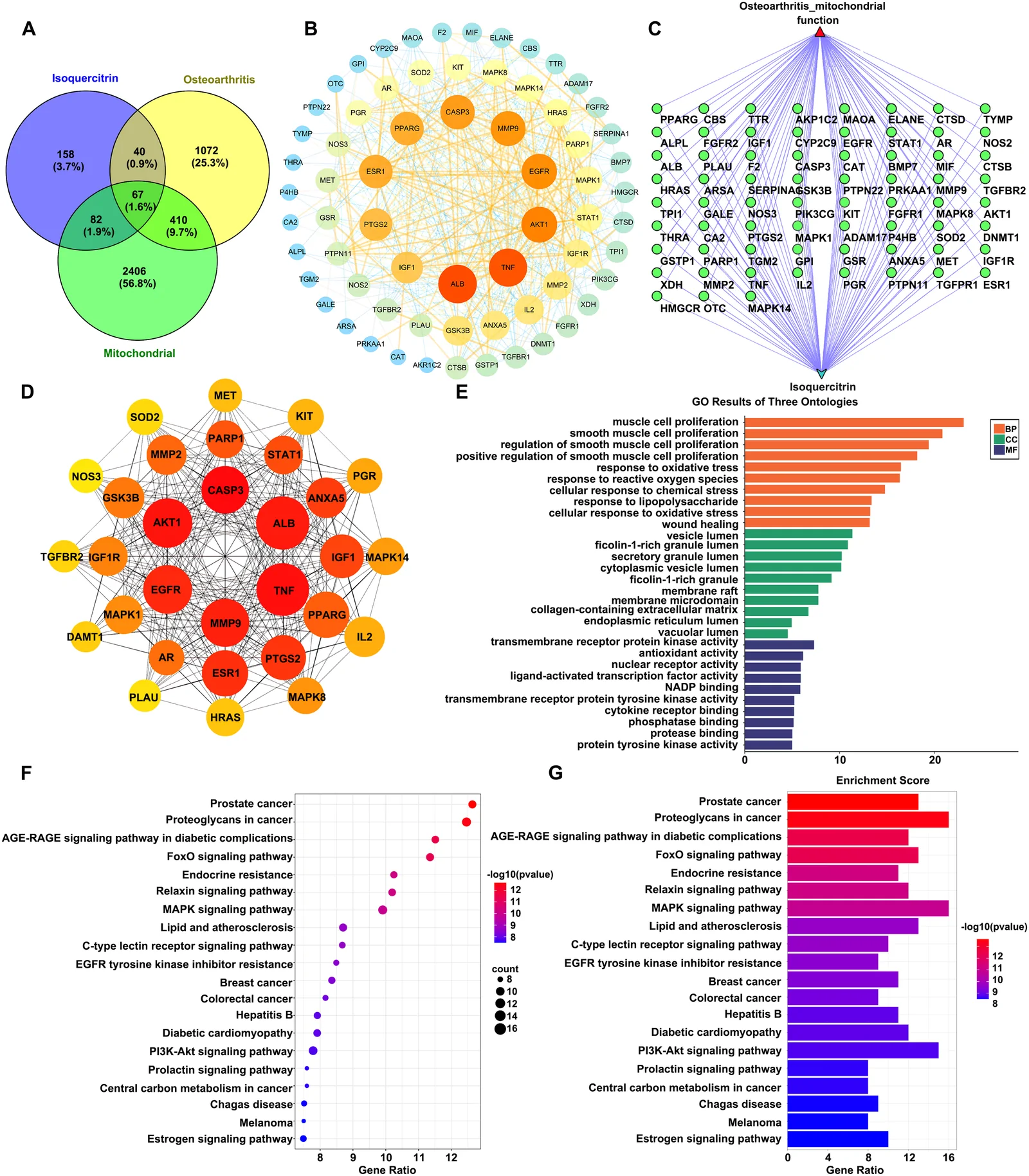
Bioinformatics analysis of potential targets and functional pathways of IQ in osteoarthritis. **A.** Venn diagram showing overlapping targets among IQ-related targets, OA-related targets, and mitochondrial function-related targets. **B.** PPI network of the identified common targets. Nodes represent targets, and edges represent protein-protein interactions. **C.** Visualization of key core nodes in the PPI network. **D.** Cluster analysis of the PPI network, grouping targets into functional modules. **E.** GO enrichment analysis showing top enriched biological processes, cellular components, and molecular functions. **F-G.** KEGG pathway enrichment analysis presented as a scatter plot **F** and bar plot **G**.

To elucidate the molecular targets and pathways underlying IQ’s chondroprotective effects, we performed bioinformatic analysis integrating IQ-related targets, OA-associated genes, and mitochondrial function-related genes. First, a Venn diagram showed 67 overlapping targets among IQ, OA, and mitochondrial function (Fig 4A), accounting for 1.6% of the total intersecting gene set—directly supporting a functional link between IQ, OA pathogenesis, and mitochondrial regulation.

We next constructed a PPI network of these 67 overlapping targets: the network (Fig 4B) contained 67 nodes and 647 interaction edges, with albumin (*ALB*), tumor necrosis factor (*TNF*), *AKT1*, epidermal growth factor receptor (*EGFR*), matrix metallopeptidase 9 (*MMP9*), and caspase 3 (*CASP3*) identified as high-connectivity hub genes. Further screening of core targets via the MCC algorithm (Figs 4C and D) confirmed *CASP3*, *TNF*, *AKT1*, *ALB*, *MMP9*, and *EGFR* as top pivotal nodes, with *AKT1* serving as a key central mediator.

GO enrichment analysis (Fig 4E) generated 1985 total terms, and these targets were primarily enriched in biological processes (e.g., response to lipopolysaccharide, cell proliferation regulation), cellular components (e.g., plasma membrane, mitochondrial inner membrane), and molecular functions (e.g., protein kinase activity, cytokine receptor binding)—processes closely aligned with OA pathogenesis and mitochondrial function regulation.

KEGG pathway enrichment analysis (Figs 4F and G) identified 160 enriched pathways, with prominent terms including “AGE-RAGE signaling pathway in diabetic complications”, “FoxO signaling pathway”, “MAPK signaling pathway”, and “Proteoglycans in cancer”, all of which are implicated in chondrocyte dysfunction, inflammatory response, and mitochondrial homeostasis. Notably, *AKT1* was enriched in multiple OA-relevant pathways, which aligns with our prior functional data on IQ’s regulation of chondrogenic markers and mitochondrial dynamics. These bioinformatic results collectively identify *AKT1* (alongside *TNF*, *ALB*, and *CASP3*) as pivotal hub targets linking IQ to OA and mitochondrial function, providing a precise molecular framework for subsequent mechanistic validation.

### Molecular dynamics simulations validate AKT1 as a stable, high-affinity binding target of IQ among its candidate proteins

**Fig 5:**
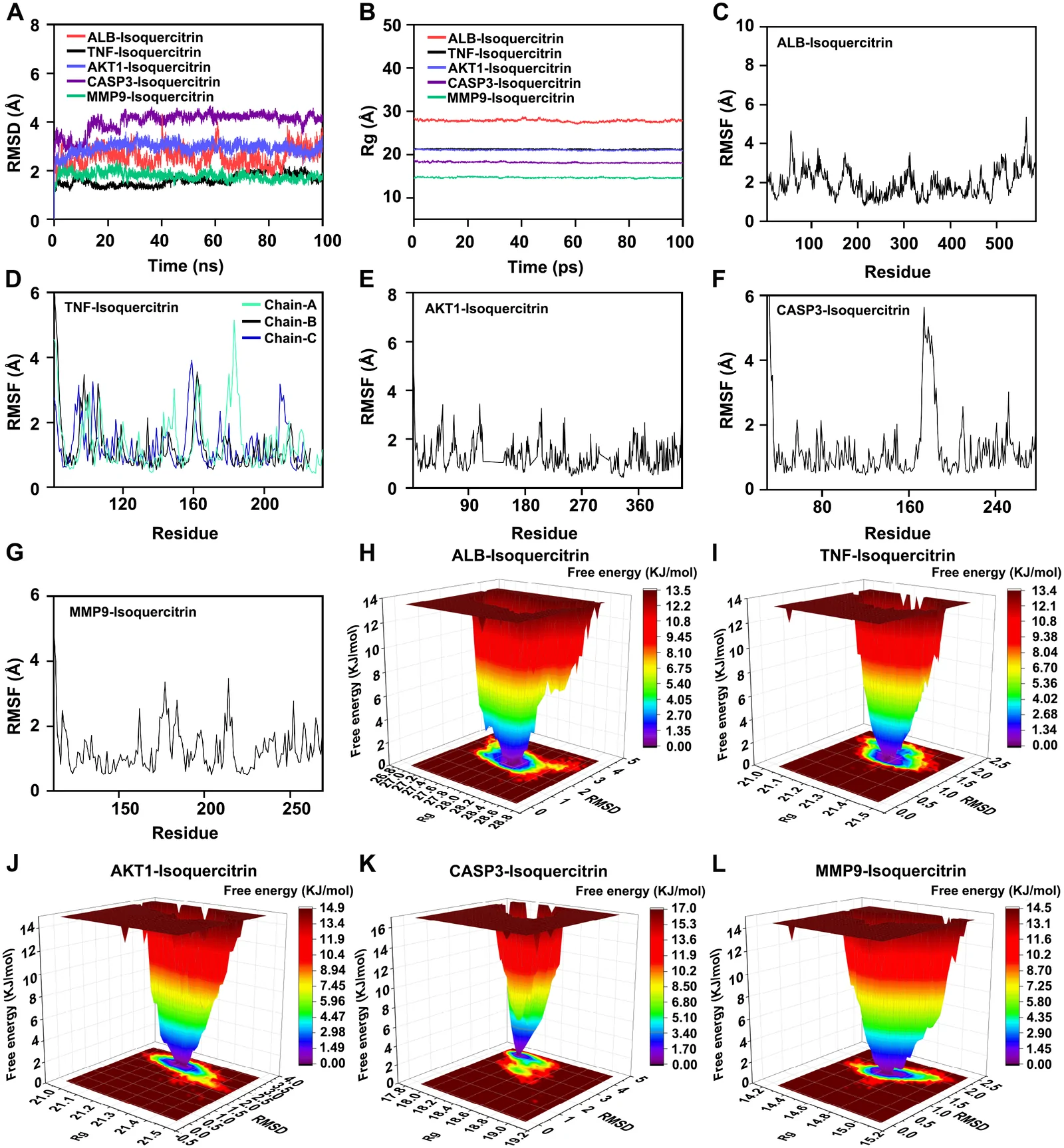
Molecular dynamics simulation analysis of IQ binding to candidate targets. **A.** Root-mean-square deviation curves of IQ complexed with ALB, *AKT1*, TNF, CASP3, and MMP9 over a 100 ns simulation period. **B.** Rg curves of IQ-target complexes during the 100 ns simulation. **C-G.** Root-mean-square fluctuation plots of amino acid residues in ALB, *AKT1*, TNF, CASP3, and MMP9 when complexed with IQ. **H-L.** Free energy landscape (FEL) of IQ-target complexes, visualized based on simulation results.

We evaluated conformational and energetic stability via 100 ns molecular dynamics simulations. Using RMSD, a key metric for conformational stability, we found all complexes reached stable states: ALB-IQ showed minor fluctuations before equilibrating at 85 ns (~ 3.4 Å), TNF-IQ equilibrated rapidly at 10 ns (~ 1.6 Å), AKT1-IQ also equilibrated at 10 ns (~ 3.0 Å), CASP3-IQ remained stable between 40–90 ns (~ 4.1 Å), and MMP9-IQ equilibrated within 5 ns (~ 1.8 Å) (Fig 5A). Complementary Rg analysis revealed stable fluctuations across all complexes, confirming IQ binding did not disrupt the global fold of target proteins (Fig 5B). RMSF further showed AKT1-IQ had mild, uniform residue flexibility (mostly <4 Å) indicative of a stable interface, while CASP3-IQ displayed pronounced flexibility at specific residues (e.g., ~ 160) that hinted at a more dynamic interaction (**Figs 5C-G**). Binding free energy landscapes reinforced energetic favorability (Figs 5H**-**5L): TNF-IQ had the lowest free energy (~1.25 kJ/mol), and AKT1-IQ also displayed a relatively low value (~1.45 kJ/mol), aligning with its high docking affinity.

Finally, we assessed structural interface compactness and interaction durability. The SASA revealed MMP9-IQ (MD target) had the smallest SASA (~10,000 Å²), indicating a tightly packed interface; AKT1-IQ maintained a moderate, stable SASA (~20,000 Å²) (S7A Fig). Hydrogen bond (HBond) analysis revealed CASP3-IQ formed abundant but variable bonds (0–12, averaging ~7), whereas AKT1-IQ sustained a steady, moderate number (0–7, averaging ~4), reinforcing the durability of its binding interface (S7B Fig). Collectively, these results demonstrate that IQ forms stable, energetically favorable complexes with all candidate targets. Notably, AKT1-IQ balances high binding affinity, conformational stability, and durable interface interactions—solidifying its role as a key binding target of IQ

### *AKT1* overexpression reverses IQ’s regulatory effects on chondrocyte function

**Fig 6:**
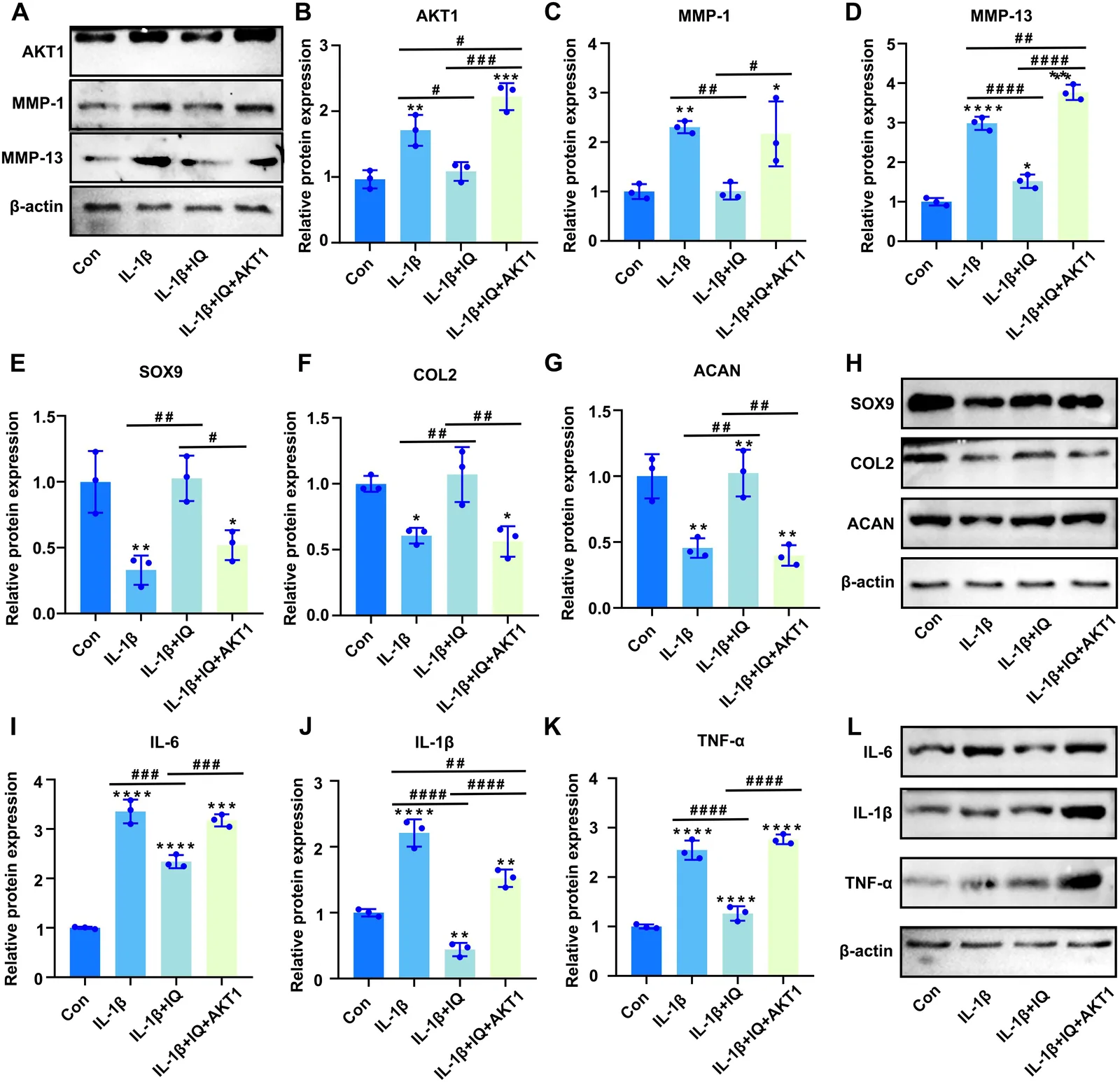
*AKT1* overexpression abrogates IQ-mediated regulation of chondrocyte catabolism, anabolism, and inflammation. **A.** Representative Western blot bands of catabolic enzymes (AKT1, MMP-1, MMP-13). **B-D.** Quantitative analysis of AKT1, MMP-1 and MMP-13 protein expression based on Western blot results. **E-G.** Quantitative analysis of SOX9, COL2, and ACAN protein expression based on Western blot results. **H.** Representative Western blot bands of anabolic matrix markers (SOX9, COL2, ACAN). **I-K.** Quantitative analysis of IL-6, IL-1β, and TNF-α protein expression based on Western blot results. **L.** Representative Western blot bands of pro-inflammatory cytokines (IL-6, IL-1β, TNF-α). Data are mean ± SD (n = 3 independent experiments). *p < 0.05, **p < 0.01, ***p < 0.001, ****p < 0.0001 vs. Con group; #p < 0.05, ##p < 0.01, ###p < 0.001, ####p < 0.0001, as indicated by the horizontal line connecting the two groups being compared. One-way ANOVA with Tukey’s post hoc test was used for multiple comparisons.

To validate that IQ exerts its chondroprotective effects via *AKT1*, we performed rescue experiments with *AKT1* overexpression in IL-1β-stimulated C28/I2 chondrocytes. First, we confirmed the efficiency of lentivirus-mediated *AKT1* overexpression: both Western blot (Figs 6A and B) and qRT-PCR (S8A Fig) results showed that AKT1 protein and mRNA levels were significantly upregulated in the IL-1β + IQ + AKT1 group compared with the Con, IL-1β, and IL-1β + IQ groups, while endogenous AKT1 expression was not significantly altered by IL-1β stimulation or IQ treatment alone.

We then assessed catabolic enzyme expression: IL-1β stimulation significantly elevated MMP-1 and MMP-13 protein (Figs 6A, 6C and 6D) and mRNA levels (S8B **and** C Fig), which were suppressed by IQ treatment. However, *AKT1* overexpression reversed IQ’s inhibitory effect, restoring MMP-1/MMP-13 to near IL-1β-induced levels. Next, we evaluated anabolic matrix markers: IL-1β reduced SOX9, COL2, and ACAN protein (Figs 6E**-**6H) and mRNA levels (S9A-C Fig), which were rescued by IQ. *AKT1* overexpression abrogated this rescue, decreasing SOX9/COL2/ACAN back to IL-1β-reduced levels.

Finally, we measured pro-inflammatory cytokine expression: IL-1β elevated IL-6, IL-1β, and TNF-α protein (Figs 6I**-**6L) and mRNA levels (S10A-C Fig), which were suppressed by IQ. *AKT1* overexpression reversed IQ’s inhibition, restoring cytokine levels to near IL-1β-induced levels. These results collectively confirm that IQ regulates chondrocyte catabolism, anabolism, and inflammation by targeting *AKT1*, with *AKT1* overexpression abrogating IQ’s protective effects. Here we analyzed intracellular cytokine expression to reflect inflammatory gene synthesis, and related secretory function will be explored in our future research.

### *AKT1* overexpression abrogates IQ’s regulation of mitochondrial dynamics and mitochondrial fission in chondrocytes

**Fig 7:**
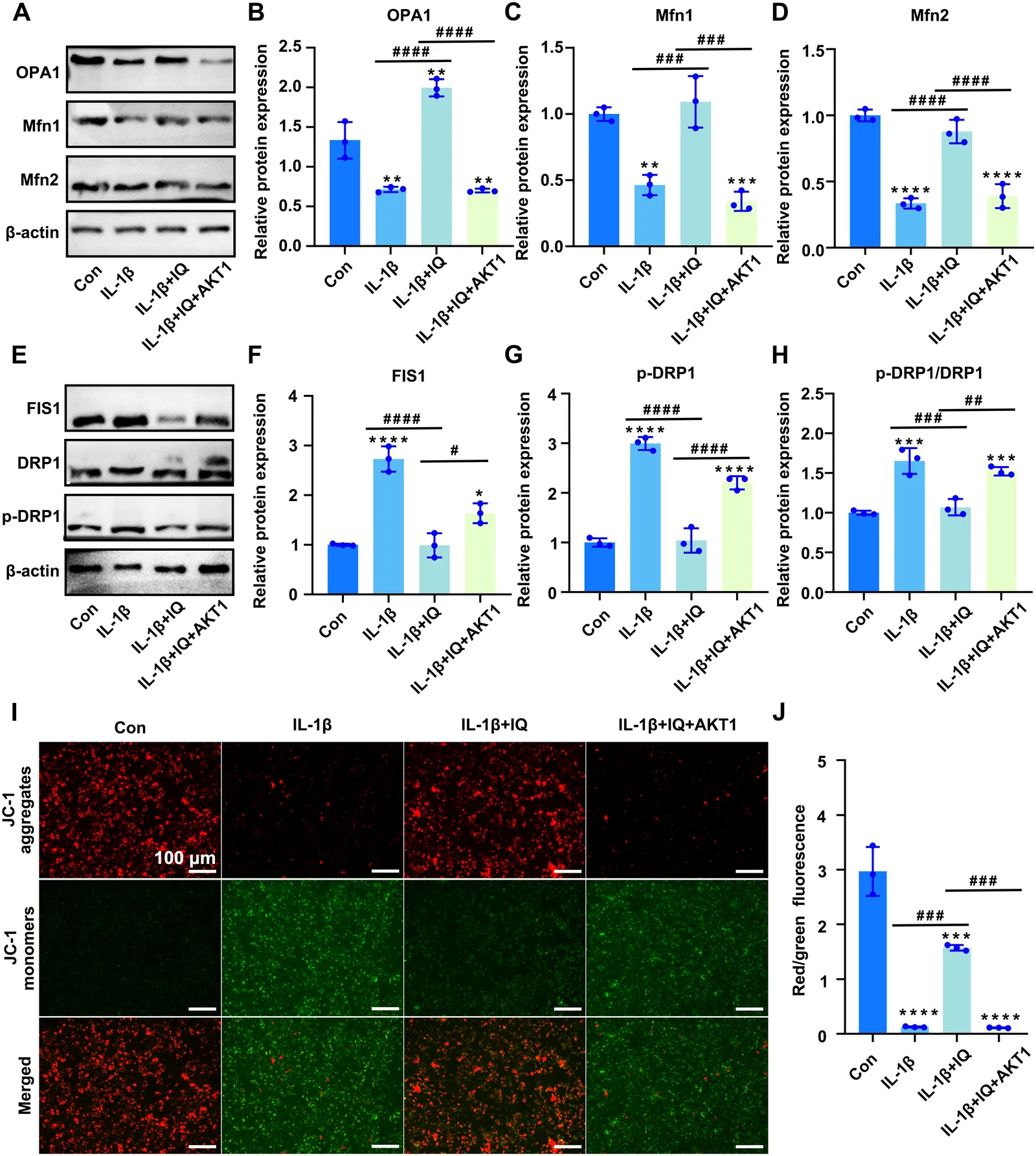
Detection of mitochondrial dynamics-related proteins and membrane potential in IL-1β-stimulated chondrocytes with IQ treatment and *AKT1* overexpression. **A**. Representative Western blot bands of mitochondrial fusion proteins (OPA1, Mfn1, Mfn2). **B-D**. Quantitative analysis of OPA1, Mfn1, and Mfn2 protein expression based on Western blot results. **E.** Representative Western blot bands of mitochondrial fission protein (FIS1, DRP1 and p-DRP1). **F.** Quantitative analysis of FIS1 protein expression based on Western blot results. **G**. Representative Western blot bands of p-DRP1. **H**. Quantitative analysis of the p-DRP1/DRP1 ratio based on Western blot results. Note: Total DRP1 appeared as a doublet band on immunoblots. Both upper and lower bands were enclosed in one ROI and their total integrated density was summed for quantification. **I**. JC-1 staining of chondrocytes to detect mitochondrial membrane potential (scale bar: 100 μm). Red fluorescence indicates JC-1 aggregates, and green fluorescence indicates JC-1 monomers. **J**. Quantitative analysis of the red/green fluorescence ratio based on JC-1 staining results. Data are presented as mean ± SD (n = 3 independent experiments). *p < 0.05, **p < 0.01, ***p < 0.001, ****p < 0.0001 vs. Con group; #p < 0.05, ##p < 0.01, ###p < 0.001, ####p < 0.0001, as indicated by the horizontal line connecting the two groups being compared. One-way ANOVA with Tukey’s post hoc test was used for multiple comparisons.

To investigate whether IQ modulates mitochondrial function via *AKT1*, we assessed mitochondrial dynamics-related proteins and mitochondrial fission in IL-1β-stimulated C28/I2 chondrocytes. First, we measured mitochondrial fusion proteins: IL-1β stimulation reduced OPA1, Mfn1, and Mfn2 protein (Figs 7A**-**7D) and mRNA levels (S11A-C Fig), which were rescued by IQ treatment. However, *AKT1* overexpression reversed this rescue, decreasing OPA1/Mfn1/Mfn2 back to IL-1β-reduced levels (ns vs IL-1β group for Mfn1/Mfn2). For the mitochondrial fission protein FIS1: IL-1β elevated FIS1 protein (Figs 7E and F) and mRNA levels (S11D Fig), which were suppressed by IQ; *AKT1* overexpression restored FIS1 to near IL-1β-induced levels.

We next evaluated mitochondrial fission-related markers: IL-1β increased p-DRP1 and the p-DRP1/DRP1 ratio (Figs 7G and H), which were reduced by IQ. *AKT1* overexpression reversed this inhibition, restoring p-DRP1 and the p-DRP1/DRP1 ratio to IL-1β-induced levels. JC-1 staining (Figs 7I and J) further confirmed this: IL-1β disrupted mitochondrial membrane potential, which was rescued by IQ; *AKT1* overexpression abrogated this rescue.

To directly assess mitochondrial oxidative DNA damage, we performed immunofluorescence co-localization staining for 8-OHdG (a marker of oxidative DNA damage) and TOM20 (a mitochondrial membrane marker, S12 Fig). Strong 8-OHdG fluorescence was observed in IL-1β-treated cells, with prominent co-localization with TOM20, indicating severe mitochondrial oxidative damage. IQ treatment significantly reduced 8-OHdG signals and their co-localization with TOM20, confirming that IQ attenuates IL-1β-induced mtDNA oxidation. However, *AKT1* overexpression reversed these protective effects, restoring high levels of 8-OHdG and its co-localization with TOM20. These results demonstrate that IQ regulates mitochondrial fusion, fission, and membrane potential via *AKT1*, with *AKT1* overexpression reversing IQ’s protective effects on mitochondrial function.

## Discussion

OA is increasingly recognized as a whole-joint disease, involving not only articular cartilage but also the synovium, subchondral bone, ligaments, and periarticular muscles [39,40]. The pathological remodeling of these tissues—synovial inflammation, osteophyte formation, subchondral bone sclerosis, and ligamentous laxity—collectively drives pain and functional decline [41]. Our study focuses on chondrocytes as a central cellular target, but we acknowledge that effective disease-modifying OA therapies must ultimately address this multi-tissue pathology. While IQ demonstrates pronounced chondroprotective effects in vitro, its potential impact on synovial fibroblasts (which contribute to inflammatory cytokine production) or osteoclast/osteoblast activity in subchondral bone remains unexplored. Future studies should evaluate IQ’s effects in ex vivo joint explant models or in vivo OA models that recapitulate this complex tissue crosstalk.

The present work reveals that IQ restores chondrocyte functional homeostasis through a triple mechanism: (1) reversing IL-1β-induced proliferation arrest and shifting metabolism toward anabolism, (2) mitigating pro-inflammatory responses, and (3) normalizing mitochondrial dynamics. Critically, we identify *AKT1* as a central node mediating these effects. Molecular docking predicts high-affinity binding of IQ to AKT1 (−10.4 kcal/mol), and *AKT1* overexpression abrogates IQ’s protective benefits, confirming its necessity. However, the precise mechanism by which AKT1 coordinates mitochondrial dynamics deserves further elaboration. AKT1 bidirectionally regulates DRP1 phosphorylation: Ser616 modification promotes mitochondrial fission, while Ser637 modification restrains it, with the net effect dependent on cellular pathological status [42]. In our IL-1β-stimulated OA model, chronic inflammation triggers sustained aberrant AKT1 hyperactivation, upregulating DRP1 Ser616 phosphorylation and driving excessive pathological mitochondrial fission. IQ binds AKT1 to function as an allosteric modulator that dampens its pathological overactivation to balance mitochondrial fission and fusion. This molecular pathway accounts for the recovered mitochondrial membrane potential observed after IQ treatment. The excess unbound *AKT1* from lentiviral overexpression exceeds IQ’s binding saturation limit, retaining pathological hyperactivity and eliminating IQ’s protective capacity, further confirming that IQ acts to normalize overactive AKT1.

Oxidative stress is a hallmark of OA chondrocytes, driven by mitochondrial dysfunction and nicotinamide adenine dinucleotide phosphate (NADPH) oxidase (NOX) activation [43,44]. We did not directly measure ROS levels in this study, but the literature strongly suggests that IQ is a potent antioxidant. Quercetin, the aglycone of IQ, directly scavenges ROS and upregulates nuclear factor erythroid 2-related factor 2/ antioxidant response element (Nrf2/ARE) pathway, while IQ itself reduces ROS in other cell types (e.g., hepatocytes, neurons) [25,45,46]. Given that IL-1β elevates ROS via NOX4 and mitochondrial electron transport chain leakage, IQ’s mitochondrial-stabilizing effect likely attenuates ROS production indirectly [46]. Furthermore, IQ binds AKT1 as an allosteric buffer to dampen pathological AKT1 hyperactivity and reset AKT1 signaling to physiological levels. Therefore, IQ may suppress ROS through both direct scavenging and AKT1-mediated Nrf2 activation. Future experiments should quantify intracellular ROS (e.g., 2′,7′-Dichlorodihydrofluorescein diacetate (DCFH-DA) staining) and examine Nrf2 pathway activation (e.g., nuclear translocation, ARE-luciferase reporter) in IQ-treated chondrocytes.

In conclusion, this study establishes IQ as a promising candidate for OA intervention by targeting AKT1 to restore chondrocyte anabolic-catabolic balance, suppress inflammation, and stabilize mitochondrial dynamics. The multi-target nature of IQ—acting on proliferation, metabolism, inflammation, and mitochondria—offers advantages over single-target agents. One key limitation of the current work is the absence of an IQ single-treatment experimental group to independently dissect its standalone biological activity; subsequent follow-up work will supplement detections of ECM, inflammatory and mitochondrial markers under IQ monotherapy to fully clarify its intrinsic regulatory effects. However, translation to clinical application requires several steps: (1) evaluation of IQ bioavailability and joint pharmacokinetics, (2) assessment of efficacy in post-traumatic and age-related OA animal models (e.g., destabilization of the medial meniscus (DMM) or monosodium iodoacetate (MIA)-induced rats), and (3) investigation of potential off-target effects on other joint tissues. Our findings provide a mechanistic framework linking AKT1 to chondroprotection, opening avenues for developing AKT1-targeted DMOADs.

## Supporting information

S1 Fig Cytotoxicity and protective effects of IQ on C28/I2 chondrocytes. (A) CCK-8 assay showing the viability of C28/I2 chondrocytes treated with increasing concentrations of IQ (0–160 μM) for 24 h under normal culture conditions. (B) Nonlinear regression analysis determined the half-maximal inhibitory concentration (IC₅₀) of IQ in chondrocytes without inflammatory stimulation. (C) Dose–response curve of IQ in the presence of IL-1β-induced inflammatory injury. Chondrocytes were exposed to 10 ng/mL IL-1β together with serial concentrations of IQ (0, 5, 10, 20, 40, 80 μM) for 24 h. Data represent the mean ± SD of three independent biological replicates. Statistical analysis was performed by one-way ANOVA. **p < 0.01, ***p < 0.001, ****p < 0.0001.
(TIF)

S2 Fig The qRT-PCR analysis of MMP-1 (A) and MMP-13 (B) mRNA levels in C28/I2 chondrocytes. Data are mean ± SD (n = 3 independent experiments), with **P < 0.01, ***P < 0.001vs. Con group; ###p < 0.001 vs. IL-1β group. One – way analysis of variance (ANOVA) with Tukey’s post hoc test was used for multiple comparisons.
(TIF)

S3 Fig The qRT-PCR analysis of anabolic factor mRNA levels in C28/I2 chondrocytes: (A) SOX9 (chondrogenic transcription factor), (B) COL2 (type II collagen), and (C) ACAN (aggrecan). Data are mean ± SD (n = 3 independent experiments), with *P < 0.05, **P < 0.01, ***P < 0.001, ****P < 0.0001vs. Con group; #p < 0.05, ##p < 0.01, ####p < 0.0001 vs. IL-1β group. One – way analysis of variance (ANOVA) with Tukey’s post hoc test was used for multiple comparisons.
(TIF)

S4 Fig The qRT-PCR analysis of pro-inflammatory cytokine mRNA levels in C28/I2 chondrocytes: (A) IL-6, (B) IL-1β, and (C) TNF-α. Data are mean ± SD (n = 3 independent experiments), with **P < 0.01, ***P < 0.001, ****P < 0.0001 vs. Con group; ##p < 0.01, ###p < 0.001, ####p < 0.0001 vs. IL-1β group. One – way analysis of variance (ANOVA) with Tukey’s post hoc test was used for multiple comparisons.
(TIF)

S5 Fig The qRT-PCR analysis of pro-inflammatory cytokine mRNA levels in C28/I2 chondrocytes: (A) OPA1, (B) Mfn1, (C) Mfn2 and (D) FIS1. Data are mean ± SD (n = 3 independent experiments), with *P < 0.05, **P < 0.01, ***P < 0.001, ****P < 0.0001vs. Con group; ##p < 0.01, ###p < 0.001, ####p < 0.0001 vs. IL-1β group. One – way analysis of variance (ANOVA) with Tukey’s post hoc test was used for multiple comparisons.
(TIF)

S6 Fig IQ inhibits IL‑1β‑induced DRP1 phosphorylation in a dose-dependent manner. (A) Western blot bands of p-DRP1 and total DRP1. (B, C) Quantitative analysis of relative p-DRP1 expression and p-DRP1/DRP1 ratio. All samples were loaded on a single gel; sequential stripping and re-probing of the same membrane was performed owing to similar molecular weights of p-DRP1 and DRP1. β‑actin served as internal control. Data are mean ± SD (n = 3). ****p < 0.0001 vs. Con; ##p < 0.01, ###p < 0.001, ####p < 0.0001 vs. IL‑1β + IQ (40 μM) group. One-way ANOVA with Tukey’s test.
(TIF)

S7 Fig Structural metrics of isoquercitrin (IQ)-target complexes. (A) Solvent-accessible surface area (SASA) over 100 ns MD simulation: AKT1-IQ maintained stable, moderate SASA (minimizing interface solvent exposure). (B) Hydrogen bond (HBond) numbers: AKT1-IQ showed consistent HBond formation, while other complexes (e.g., ALB-IQ) had sparse, variable HBonds.
(TIF)

S8 Fig The qRT-PCR analysis of catabolic enzyme mRNA levels in C28/I2 chondrocytes: (A) AKT1, (B) MMP-1 and (C) MMP-13. Data are mean ± SD (n = 3 independent experiments), with **p < 0.01, ****p < 0.0001 vs. Con group; ####p < 0.0001 as indicated by the horizontal line connecting the two groups being compared. One-way analysis of variance (ANOVA) with Tukey’s post hoc test was used for multiple comparisons.
(TIF)

S9 Fig The qRT-PCR analysis of anabolic matrix marker mRNA levels in C28/I2 chondrocytes: (A) SOX9, (B) COL2 and (C) ACAN. Data are mean ± SD (n = 3 independent experiments), with ***p < 0.001, ****p < 0.0001 vs. Con group; ###p < 0.001, ####p < 0.0001 vs. IL-1β + IQ group. One-way analysis of variance (ANOVA) with Tukey’s post hoc test was used for multiple comparisons.
(TIF)

S10 Fig The qRT-PCR analysis of anabolic matrix marker mRNA levels in C28/I2 chondrocytes: (A) IL-6, (B) IL-1β and (C) TNF-α. Data are mean ± SD (n = 3 independent experiments), *p < 0.05, ****p < 0.0001 vs. Con group; ####p < 0.0001 vs. IL-1β + IQ group. One-way analysis of variance (ANOVA) with Tukey’s post hoc test was used for multiple comparisons.
(TIF)

S11 Fig The qRT-PCR analysis of anabolic matrix marker mRNA levels in C28/I2 chondrocytes: (A) OPA1, (B) Mfn1, (C) Mfn2 and (D) FIS1. Data are mean ± SD (n = 3 independent experiments), with***p < 0.001, ****p < 0.0001 vs. Con group; ###p < 0.001, ####p < 0.0001 vs. IL-1β + IQ group. One-way analysis of variance (ANOVA) with Tukey’s post hoc test was used for multiple comparisons.
(TIF)

S12 Fig IQ reduces IL-1β-induced mitochondrial oxidative DNA damage in chondrocytes via AKT1. (A) Representative immunofluorescence images of TOM20 (red, mitochondria), 8-OHdG (green, oxidized DNA), and DAPI (blue, nuclei) in chondrocytes under different treatments. Scale bar: 100 μm. (B) Quantitative analysis of mean 8-OHdG fluorescence intensity. Data are mean ± SD (n = 3 independent experiments), with ****P < 0.0001 vs. Con; ####p < 0.0001 vs. IL-1β + IQ group. One-way analysis of variance (ANOVA) with Tukey’s post hoc test was used for multiple comparisons.
(TIF)

S13 Fig Graphical abstract. (TIF)

S1 Data Raw data. (XLSX)

S2 Data Raw images. (PDF)

## References

[1] Berenbaum F, Walker C. Osteoarthritis and inflammation: a serious disease with overlapping phenotypic patterns. Postgrad Med. 2020;132(4):377–84. doi: 10.1080/00325481.2020.1730669 32100608

[2] Zhang W, Qi L, Chen R, He J, Liu Z, Wang W, et al. Circular RNAs in osteoarthritis: indispensable regulators and novel strategies in clinical implications. Arthritis Res Ther. 2021;23(1):23. doi: 10.1186/s13075-021-02420-2 33436088 PMC7802294

[3] Wakale S, Wu X, Sonar Y, Sun A, Fan X, Crawford R, et al. How are Aging and Osteoarthritis Related?. Aging and disease. 2023;14(3):592. doi: 10.14336/ad.2022.0831 PMC10187698 37191424

[4] Najar M, Fahmi H. Of Mesenchymal Stem/Stromal Cells and Osteoarthritis: Time to Merge the Latest Breakthroughs. Stem Cell Rev Rep. 2020;16(5):1016–8. doi: 10.1007/s12015-020-10001-0 32656622

[5] Pettenuzzo S, Berardo A, Belluzzi E, Pozzuoli A, Ruggieri P, Carniel EL, et al. Mechanical insights into fat pads: a comparative study of infrapatellar and suprapatellar fat pads in osteoarthritis. Connect Tissue Res. 2025;66(4):272–83. doi: 10.1080/03008207.2025.2502591 40340764

[6] Battistelli M, Favero M, Burini D, Trisolino G, Dallari D, De Franceschi L, et al. Morphological and ultrastructural analysis of normal, injured and osteoarthritic human knee menisci. Eur J Histochem. 2019;63(1):2998. doi: 10.4081/ejh.2019.2998 30739432 PMC6379780

[7] Zhu X, Chan YT, Yung PSH, Tuan RS, Jiang Y. Subchondral Bone Remodeling: A Therapeutic Target for Osteoarthritis. Front Cell Dev Biol. 2021;8:607764. doi: 10.3389/fcell.2020.607764 33553146 PMC7859330

[8] Martel-Pelletier J, Maheu E, Pelletier J-P, Alekseeva L, Mkinsi O, Branco J, et al. A new decision tree for diagnosis of osteoarthritis in primary care: international consensus of experts. Aging Clin Exp Res. 2019;31(1):19–30. doi: 10.1007/s40520-018-1077-8 30539541 PMC6514162

[9] Liu S, Zhang G, Li N, Wang Z, Lu L. The interplay of aging and PANoptosis in osteoarthritis pathogenesis: Implications for novel therapeutic strategies. J Inflamm Res. 2025;18:1951–67. doi: 10.2147/JIR.S489613 PMC11829118 39959642

[10] Yadav S, Yadav J, Humphrey MB. Emerging Role of TRP Channels in Osteoarthritis Pathogenesis. Cells. 2026;15(3):299. doi: 10.3390/cells15030299 41677662 PMC12896641

[11] Yang J, Shin Y, Kim H-J, Kim H-E, Chun J-S. Prokineticin 2 is a catabolic regulator of osteoarthritic cartilage destruction in mouse. Arthritis Res Ther. 2023;25(1):236. doi: 10.1186/s13075-023-03206-4 38057865 PMC10699050

[12] Li R, Guan Z, Bi S, Wang F, He L, Niu X, et al. The proton-activated G protein-coupled receptor GPR4 regulates the development of osteoarthritis via modulating CXCL12/CXCR7 signaling. Cell Death Dis. 2022;13(2):152. doi: 10.1038/s41419-021-04455-4 35165253 PMC8844071

[13] Hwang HS, Lee MH, Kim HA. The TLR-2/TonEBP signaling pathway regulates 29-kDa fibronectin fragment-dependent expression of matrix metalloproteinases. Sci Rep. 2021;11(1):8891. doi: 10.1038/s41598-021-87813-8 PMC8076285 33903620

[14] Harvanova D, Matejova J, Slovinska L, Lacko M, Gulova S, Fecskeova LK, et al. The Role of Synovial Membrane in the Development of a Potential In Vitro Model of Osteoarthritis. Int J Mol Sci. 2022;23(5):2475. doi: 10.3390/ijms23052475 35269618 PMC8910122

[15] Mallia I, Fioravanti A, Guiducci S. Infrapatellar Fat Pad in Knee Osteoarthritis: A Comprehensive Review of Pathophysiology and Targeted Therapeutic Strategies. Int J Mol Sci. 2025;26(21):10408. doi: 10.3390/ijms262110408 41226448 PMC12609914

[16] Liu Y, Liu Y, Zhang N, An H, Mi L, Gao Y, et al. Mitochondrial transplantation for osteoarthritis: from molecular mechanisms to clinical translation. Front Immunol. 2026;17:1716906. doi: 10.3389/fimmu.2026.1716906 41948333 PMC13050752

[17] Wei Z, Dong C, Guan L, Wang Y, Huang J, Wen X. A metabolic exploration of the protective effect of Ligusticum wallichii on IL-1β-injured mouse chondrocytes. Chin Med. 2020;15:12. doi: 10.1186/s13020-020-0295-0 32025239 PMC6995652

[18] Ahmed S, Comolli NK. Delivery of potential natural disease-modifying osteoarthritis drugs: Beyond polyphenols and flavonoids. Biomed Pharmacother. 2026;198:119154. doi: 10.1016/j.biopha.2026.119154 41904894

[19] Sukhikh S, Noskova S, Ivanova S, Ulrikh E, Izgaryshev A, Babich O. Chondroprotection and Molecular Mechanism of Action of Phytonutraceuticals on Osteoarthritis. Molecules. 2021;26(8):2391. doi: 10.3390/molecules26082391 33924083 PMC8074261

[20] Mattiuzzo E, Faggian A, Venerando R, Benetti A, Belluzzi E, Abatangelo G, et al. In Vitro Effects of Low Doses of β-Caryophyllene, Ascorbic Acid and d-Glucosamine on Human Chondrocyte Viability and Inflammation. Pharmaceuticals (Basel). 2021;14(3):286. doi: 10.3390/ph14030286 33806983 PMC8005039

[21] Xuan C, Chen D, Zhang S, Li C, Fang Q, Chen D, et al. Isoquercitrin Alleviates Diabetic Nephropathy by Inhibiting STAT3 Phosphorylation and Dimerization. Adv Sci (Weinh). 2025;12(25):e2414587. doi: 10.1002/advs.202414587 40184310 PMC12224983

[22] Chen Y-Y, Guo J, Xue S-L, Ding L-L, Wei B-Y, Wen S-Y. Uncovering the Anti-Atherosclerotic Effect of Isoquercitrin via Microbiome Metabolic Modulation and Network Pharmacology. J Agric Food Chem. 2025;73(37):23469–85. doi: 10.1021/acs.jafc.5c09903 40916588

[23] Huang S-H, Xu M, Wu H-M, Wan C-X, Wang H-B, Wu Q-Q, et al. Isoquercitrin Attenuated Cardiac Dysfunction Via AMPKα-Dependent Pathways in LPS-Treated Mice. Mol Nutr Food Res. 2018;62(24):e1800955. doi: 10.1002/mnfr.201800955 30359483

[24] Iqbal S, Karim MR, Mohammad S, Ahn JC, Kariyarath Valappil A, Mathiyalagan R, et al. In Silico and In Vitro Study of Isoquercitrin against Kidney Cancer and Inflammation by Triggering Potential Gene Targets. Curr Issues Mol Biol. 2024;46(4):3328–41. doi: 10.3390/cimb46040208 38666938 PMC11049307

[25] Kwon S-H, Lee W-Y, Kim YW, Ko KS, Bak SB, Park S-D. Isoquercitrin Attenuates Oxidative Liver Damage Through AMPK-YAP Signaling: An Integrative In Silico, In Vitro, and In Vivo Study. Int J Mol Sci. 2025;26(6):2717. doi: 10.3390/ijms26062717 40141359 PMC11943443

[26] Cho W-K, Lee M-M, Ma JY. Antiviral Effect of Isoquercitrin against Influenza A Viral Infection via Modulating Hemagglutinin and Neuraminidase. Int J Mol Sci. 2022;23(21):13112. doi: 10.3390/ijms232113112 36361900 PMC9653704

[27] Usman A, Işık S, Abba S, Meriçli F. Artificial intelligence-based models for the qualitative and quantitative prediction of aphytochemical compound using HPLC method. Turk J Chem. 2020;44(5):1339–51. doi: 10.3906/kim-2003-6 PMC7751937 33488234

[28] Li X, Zhou D, Yang D, Fu Y, Tao X, Hu X, et al. Isoquercitrin Attenuates Osteogenic Injury in MC3T3 Osteoblastic Cells and the Zebrafish Model via the Keap1-Nrf2-ARE Pathway. Molecules. 2022;27(11):3459. doi: 10.3390/molecules27113459 35684398 PMC9182080

[29] Li J, Wang X, Wang Y, Lu C, Zheng D, Zhang J. Isoquercitrin, a flavonoid glucoside, exerts a positive effect on osteogenesis in vitro and in vivo. Chem Biol Interact. 2019;297:85–94. doi: 10.1016/j.cbi.2018.10.018 30365939

[30] Li H, Chen G, Ma C, Hu Y, Zhou P, Zhou S, et al. Isoquercitrin attenuates osteoarthritis progression by targeting P53-mediated ferroptosis: A mechanistic study integrating network pharmacology and experimental validation. J Nutr Biochem. 2026;153:110306. doi: 10.1016/j.jnutbio.2026.110306 41663037

[31] Manning BD, Toker A. AKT/PKB Signaling: Navigating the Network. Cell. 2017;169(3):381–405. doi: 10.1016/j.cell.2017.04.001 28431241 PMC5546324

[32] Xie J, Lin J, Wei M, Teng Y, He Q, Yang G, et al. Sustained Akt signaling in articular chondrocytes causes osteoarthritis via oxidative stress-induced senescence in mice. Bone Res. 2019;7:23. doi: 10.1038/s41413-019-0062-y 31646013 PMC6804644

[33] Xu C, Wang H, Wang H, Man J, Deng Y, Li Y, et al. Schisandrin B regulates mitochondrial dynamics via AKT1 activation and mitochondrial targeting to ameliorate renal ischemia-reperfusion injury. Phytomedicine. 2025;141:156672. doi: 10.1016/j.phymed.2025.156672 40220406

[34] Schneider CA, Rasband WS, Eliceiri KW. NIH Image to ImageJ: 25 years of image analysis. Nat Methods. 2012;9(7):671–5. doi: 10.1038/nmeth.2089 22930834 PMC5554542

[35] Feng K, Xie X, Yuan J, Gong L, Zhu Z, Zhang J, et al. Reversing the surface charge of MSC-derived small extracellular vesicles by εPL-PEG-DSPE for enhanced osteoarthritis treatment. J Extracell Vesicles. 2021;10(13):e12160. doi: 10.1002/jev2.12160 34724347 PMC8559985

[36] Arra M, Swarnkar G, Ke K, Otero JE, Ying J, Duan X, et al. LDHA-mediated ROS generation in chondrocytes is a potential therapeutic target for osteoarthritis. Nat Commun. 2020;11(1):3427. doi: 10.1038/s41467-020-17242-0 32647171 PMC7347613

[37] Rutgers M, Saris DBF, Dhert WJA, Creemers LB. Cytokine profile of autologous conditioned serum for treatment of osteoarthritis, in vitro effects on cartilage metabolism and intra-articular levels after injection. Arthritis Res Ther. 2010;12(3):R114. doi: 10.1186/ar3050 20537160 PMC2911907

[38] Shih K-S, Wang J-H, Wu Y-W, Teng C-M, Chen C-C, Yang C-R. Aciculatin inhibits granulocyte colony-stimulating factor production by human interleukin 1β-stimulated fibroblast-like synoviocytes. PLoS One. 2012;7(7):e42389. doi: 10.1371/journal.pone.0042389 22860122 PMC3409160

[39] Huang X, Wang Z, Wang H, Chen D, Tong L. Novel strategies for the treatment of osteoarthritis based on biomaterials and critical molecular signaling. J Mater Sci Technol. 2023;149:42–55. doi: 10.1016/j.jmst.2022.11.027

[40] Umar M, Wang Z, Li J, Li Z, Li G, Tong L, et al. Novel therapeutic strategies for osteoarthritis: from mechanistic insights to precision medicine. Bone Res. 2026;14(1):64. doi: 10.1038/s41413-026-00540-6 42276983 PMC13260458

[41] Liao L, Zhang S, Zhao L, Chang X, Han L, Huang J, et al. Acute Synovitis after Trauma Precedes and is Associated with Osteoarthritis Onset and Progression. Int J Biol Sci. 2020;16(6):970–80. doi: 10.7150/ijbs.39015 32140066 PMC7053339

[42] Adhikary A, Mukherjee A, Banerjee R, Nagotu S. DRP1: At the Crossroads of Dysregulated Mitochondrial Dynamics and Altered Cell Signaling in Cancer Cells. ACS Omega. 2023;8(48):45208–23. doi: 10.1021/acsomega.3c06547 38075775 PMC10701729

[43] Liu L, Luo P, Yang M, Wang J, Hou W, Xu P. The role of oxidative stress in the development of knee osteoarthritis: A comprehensive research review. Front Mol Biosci. 2022;9:1001212. doi: 10.3389/fmolb.2022.1001212 36203877 PMC9532006

[44] Han J, Park D, Park JY, Han S. Inhibition of NADPH Oxidases Prevents the Development of Osteoarthritis. Antioxidants. 2022;11(12):2346. doi: 10.3390/antiox11122346 PMC9774355 36552552

[45] Treml J, Leláková V, Šmejkal K, Paulíčková T, Labuda Š, Granica S, et al. Antioxidant Activity of Selected Stilbenoid Derivatives in a Cellular Model System. Biomolecules. 2019;9(9):468. doi: 10.3390/biom9090468 31505897 PMC6770161

[46] Dai Y, Zhang H, Zhang J, Yan M. Isoquercetin attenuates oxidative stress and neuronal apoptosis after ischemia/reperfusion injury via Nrf2-mediated inhibition of the NOX4/ROS/NF-κB pathway. Chem Biol Interact. 2018;284:32–40. doi: 10.1016/j.cbi.2018.02.017 29454613

