# Supplementary material for: Isoquercitrin ameliorates chondrocyte dysfunction in osteoarthritis by targeting AKT1-mediated mitochondrial dynamics regulation
Source: PLoS One. 2026 Sep 17;21(9):e0357989. doi: 10.1371/journal.pone.0357989 (PMC13585277; doi:10.1371/journal.pone.0357989)

Figure 2A

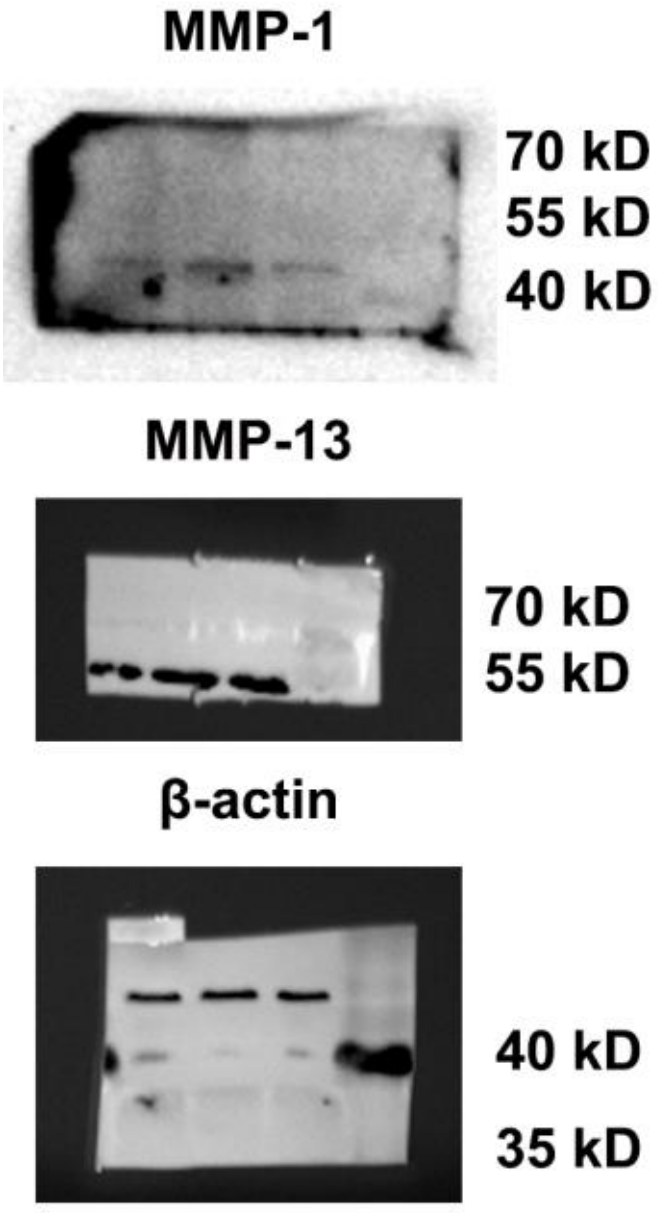

Figure 2D

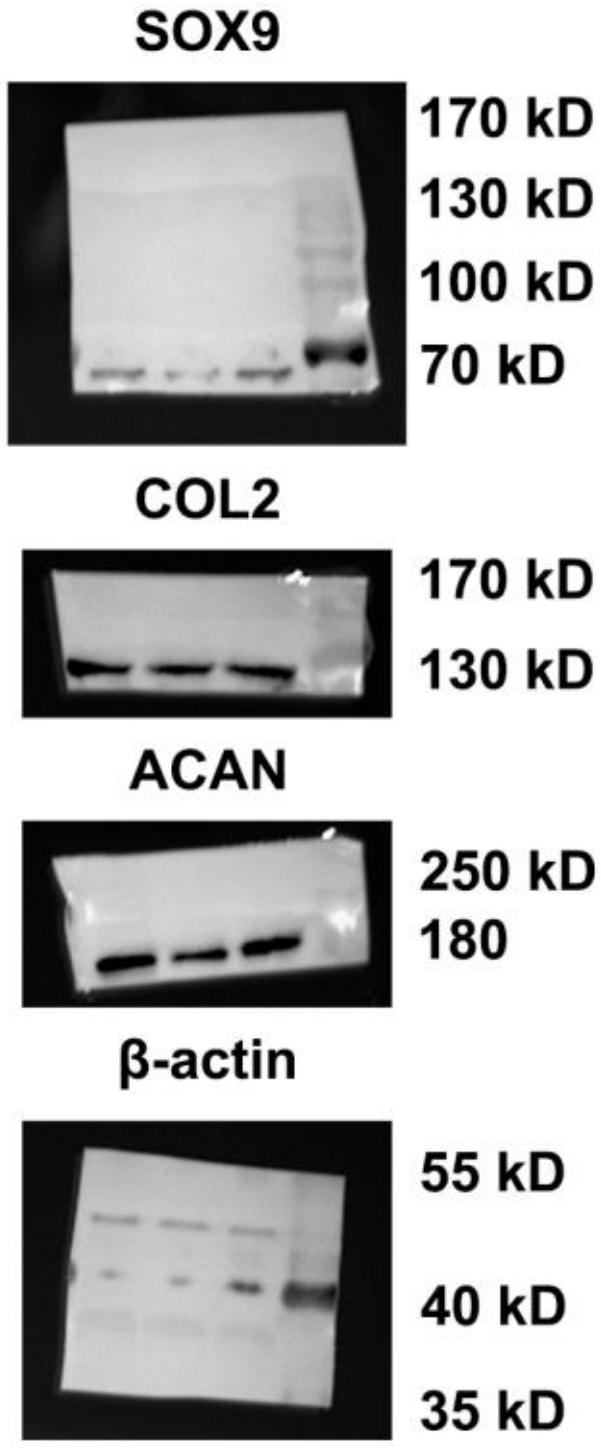

Figure 2H

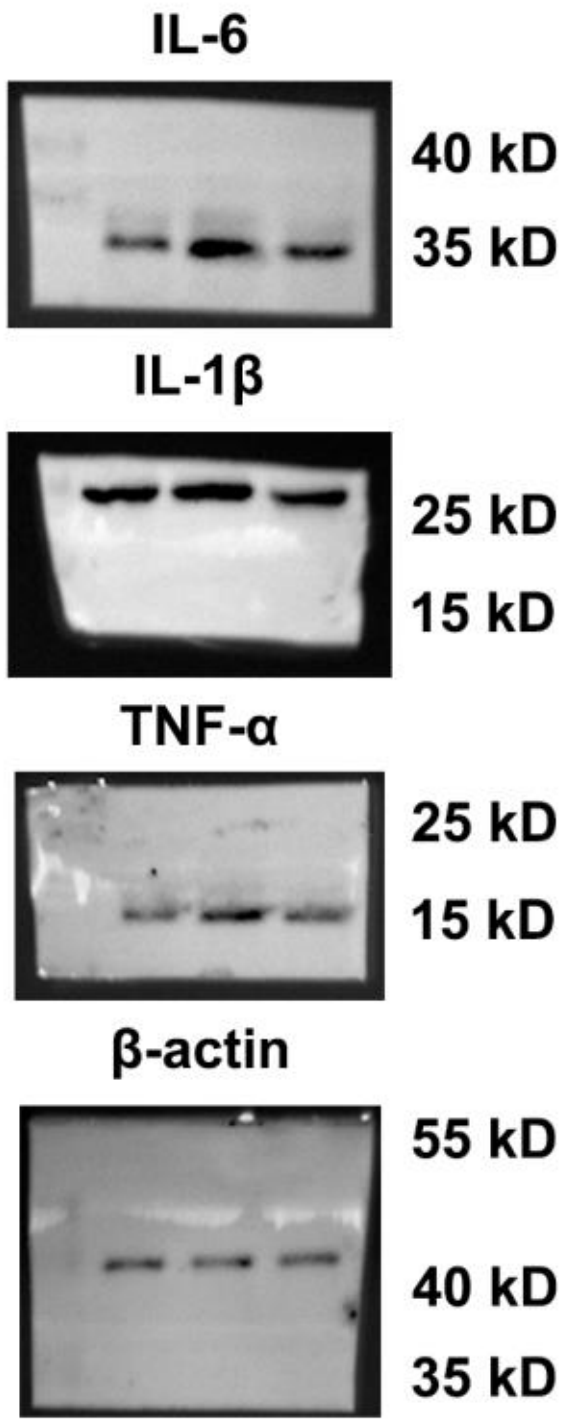

Figure 3A

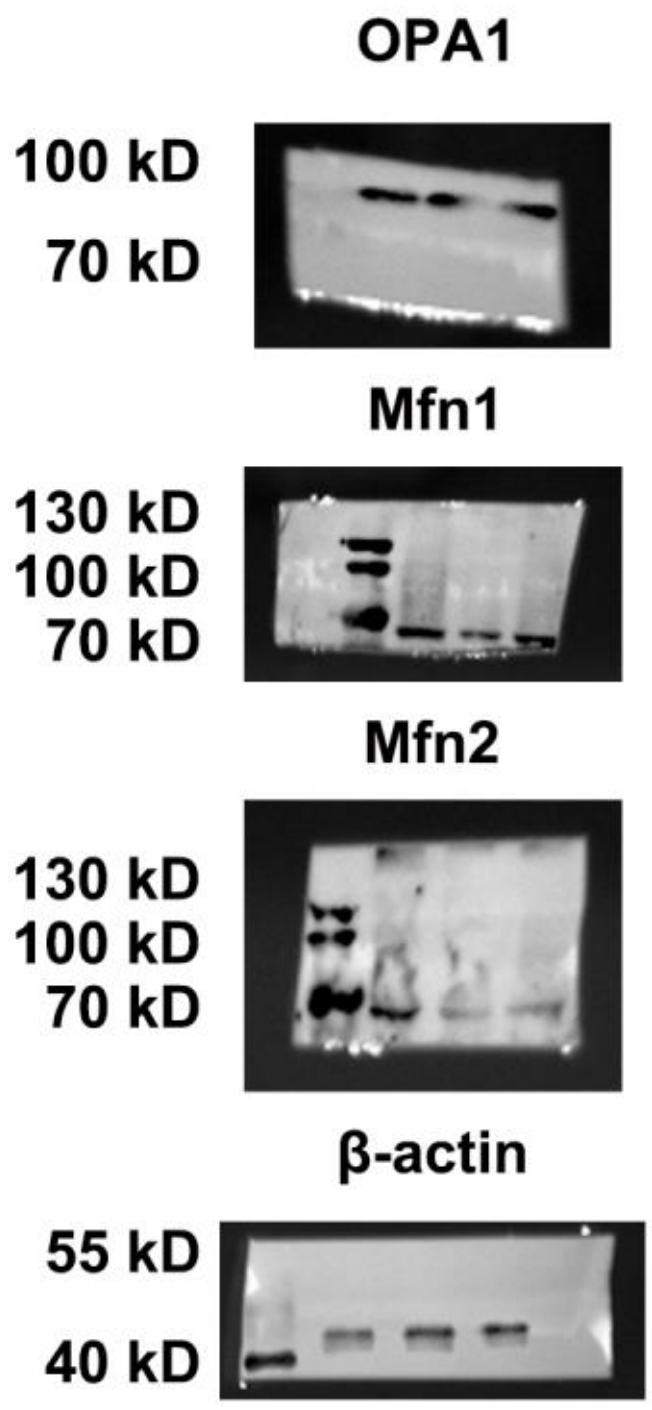

Figure 3E

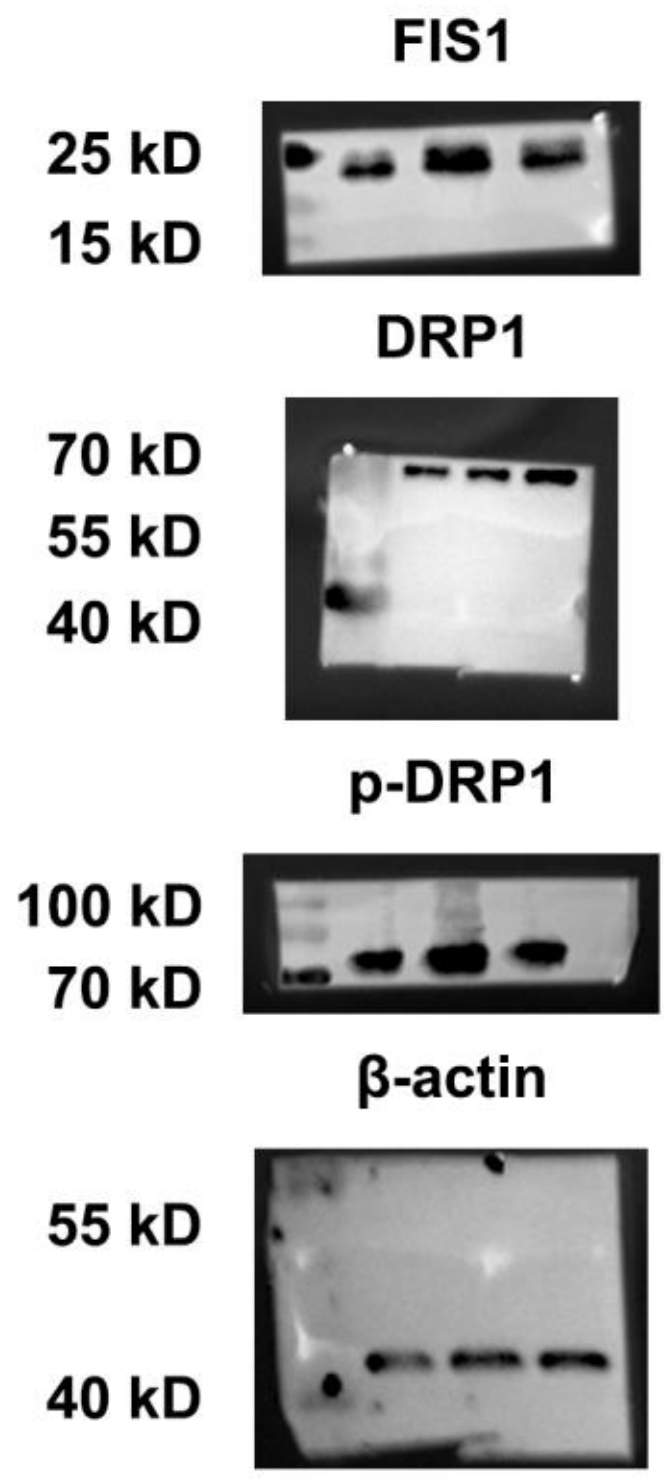

Figure 6A

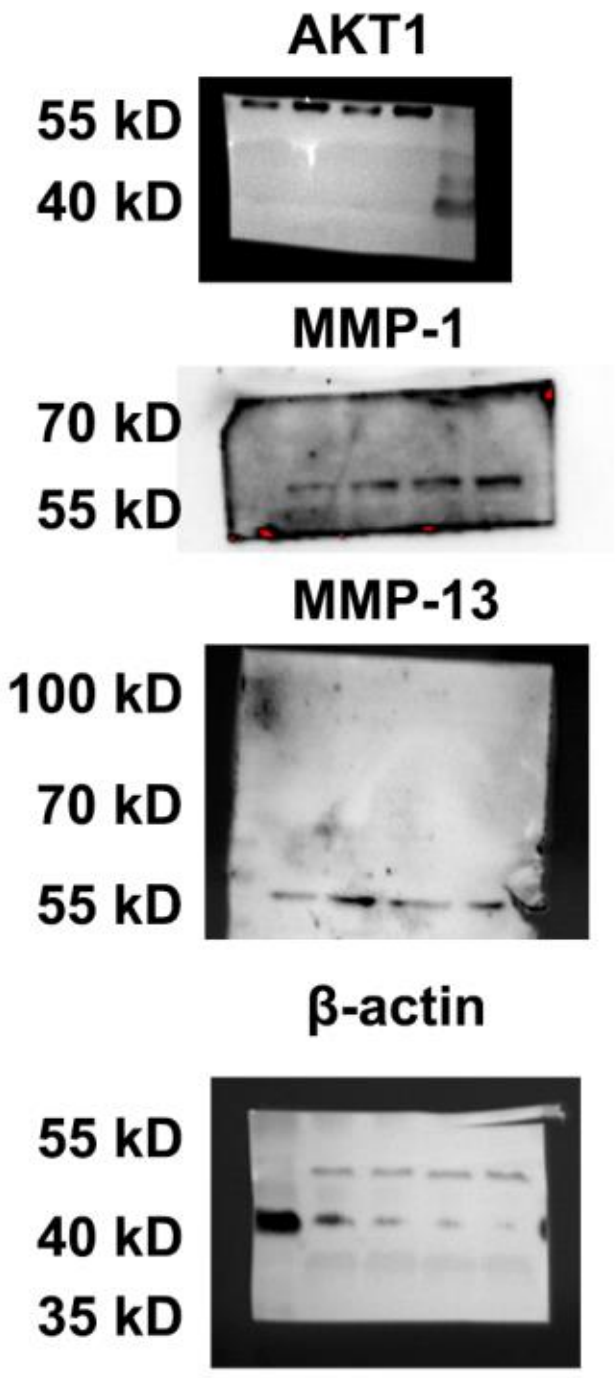

Figure 6D

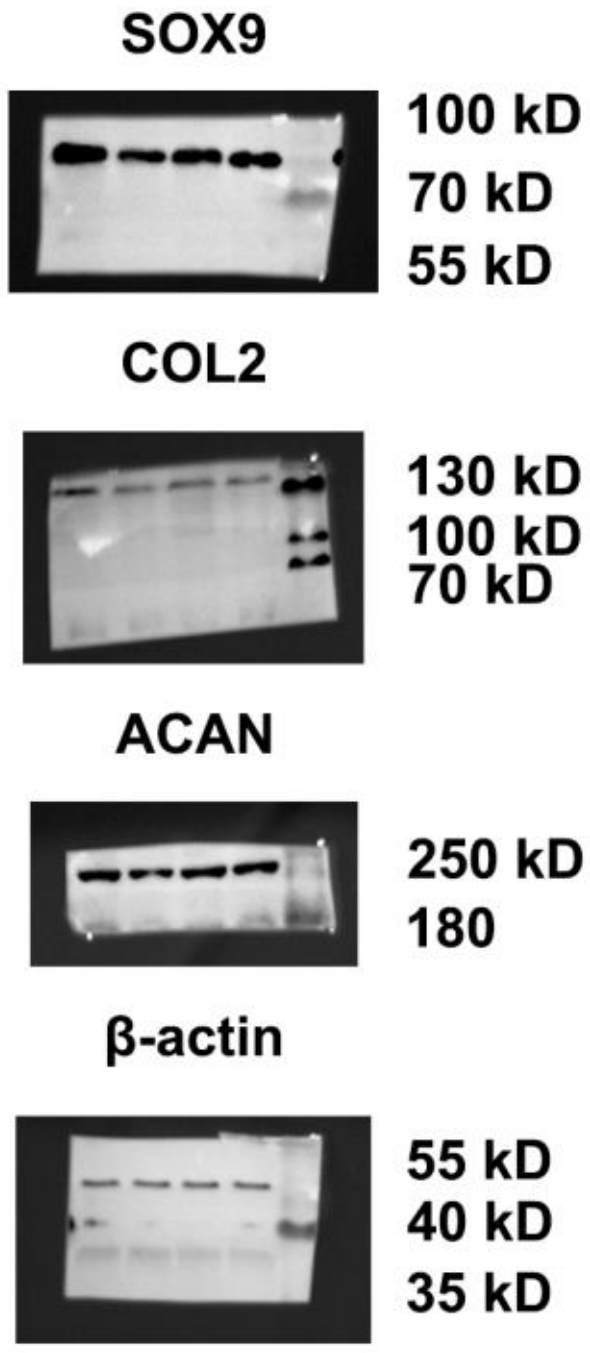

Figure 6H

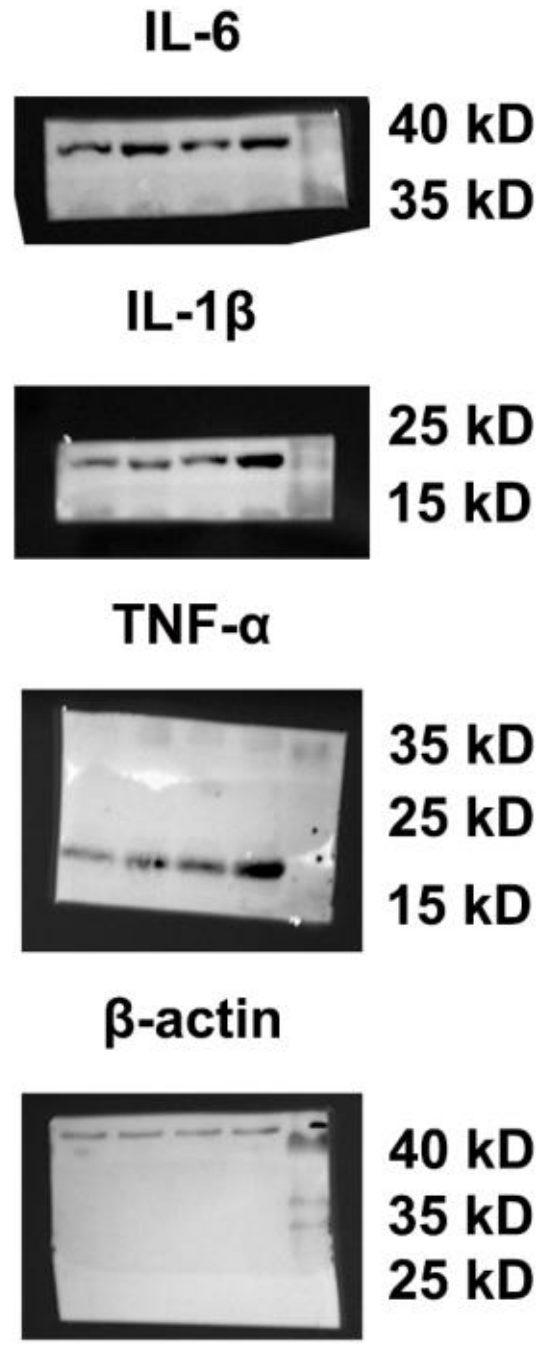

Figure 7A

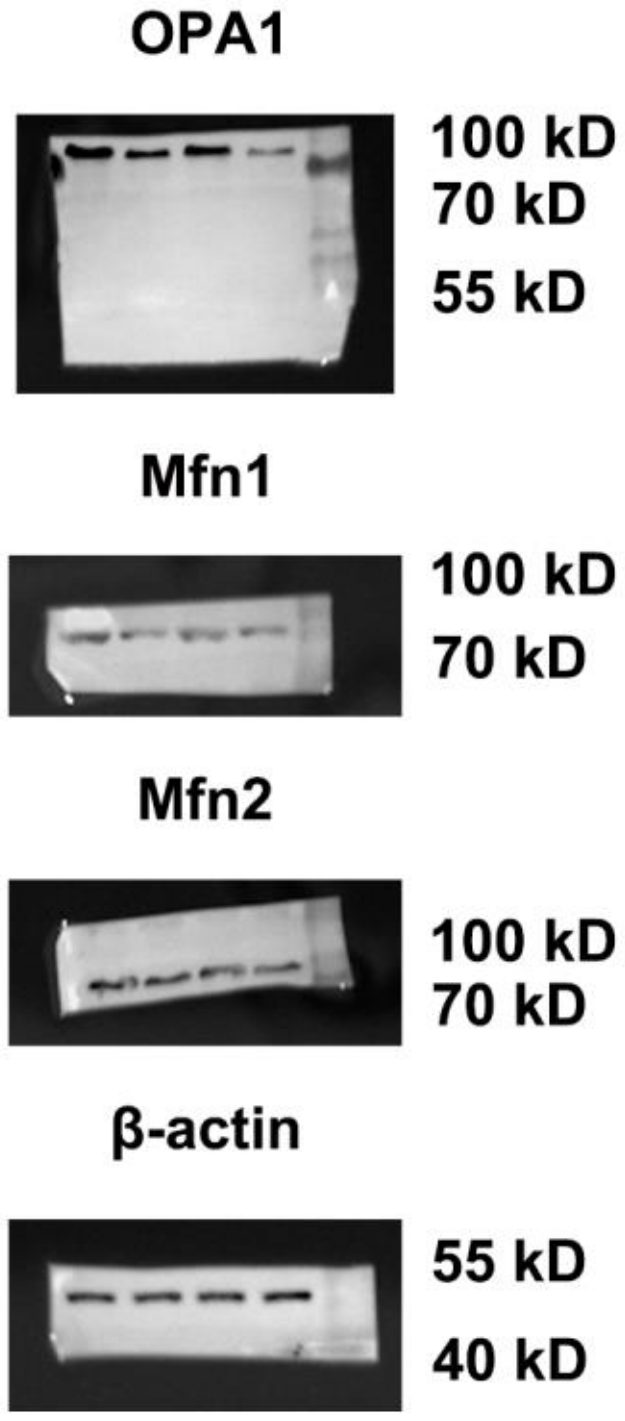

Figure 7E

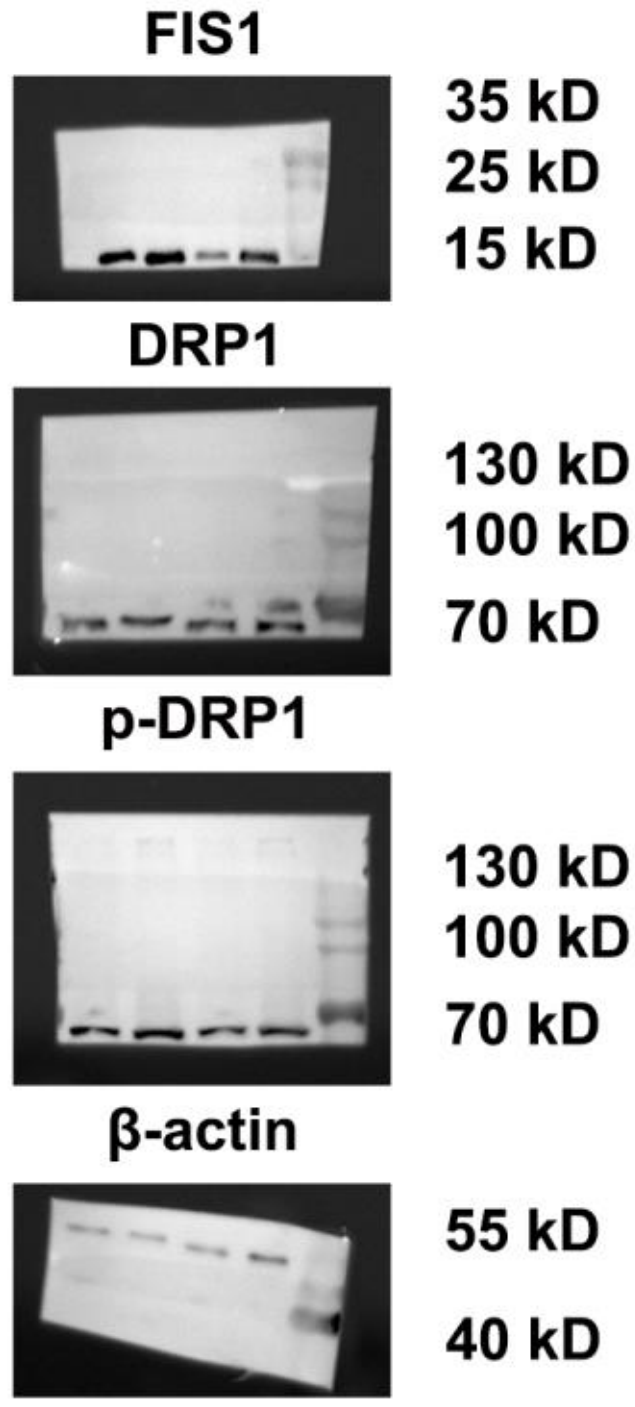

Figure 7A

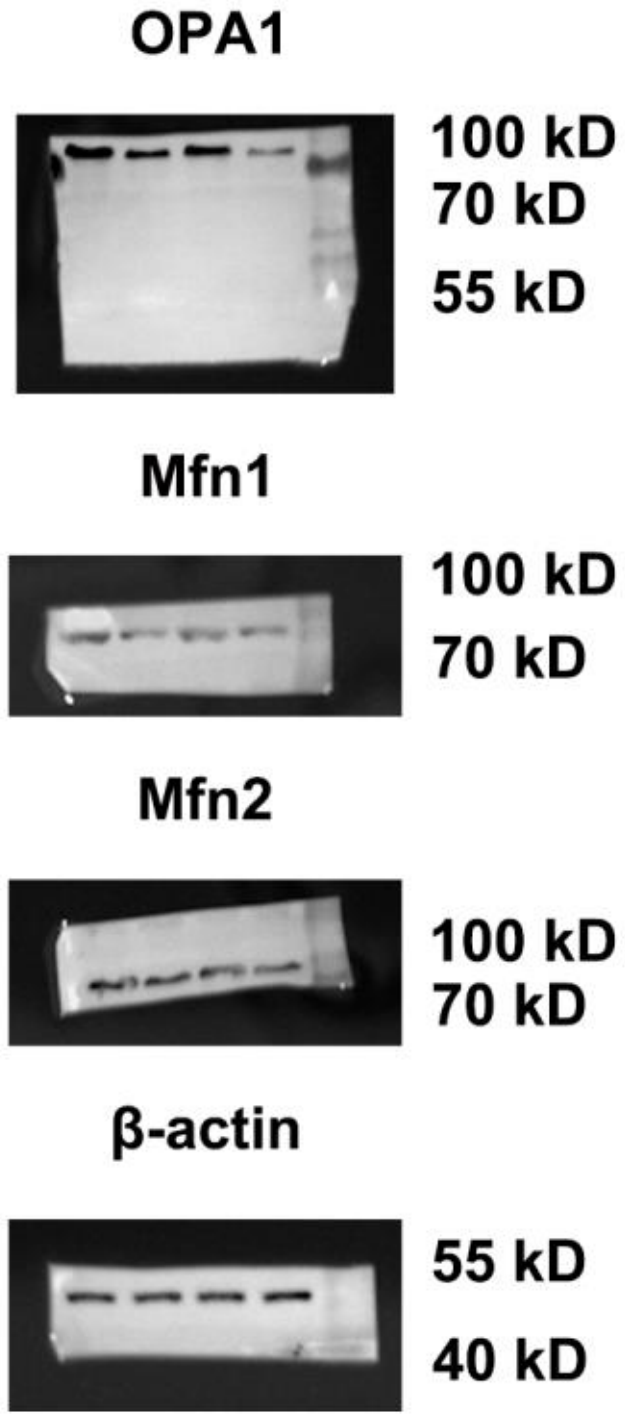

Figure 7E

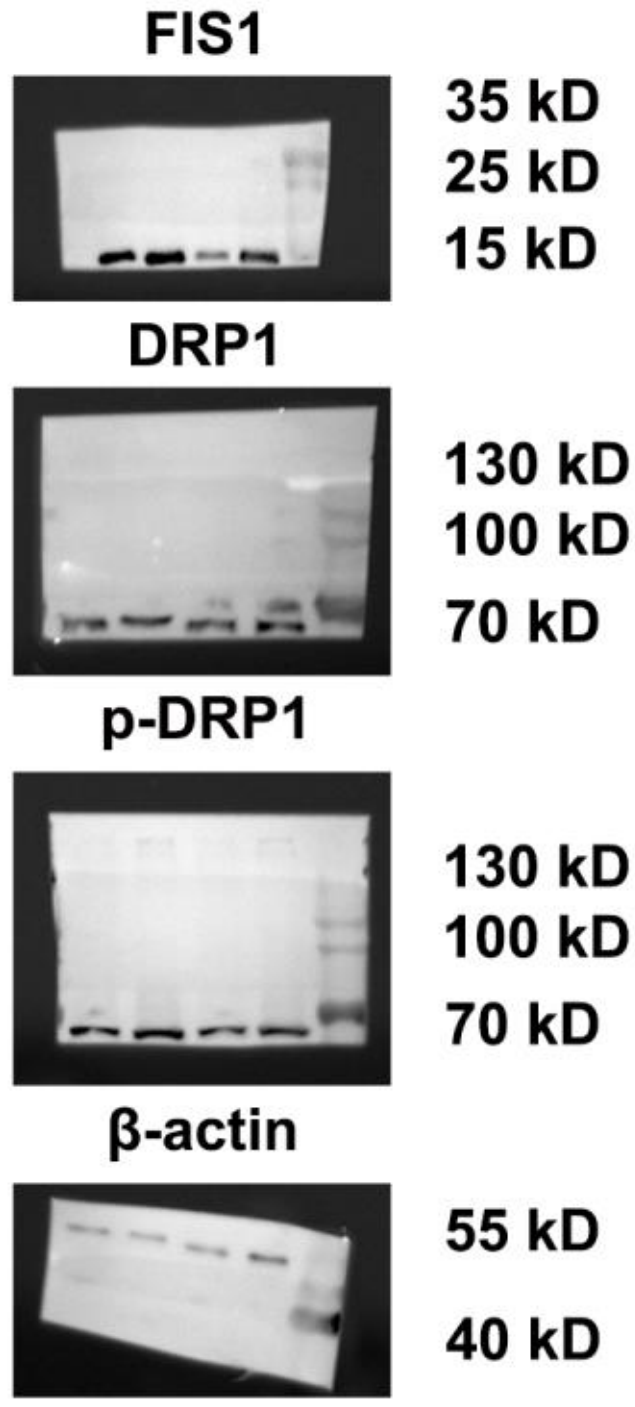

Figure S6A

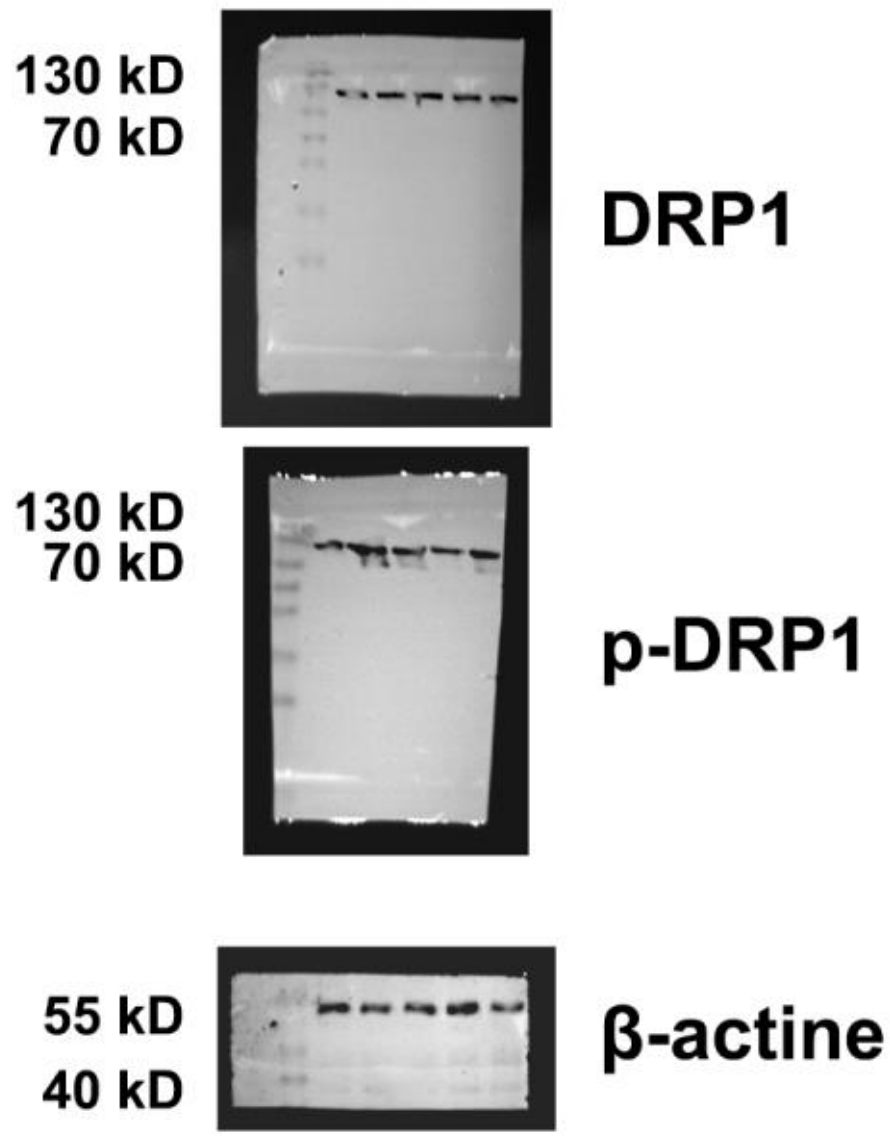

Supplement: Supplementary file 1 [file pone.0357989.s015.pdf]
