# Supplementary material for: Isoquercitrin ameliorates chondrocyte dysfunction in osteoarthritis by targeting AKT1-mediated mitochondrial dynamics regulation
Source: PLoS One. 2026 Sep 17;21(9):e0357989. doi: 10.1371/journal.pone.0357989 (PMC13585277; doi:10.1371/journal.pone.0357989)
Supplement: Supplementary file 3 [file pone.0357989.s018.docx]

**Point-by-Point Response to Reviewer Comments
Manuscript ID: PONE-D-26-14316
Title: Isoquercitrin ameliorates chondrocyte dysfunction in osteoarthritis by targeting AKT1-mediated mitochondrial dynamics regulation**

**Summary of Response:**

We sincerely thank Reviewers for their insightful comments and constructive suggestions. In this revision, we have addressed all concerns raised by Reviewers. Key updates to the manuscript are summarized as follows:

- Recalculate IC₅₀ of IQ (**Fig S1**).
- Perform IL‑1β-stimulated dose–response experiments (10/40/80 μM IQ) to detect p-DRP1/DRP1 expression (**Fig S6**).

Reviewer #1:

1. The dose of IQ (40 µM) is therefore still unvalidated in the presence of IL 1β (Fig 1); is clearly evident that 40 μM is a tolerated concentration, but No dose–response for IQ under IL-1β was ever performed. I think this is important.

**Response:** To address this issue completely, we supplemented a new CCK‑8 viability experiment where C28/I2 chondrocytes were co‑treated with fixed 10 ng/mL IL‑1β and serial concentrations of IQ (0, 5, 10, 20, 40, 80 μM) for 24 h (**Fig. S1C**). Consistent with our functional phenotypes, IL‑1β treatment markedly suppressed chondrocyte viability compared with the normal control group. IQ improved cell viability in a concentration‑dependent manner from 5 μM to 40 μM, reaching the maximal protective effect at 40 μM. When the concentration rose to 80 μM, the protective efficacy of IQ declined, accompanied by obvious cytotoxicity under inflammatory conditions.

These supplementary data robustly validate that 40 μM is the optimal therapeutic concentration for subsequent functional and mechanistic assays under IL‑1β stimulation.

Revised sentence added to Results: “*Second, we performed a targeted dose–response assay under standardized 10 ng/mL IL‑1β inflammatory stimulation (Figs S1C). IL‑1β alone drastically reduced chondrocyte viability. Co‑administration of IQ produced gradual, concentration‑dependent rescue of cell viability from 5 μM up to 40 μM, which yielded the maximum protective effect. However, the protective capacity declined sharply at 80 μM IQ, demonstrating obvious cytotoxicity in the inflammatory microenvironment. Collectively, the dual CCK‑8 datasets confirm that 40 μM IQ achieves maximal chondroprotection without observable toxicity under IL‑1β injury, justifying the use of this concentration for all subsequent functional experiments.”*

Revised sentence added to Materials and Methods:“*Second, dose–response detection under IL‑1β-induced inflammatory injury: chondrocytes were co-incubated with 10 ng/mL IL‑1β and serial IQ concentrations (0, 5, 10, 20, 40, 80 μM) for 24 h. Subsequently, 10 μL of CCK-8 reagent was supplemented to each well. After 2–4 h of incubation in the dark, optical density (OD) values were measured via a Multiskan FC microplate reader (Thermo Fisher Scientific, USA).*”

**Fig. S1.** **Cytotoxicity and protective effects of IQ on C28/I2 chondrocytes.** (A) CCK-8 assay showing the viability of C28/I2 chondrocytes treated with increasing concentrations of IQ (0–160 μM) for 24 h under normal culture conditions. (B) Nonlinear regression analysis determined the half-maximal inhibitory concentration (IC₅₀) of IQ in chondrocytes without inflammatory stimulation. (C) Dose–response curve of IQ in the presence of IL-1β-induced inflammatory injury. Chondrocytes were exposed to 10 ng/mL IL-1β together with serial concentrations of IQ (0, 5, 10, 20, 40, 80 μM) for 24 h. Data represent the mean ± SD of three independent biological replicates. Statistical analysis was performed by one-way ANOVA. **p < 0.01, ***p < 0.001, ****p < 0.0001.

**Location of the changes:** Fig. S1; Raw113-117; 289-297; 598-605.

2. “Results showed that IQ concentrations ≤ 80 μM had no adverse impact on chondrocyte viability, whereas 160 μM IQ induced slight cytotoxicity (Fig 1B).”

an IC₅₀ of 95.05 µM requires that viability at 160 µM be well below 50%; So "slight cytotoxicity" and "≤80 µM had no adverse impact" cannot both be true alongside the reported IC₅₀

**Response:** We apologize for the contradictory description inconsistent with the IC₅₀ value. After careful inspection, we identified inappropriate qualitative summaries in the original manuscript caused by coarse concentration gradients. The statement “IQ concentrations ≤ 80 μM had no adverse impact on chondrocyte viability, whereas 160 μM IQ induced slight cytotoxicity” conflicts with the IC₅₀ fitting result and has therefore been fully deleted. We optimized the IQ concentration layout and re-performed four-parameter logistic regression analysis based on updated viability data. The revised IC₅₀ is 91.76 μM (**Figs. S1B, C**). In the revised Results section, we only objectively describe the trend that cell viability decreases gradually as IQ concentration increases, avoiding inaccurate subjective judgments.

Revised sentence added to Results: “*Prior to functional assays, two independent CCK‑8 viability tests were conducted to identify the optimal IQ dosage. First, broad-range gradient treatment without IL‑1β was used to evaluate intrinsic IQ cytotoxicity. Four‑parameter nonlinear regression fitted an IC₅₀ of 91.76 μM for resting chondrocytes; mild growth inhibition emerged above 80 μM, while concentrations ≤40 μM exerted negligible toxic effects on normal chondrocytes (Fig 1B and Figs S1A, B).”*

Revised sentence added to Materials and Methods:“*Two parallel CCK‑8 assays were performed separately: First, cytotoxic screening of IQ without IL‑1β stimulation: cells were exposed to IQ at concentrations of 0, 20, 40, 60, 80, 100, 120, 140, 160 μM for another 24 h to calculate the IC₅₀ value of IQ under normal culture conditions.*”

**Fig. S1.** **Cytotoxicity and protective effects of IQ on C28/I2 chondrocytes.** (A) CCK-8 assay showing the viability of C28/I2 chondrocytes treated with increasing concentrations of IQ (0–160 μM) for 24 h under normal culture conditions. (B) Nonlinear regression analysis determined the half-maximal inhibitory concentration (IC₅₀) of IQ in chondrocytes without inflammatory stimulation. (C) Dose–response curve of IQ in the presence of IL-1β-induced inflammatory injury. Chondrocytes were exposed to 10 ng/mL IL-1β together with serial concentrations of IQ (0, 5, 10, 20, 40, 80 μM) for 24 h. Data represent the mean ± SD of three independent biological replicates. Statistical analysis was performed by one-way ANOVA. **p < 0.01, ***p < 0.001, ****p < 0.0001.

**Location of the changes:** Fig. S1; Raw110-112; 285-289; 598-605.

3. The wavelength (490 nm) appears only in the rebuttal, not in the manuscript (Rows 109–112 give the instrument only).

**Response:** We appreciate the reviewer’s meticulous reading. As pointed out, the absorbance wavelength (490 nm) for the CCK‑8 measurement was only mentioned in our response letter and omitted within the main text. To fix this omission, we have added the wavelength parameter to the CCK‑8 experimental description in the Materials and methods section.

Revised sentence added to Materials and Methods: “*After 2-4 h of incubation in the dark, optical density (OD) values were measured at 490 nm via a Multiskan FC microplate reader (Thermo Fisher Scientific, USA).*”

**Location of the changes:** Raw108-109.

4. “Explain the ImageJ protocol for blot quantification”, you did not added in the manuscript a clear protocol The grayscale / background-subtraction / integrated-density description exists only in the rebuttal

**Response:** We appreciate this meticulous comment. The detailed ImageJ blot quantification workflow was only mentioned in our previous rebuttal and absent from the original manuscript. We have supplemented the full quantitative operation steps in the Western blotting subsection of Materials and Methods. The added content clarifies grayscale conversion, unified background subtraction and integrated density normalization against β‑actin internal control, and all revisions are highlighted in the tracked-changes manuscript for easy checking.

Revised sentence added to Materials and Methods: “*Unmodified original blot images were quantified with ImageJ software (National Institutes of Health, Bethesda, MD, USA; https://imagej.net/ij/) [34]: images were converted to grayscale, background was subtracted uniformly, and relative protein expression was calculated as the integrated density of target band divided by that of β‑actin (internal control).*”

**Location of the changes:** Raw155-159.

5. Statistical analysis: defines significance only as *P<0.05 and **P<0.01, while every figure legend uses *** and ****.

**Response:** We sincerely thank the reviewer for this rigorous inspection of our statistical criteria consistency. We have updated the Statistical analysis section to fully supplement the definitions of ***P < 0.001, ****P < 0.0001; #P < 0.05, ##P < 0.01, ###P < 0.001, ####P < 0.0001, matching all labeling standards in each figure. All revisions are marked in the tracked-changes manuscript.

Revised sentence added to Materials and Methods: “*One-way analysis of variance (ANOVA) combined with Tukey’s post hoc test was applied for multiple group comparisons using GraphPad Prism 9.5.0 for Windows (GraphPad Software, Inc., San Diego, CA, USA; https://www.graphpad.com); significance was defined as *P < 0.05, **P < 0.01, ***P < 0.001, ****P < 0.0001 and #P < 0.05, ##P < 0.01, ###P < 0.001, ####P < 0.0001.*”

**Location of the changes:** Raw 274-275.

6. I was wondering, why there is not IQ-alone group (IQ without IL‑1β) in any functional assay, so IQ's baseline effect on ECM, cytokines and mitochondrial proteins is unknown. an IQ-only arm would also strengthen the safety claim you make in the opening Results paragraph.

**Response:** We sincerely appreciate this valuable comment raised by the reviewer. We fully recognize the deficiency that no IQ-only treatment group (without IL‑1β stimulation) was included in our functional detection system, which makes the baseline biological effects of IQ on ECM anabolic proteins, pro-inflammatory cytokines and mitochondrial dynamic proteins unclear under physiological conditions; this omission also weakens the biosafety statement of IQ described at the beginning of the Results section, and we acknowledge this as an inadequate experimental design in our original manuscript.

For our initial experimental layout, we referenced mainstream chondrocyte OA studies from published flavonoid-related literature (refs., *J. Nutr. Biochem.* **2026**, *153, 110306*; *Molecules* **2022**, *27, 3459; Aging* **2023***, 15:4861-4874*). The core scientific focus of this study is to clarify the rescue effect and underlying mechanism of IQ against IL‑1β-evoked chondrocyte dysfunction. Therefore, almost all treatment groups were established on the basis of the IL‑1β-induced chondrocyte injury model, with the normal untreated group as the only blank control.

Unfortunately, due to limited remaining materials and tight experimental schedule, we are unable to supplement full sets of WB, qPCR and staining assays with an IQ-only group covering all functional indicators in this revision. To fix this flaw reasonably, we have made two key revisions to the manuscript:

1. We completely deleted the overemphasized descriptive sentences regarding the broad safety profile of IQ in the opening paragraph of Results, removing the unsupported biosafety claim that lacks corresponding IQ-only experimental evidence;
2. We supplemented a dedicated limitation subsection in the Discussion chapter, explicitly stating the absence of the IQ-only group as a present experimental shortcoming, and proposed relevant supplementary detection (ECM, inflammatory and mitochondrial markers under IQ single treatment) as a clear direction for our follow-up in-depth research.

Revised sentence added to Results: “*One key limitation of the current work is the absence of an IQ single-treatment experimental group to independently dissect its standalone biological activity; subsequent follow-up work will supplement detections of ECM, inflammatory and mitochondrial markers under IQ monotherapy to fully clarify its intrinsic regulatory effects.*”

**Location of the changes:** Raw 587-591.

7. The opening Results paragraph on ADMET/ecotoxicity ("low toxicity to aquatic and terrestrial organisms, no bioaccumulation") is uncited, is not a result of this study

**Response:** We fully agree with the reviewer’s valuable suggestion. The original descriptions about ADMET characteristics, aquatic and terrestrial ecotoxicity as well as bioaccumulation are irrelevant background information not generated from our present chondrocyte experiments, and the lack of citations also causes non-compliance with manuscript referencing norms. Therefore, we have completely deleted this paragraph of extraneous content.

8. “Other common chemical reagents including PFA and SKIM MILK were purchased from domestic qualified suppliers.” Still in the manuscript, but in the rebuttal <>

**Response:** We appreciate the reviewer’s careful reminder. We have fully removed this redundant sentence regarding common chemical reagents from the revised manuscript.

9. I suggest a 2–3 point dose–response (e.g. 10 / 40 / 80 μM) for p-DRP1/DRP1 for IQ under IL-1β (a single western blot gel)

**Response:** We appreciate the reviewer’s recommendation to test a 3-point dose response of IQ on p-DRP1/DRP1 under IL‑1β. Given the nearly identical molecular weight of p-DRP1 and DRP1, simultaneous detection on the same membrane is not feasible. We thus used sequential stripping and re-probing on one single gel containing all experimental groups.

The newly acquired western blot results consistently verify that IL‑1β significantly increases the p-DRP1 level and elevates the p-DRP1/DRP1 ratio (**Fig. S6**). IQ treatment reverses DRP1 phosphorylation in a dose-dependent manner: 40 μM IQ exhibits the strongest inhibitory effect, while the protective effect is partially attenuated at 80 μM IQ. These immunoblot data are highly consistent with our CCK-8 cell viability results, jointly confirming that 40 μM IQ represents the optimal working concentration to suppress excessive DRP1 phosphorylation and mitochondrial fission.

Revised sentence added to Results: “*To further clarify the optimal working concentration of IQ in regulating mitochondrial fission, we established a 3-point dose-response model under IL‑1β stimulation* *(Fig. S6). Treatment with IQ dose-dependently suppressed IL‑1β-triggered DRP1 activation. The maximal inhibitory effect was observed at 40 μM IQ, whereas the protective effect was partially weakened at 80 μM IQ. Consistent with our CCK‑8 cell viability data, these results confirm that 40 μM IQ is the optimal concentration to restrain excessive DRP1 phosphorylation.*”

**Fig. S6. IQ inhibits IL‑1β‑induced DRP1 phosphorylation in a dose-dependent manner.** (A) Western blot bands of p-DRP1 and total DRP1. (B, C) Quantitative analysis of relative p-DRP1 expression and p-DRP1/DRP1 ratio. All samples were loaded on a single gel; sequential stripping and re-probing of the same membrane was performed owing to similar molecular weights of p-DRP1 and DRP1. β‑actin served as internal control. Data are mean ± SD (n = 3). ****p < 0.0001 vs. Con; ##p < 0.01, ###p < 0.001, ####p < 0.0001 vs. IL‑1β+IQ (40 μM) group. One-way ANOVA with Tukey’s test.

**Fig. S17**. Original Western blot images for Fig S3.

**Location of the changes:** FigS6; S17; Raw 366-372; 626-632; 668.

10. Fig 7 E, DRP1 in those with IQ is visible a doublet.

Please state which band was used for total DRP1 quantification, and whether both were included. If the relative intensity of the two bands changes across conditions, this should be commented on.

**Response:** We thank the reviewer for this critical observation. For total DRP1 quantification in Fig.7E, we summed the integrated density of both the upper and lower bands of the DRP1 doublet using a unified ROI in ImageJ. This inter-group band variation has been fully counted in our total DRP signal without introducing analytical deviation. A corresponding description of our doublet quantification method has been added to the Figure 7 legend, with all revisions highlighted in the tracked manuscript.

Revised sentence added to the Figure 7 legend: “*Note: Total DRP1 appeared as a doublet band on immunoblots. Both upper and lower bands were enclosed in one ROI and their total integrated density was summed for quantification.*”

**Location of the changes:** Raw 533-535.

11. Pay attention for the claims that exist in rebuttal, but are absent from manuscript; double check all.

**Response:** We thank the reviewer for this important reminder. We have thoroughly cross-referenced all contents in the prior rebuttal against the full manuscript. All key experimental parameters, quantification protocols, statistical rules and supplementary experimental schemes that were only written in the rebuttal have been supplemented into the revised main text. Irrelevant redundant paragraphs have been completely removed.
