# Supplementary material for: Isoquercitrin ameliorates chondrocyte dysfunction in osteoarthritis by targeting AKT1-mediated mitochondrial dynamics regulation
Source: PLoS One. 2026 Sep 17;21(9):e0357989. doi: 10.1371/journal.pone.0357989 (PMC13585277; doi:10.1371/journal.pone.0357989)
Supplement: Supplementary file 4 [file pone.0357989.s017.docx]

**Point-by-Point Response to Reviewer Comments
Manuscript ID: PONE-D-26-14316
Title: Isoquercitrin ameliorates chondrocyte dysfunction in osteoarthritis by targeting AKT1-mediated mitochondrial dynamics regulation**

**Summary of Response:**

We sincerely thank Reviewers for their insightful comments and constructive suggestions. In this revision, we have addressed all concerns raised by Reviewers. Key updates to the manuscript are summarized as follows:

- Calculate the IC50 value of IQ (**Fig S1**).
- Add more experimental evidence of AKT1 overexpression (**Figs 6A, 6B** and **Fig S7A**).
- Perform co-localization fluorescence imaging to verify that IQ alleviates IL-1β-triggered mitochondrial oxidative DNA damage in chondrocytes in an AKT1-dependent manner (Fig S12).
- Supply all original raw western blot images for this work (**Figs S12–S15**).

Reviewer #1: Overall, this is a well-structured study with a clear logical flow from bioinformatic prediction to experimental validation. The combination of molecular dynamics and rescue experiments provides a solid mechanistic foundation. I found this a nice work. However: it can be improved, especially in the material and methods section, here my suggestions. please recheck for misspelled words, in particular in the graphs and pictures.

**General response**: We thank Reviewer #1 for his/her positive evaluation of our work on isoquercitrin ameliorating chondrocyte dysfunction in osteoarthritis by targeting AKT1-mediated mitochondrial dynamics, as well as the highly constructive and meticulous comments that have greatly helped us improve the manuscript. In response to the constructive comments, we have revised the manuscript thoroughly. We carefully checked and corrected all spelling errors throughout the manuscript, particularly in figures and legends. In addition, we have further refined and supplemented the Materials and Methods section, adding detailed descriptions of experimental procedures and parameters as requested. Below, we provide our detailed point-by-point responses to all comments.

Several critical parameters are missing from the Materials and Methods section

• EdU Staining for Cell Proliferation, line 80: missing details for “click reaction” and for about the microscope.

**Response:** We thank the reviewer for pointing out these missing details in the Materials and Methods section. We have supplemented the detailed experimental procedures of EdU staining in the revised manuscript. Specifically, we added a complete description of the click reaction and relevant parameters of the fluorescence microscope. In addition, we have also supplemented the full information of the analysis software including version number and official website in accordance with the journal requirements. The updated text reads as follows:“*Following an additional PBS wash, the click reaction was performed using the kit-provided reaction mixture according to the manufacturer’s instructions for 30 min at room temperature in the dark. Nuclei were counterstained with DAPI for 10 min in the dark. Fluorescence images were captured using an inverted fluorescence microscope (DMIL LED, Leica Microsystems, Wetzlar, Germany) equipped with a 10× objective lens. The number of EdU-positive proliferative cells were counted using ImageJ software (National Institutes of Health, Bethesda, MD, USA; https://imagej.net/ij/) [34].*”

**Location of the changes:** Row 118-124.

• Western Blotting: % of gels? Were them gradient or not? equivalent protein …how much? Please describe the” proper conditions” also; blocked with 5% (w/v) skim milk and in what did you dissolved the milk powder? Please add details You listed primary antibodies but omitted the dilutions for secondary antibodies.

**Response:** We apologize for the lack of detailed experimental parameters in the original manuscript, which compromised the rigor of our work. We have now fully revised and supplemented the Western blotting section as requested, with detailed additions listed as follows:

1. We clearly stated that 6% or 12% non-gradient SDS-PAGE gels were used for protein separation, and the loading quantity of each protein sample was set at 30 μg.
2. We used 5% (w/v) bovine serum albumin (BSA) diluted in TBST for membrane blocking. BSA was chosen because skim milk contains endogenous immunoglobulins, which may cause non-specific binding and interfere with the detection of target proteins.
3. We supplemented the dilution ratio (1:5000) for HRP-conjugated secondary antibodies.
4. We added complete information of the image analysis software ImageJ, including its developer, official website and corresponding reference, in accordance with journal requirements.

All optimized experimental details have been incorporated into the revised manuscript to guarantee experimental repeatability.

Revised sentence added to Western Blotting:“*Total protein was isolated using radio-immunoprecipitation assay (RIPA) lysis buffer containing protease and phosphatase inhibitor cocktails. C28/I2 chondrocytes were collected, rinsed with cold PBS, lysed on ice, and centrifuged to harvest supernatants. Protein concentration was quantified via the bicinchoninic acid (BCA) assay. Equal amounts of 30 µg protein per sample were separated on two distinct SDS-PAGE gels according to target molecular weights: 6% constant gels for high-molecular-weight proteoglycans (e.g., aggrecan (ACAN)), and 12% constant gels for low-molecular-weight proteins. After transfer to polyvinylidene fluoride (PVDF) membranes, membranes were blocked with 5% (w/v) bovine serum albumin (BSA) dissolved in TBST for 1 hour at room temperature, rinsed with TBST, and incubated overnight at 4 °C with primary antibodies (β-actin: 66009-1-Ig, 1:10000, Proteintech; matrix metallopeptidase 1 (MMP-1): HY-P80754, 1:1000, MedChemExpress; matrix metallopeptidase 13 (MMP-13): YA3143, 1:1000, MedChemExpress; SRY-box transcription factor 9 (SOX9): HY-P80355, 1:500, MedChemExpress; type II collagen (COL2): HY-P81047, 1:2000, MedChemExpress; aggrecan (ACAN): ab3778, 1:1000, Abcam; interleukin 6 (IL-6): ab233706, 1:1000, Abcam; interleukin 1 beta (IL-1β): HY-P80503, 1:1000, MedChemExpress; tumor necrosis factor alpha (TNF-α): HY-P80914, 1:800, MedChemExpress; optic atrophy 1 (OPA1): ab42364, 1:1000, Abcam; mitofusin 1 (MFN1): ab221661, 1:1000, Abcam; mitofusin 2 (MFN2): ab56889, 1:1000, Abcam; mitochondrial fission 1 protein (FIS1): ab156865, 1:10000, Abcam; dynamin-related protein 1 (DRP1): 12957-1-AP, 1:5000, Proteintech; DRP1 (phospho S616): ab314755, 1:1000, Abcam). Then, membranes were incubated with horseradish peroxidase (HRP)-conjugated anti-rabbit or anti-mouse secondary antibodies (1:5000) for 1 h at room temperature. Finally, the membranes were observed by an enhanced chemiluminescence (ECL) kit (Millipore, Billerica, MA, USA) and quantified by ImageJ software (National Institutes of Health, Bethesda, MD, USA; https://imagej.net/ij/) [34]. β-actin was used as an internal control.*”

**Location of the changes:** Row 126-150.

• JC-1 Staining: please add details

**Response:** We have now supplemented all critical experimental parameters in the revised manuscript, including the kit information, working solution preparation, incubation conditions, washing steps, fluorescence detection settings, and imaging system specifications, as shown below:

“*The working solution was prepared by diluting the JC-1 stock (200×) with ultrapure water and JC-1 staining buffer (5×) according to the standard protocol. Post-treatment, cells were rinsed with pre-warmed PBS, incubated with JC-1 working solution at 37 °C for 20 min in the dark, then washed twice with ice-cold JC-1 staining buffer (1×). Fluorescence signals were visualized using an inverted fluorescence microscope (Leica DMIL LED, Leica Microsystems, Wetzlar, Germany), with red fluorescence (JC-1 aggregates) and green fluorescence (JC-1 monomers) detected at excitation/emission wavelengths of 525/590 nm and 490/530 nm, respectively.*”

**Location of the changes:** Row 166-173

• Line 162: “cells were digested, resuspended, and seeded” would you say “detached”?

just to be clear: "Digested" usually refers to the process of breaking down tissue to get cells. For moving cells from one plate to another, "detached" or "trypsinized" is the standard terminology.

**Response:** Thank you for this helpful comment on terminology clarity. We agree that “digested” is more commonly used for dissociating cells from tissues, whereas “detached” or “trypsinized” is the standard term for releasing adherent cells from culture plates. We have revised the manuscript accordingly, replacing the phrase “cells were digested, resuspended, and seeded” with “cells were detached with trypsin-EDTA, resuspended, and seeded”. This correction improves the precision and clarity of the experimental description.

**Location of the changes:** Row 251, 252.

• Fig 1C: Cell viability assay of C28/I2 chondrocytes treated with gradient IQ concentrations (0-160 μM) under IL-1β stimulation (or without??), please add in the caption the concentration for IL-1β, please confirm that you obtained 100% viability after exposing chondrocyte to IL-1β. How did you decide the 10ng/ml IL-1β amount?

I think there is a confusion in data representation: put all the graphs surviaval for isoquercitine only, surviaval for IQ + IL-1β, surviaval for IL-1β only. IL-1β: error bar is a bit big, how many replicates did you do? They appears like 2 distinct groups.

**Response:** We greatly appreciate the reviewer’s valuable comments and suggestions. We have addressed all points one by one in detail as follows:

1. **Supplementation of IL-1β concentration and selection rationale**

We have added relevant descriptions in the Results section. C28/I2 chondrocytes were treated with 10 ng/mL IL-1β to establish an OA-mimetic in vitro model. This concentration is widely validated and commonly adopted in published related studies (refs., *J Extracell Vesicles*. **2021**, *10*, e12160; *Nat Commun*. **2020**, *11*, 3427), so we directly applied it without additional dose optimization. Revised sentence added to Results:“*To explore the influence of IQ on the proliferation of OA-like chondrocytes, C28/I2 cells were stimulated with 10 ng/mL IL-1β (a standard concentration used in prior studies) to construct an in vitro model [35,36].*

2. **Explanation for cell viability assay design and data classification**

Prior to functional experiments, we performed CCK-8 assays to screen the working concentration of IQ. This assay was conducted in chondrocytes without IL-1β stimulation. We further calculated the IC₅₀ of IQ using a four-parameter logistic model, and the value was 95.05 μM (95% CI: 77.63–629.2 μM), as shown in **Fig S1**. Since 40 μM was well below the cytotoxic concentration, we selected 40 μM IQ for all subsequent experiments. We did not perform separate CCK-8 tests for the IL-1β-only group and IQ + IL-1β group. The 10 ng/mL IL-1β adopted here is a mature and well-recognized concentration in chondrocyte research, and its influence on cell viability has been fully verified in previous work. We apologize that we did not optimize IQ concentration under IL-1β treatment via cell viability assays. However, subsequent functional experiments fully demonstrated that 40 μM IQ exerted favorable protective effects against IL-1β-induced chondrocyte damage. Given that 40 μM IQ was used uniformly across the entire study, re-running full concentration-gradient tests under IL-1β stimulation would require a substantial amount of extra work. We hereby promise to explore the optimal IQ concentration in the presence of IL-1β in our future investigations. Revised sentence added to Results:“*Prior to functional assays, we assessed the cytotoxicity of IQ in C28/I2 chondrocytes without 10 ng/mL IL-1β stimulation using the CCK-8 assay, so as to determine the optimal working concentration for subsequent experiments.*

*We further calculated the half-maximal inhibitory concentration (IC₅₀) of IQ using a four-parameter logistic model, and the value was 95.05 μM (95% CI: 77.63–629.2 μM), as presented in Fig S1. Given that 40 μM was well below the cytotoxic IC₅₀, this concentration was selected for all subsequent experiments.*”

Revised the legend for **Fig 1B**: “*Cell viability of C28/I2 chondrocytes treated with gradient concentrations of IQ without IL-1β stimulation. **p < 0.01, ****p < 0.0001 vs. 0 μM group.”*

3. **Revision of Fig 1C and explanation for error bars and replicates**

We sincerely apologize for the large error bars and discrete data distribution in the original **Fig 1C**. This issue was caused by inappropriate statistical processing of the original datasets, rather than insufficient biological replicates. We have re-analyzed data from independent biological replicates with correct statistical methods and updated **Fig 1C** accordingly. The revised graph shows obviously reduced variability, and the statistical significance of IQ’s protective effect remains robust. The exact number of replicates has also been clearly marked in the updated figure legend.

**Fig. S1.** Cell viability of *C28/I2* chondrocytes treated with gradient concentrations of IQ (0–160 μM). The dose-response curve was fitted using a four-parameter logistic model, and the IC₅₀ value was determined to be 95.05 μM (95% CI: 77.63–629.2 μM).

**Fig 1. Preliminary chondroprotective evaluation of IQ. A.** DAPI/EDU co-staining of C28/I2 chondrocytes. DAPI (blue) stains cell nuclei, and EDU (green) labels proliferating cells (scale bar: 200 μm). **B.** Cell viability of C28/I2 chondrocytes treated with gradient concentrations of IQ without IL-1β stimulation. **p < 0.01, ****p < 0.0001 vs. 0 μM group. **C.** Quantification of EdU-positive cells. Data are presented as mean ± standard deviation (SD). **p < 0.01, ****p < 0.0001 vs. Con; ###p < 0.001 vs. IL-1β group. One-way ANOVA with Tukey’s post hoc test was used for multiple comparisons.

**Location of the changes:** Row 273-276; 280-282; 284-287; 295-298; Fig 1C; Fig. S1.

• Please indicate clearly in the main manuscript that qPCR primers are listed in the supporting material as well as that you normalized per GAPDH.

**Response:** We sincerely apologize for this oversight in the original submission. We have now clearly stated in the main manuscript that all qPCR gene expression data were normalized to the housekeeping gene GAPDH, and the complete sequences of all primers used in this study are explicitly listed in Table 1 in the Supporting Information. These additions ensure full transparency and reproducibility of the qPCR experiments, as requested.

Revised sentence added to Materials and Methods:“*The relative gene expression was calculated by the 2^(-ΔΔCt) method and normalized to GAPDH. The primer sequences used for qRT-PCR are provided in Table 1.*”

**Location of the changes:** Row 158-161.

• Fig 2 e fig 3 e fig 6: β-action…. you would say β-actine?

**Response:** Thank you very much for pointing out this typographical error. We sincerely apologize for the confusion caused by the misspelling of “β-actin” in the figure labels. We have now carefully reviewed and corrected all instances of “β-action” to the correct term “β-actin” in Figs 2, 3, 6 and 7 as well as in the corresponding figure legends and main text. All corrections are clearly marked in the revised manuscript.

**Location of the changes:** Figs 2, 3, 6 and 7.

• Graphical abstract: dOgradation.

**Response:** We sincerely apologize for this spelling error, which was caused by our carelessness during the final check. We greatly appreciate the reviewer’s careful observation and helpful feedback. We have corrected “dOgradation” to the correct spelling “degradation” in the revised graphical abstract. The corrected graphical abstract has been updated in the revised manuscript.

• In my opinion, qPCR data should be in the main document, to discuss with the editor

**Response:** We fully respect the reviewer’s opinion and agree that including the key qPCR data in the main manuscript can strengthen the presentation of our findings and facilitate discussion. To address this, we have moved the core qPCR results into the main document, where they are now presented alongside the corresponding protein expression data.

Revised sentence added to Materials and Methods:“*The relative gene expression was calculated by the 2^(-ΔΔCt) method and normalized to GAPDH. The primer sequences used for qRT-PCR are provided in Table 1.*”

**Table 1. All primer sequences were used for qRT-PCR in this work.**

| **Name** | **Forward** | **Reverse** |
| --- | --- | --- |
| ***MMP1*** | 5’-AAAATTACACGCCAGATTTGCC-3’ | 5’-GGTGTGACATTACTCCAGAGTTG-3’ |
| ***MMP13*** | 5’-ACTGAGAGGCTCCGAGAAATG-3’ | 5’-GAACCCCGCATCTTGGCTT-3’ |
| ***SOX9*** | 5’-AGCGAACGCACATCAAGAC-3’ | 5’-CTGTAGGCGATCTGTTGGGG-3’ |
| ***COL2*** | 5’-TGGACGATCAGGCGAAACC-3’ | 5’-GCTGCGGATGCTCTCAATCT-3’ |
| ***ACAN*** | 5’-GTGCCTATCAGGACAAGGTCT-3’ | 5’-GATGCCTTTCACCACGACTTC-3’ |
| ***IL6*** | 5’-ACTCACCTCTTCAGAACGAATTG-3’ | 5’-CCATCTTTGGAAGGTTCAGGTTG-3’ |
| ***IL1B*** | 5’-ATGATGGCTTATTACAGTGGCAA-3’ | 5’-GTCGGAGATTCGTAGCTGGA-3’ |
| ***TNF*** | 5’-CCTCTCTCTAATCAGCCCTCTG-3’ | 5’-GAGGACCTGGGAGTAGATGAG-3’ |
| ***OPA1*** | 5’-TGTGAGGTCTGCCAGTCTTTA-3’ | 5’-TGTCCTTAATTGGGGTCGTTG-3’ |
| ***MFN1*** | 5’-TGGCTAAGAAGGCGATTACTGC-3’ | 5’-GCAGGCTGGTGGTATTCTTG-3’ |
| ***MFN2*** | 5’-CTCTCGATGCAACTCTATCGTC-3’ | 5’-TCCTGTACGTGTCTTCAAGGAA-3 |
| ***FIS1*** | 5’-GTCCAAGAGCACGCAGTTTG-3’ | 5’-GCTGGTGATGGTCTTGGTG-3’ |
| ***GAPDH*** | 5’-GGAGCGAGATCCCTCCAAAAT-3' | 5’-GGCTGTTGTCATACTTCTCATGG-3’ |

**Location of the changes:** Row 158-162.

Some suggestions to improve discussion or the manuscript in general:

• You link AKT1 to mitochondrial dynamics but don't explain the "how."

**Response:** Thank you for this critical comment. We have comprehensively elaborated the regulatory mechanism between AKT1 and mitochondrial dynamics in the revised Discussion section. In this study, both molecular docking and AKT1 overexpression experiments verified that AKT1 is an essential molecular target of IQ. Beyond its roles in apoptosis and mitochondrial biogenesis via mTORC1/PGC-1α (refs., *Aging Dis.* **2022***, 13,* 157; *Front Med.* **2025**, *12*, 1635219), AKT1 controls mitochondrial fission through site-specific phosphorylation of DRP1 (refs., *ACS Omega*. **2023**, *8*, 45208–23). In IL‑1β-induced OA chondrocytes, chronic inflammatory stimulation triggers sustained, pathological hyperactivation of AKT1 (refs., *Bone Res*. **2019**, *7*, 23). In our OA model, IL-1β activates AKT1 to increase DRP1 Ser616 phosphorylation and drive mitochondrial fission. IQ inhibits AKT1 activity, and restores the balance of mitochondrial fusion and fission.

Revised sentence added to Discussion: “*The present work reveals that IQ restores chondrocyte functional homeostasis through a triple mechanism: (1) reversing IL-1β-induced proliferation arrest and shifting metabolism toward anabolism, (2) mitigating pro-inflammatory responses, and (3) normalizing mitochondrial dynamics. Critically, we identify AKT1 as a central node mediating these effects. Molecular docking predicts high-affinity binding of IQ to AKT1 (-10.4 kcal/mol), and AKT1 overexpression abrogates IQ’s protective benefits, confirming its necessity. However, the precise mechanism by which AKT1 coordinates mitochondrial dynamics deserves further elaboration. AKT1 bidirectionally regulates DRP1 phosphorylation: Ser616 modification promotes mitochondrial fission, while Ser637 modification restrains it, with the net effect dependent on cellular pathological status [42]. In our IL-1β-stimulated OA model, chronic inflammation triggers sustained aberrant AKT1 hyperactivation, upregulating DRP1 Ser616 phosphorylation and driving excessive pathological mitochondrial fission. IQ binds AKT1 to function as an allosteric modulator that dampens its pathological overactivation to balance mitochondrial fission and fusion. This molecular pathway accounts for the recovered mitochondrial membrane potential observed after IQ treatment. The excess unbound AKT1 from lentiviral overexpression exceeds IQ’s binding saturation limit, retaining pathological hyperactivity and eliminating IQ’s protective capacity, further confirming that IQ acts to normalize overactive AKT1.”*

**Location of the changes:** Row 536-552.

• What about enquiring/discussing effects of IQ on ROS?

**Response:** We appreciate the reviewer’s important comment. We have fully discussed the potential relationship between IQ and oxidative stress in the revised Discussion section. Oxidative stress driven by mitochondrial impairment and NOX activity is prominent in OA chondrocytes (refs., *Front Mol Biosci.* **2022**, *9,* 1001212; *Antioxidants.* **2022**, *11,* 2346). While ROS was not directly measured in this study, published studies have demonstrated the antioxidant property of IQ. Its aglycone quercetin can scavenge ROS and activate the Nrf2/ARE pathway, and IQ has been confirmed to lower ROS levels in hepatocytes and neurons (refs., *Int J Mol Sci.* **2025**, *26*, 2717; *Biomolecules.* **2019**, *9*, 468; *Chem Biol Interact*. **2018**, *284*, 32–40).

IL-1β triggers ROS production through NOX4 and damaged mitochondrial electron transport chain. The mitochondrial protective effect of IQ can indirectly restrain ROS release. Meanwhile, AKT1 modulates Nrf2 signaling and antioxidant gene expression (refs., *Antioxidants*. **2021**, *10*, 554). We therefore infer that IQ relieves oxidative stress via direct ROS clearance and AKT1-mediated Nrf2 activation. We have also put forward corresponding experimental plans, including DCFH-DA staining and Nrf2 pathway detection, for further validation in future work.

Revised sentence added to Discussion: “*Oxidative stress is a hallmark of OA chondrocytes, driven by mitochondrial dysfunction and nicotinamide adenine dinucleotide phosphate (NADPH) oxidase (NOX) activation [45,46]. We did not directly measure ROS levels in this study, but the literature strongly suggests that IQ is a potent antioxidant. Quercetin, the aglycone of IQ, directly scavenges ROS and upregulates nuclear factor erythroid 2-related factor 2/ antioxidant response element (Nrf2/ARE) pathway, while IQ itself reduces ROS in other cell types (e.g., hepatocytes, neurons) [25,47,48]. Given that IL-1β elevates ROS via NOX4 and mitochondrial electron transport chain leakage, IQ’s mitochondrial-stabilizing effect likely attenuates ROS production indirectly [48]. Furthermore, Furthermore, AKT1 signaling modulates Nrf2 nuclear translocation and antioxidant gene expression (e.g., Heme oxygenase-1 (HO‑1), NAD(P)H quinone dehydrogenase 1 (NQO1)) in a context‑dependent manner [49]. Therefore, IQ may suppress ROS through both direct scavenging and AKT1-mediated Nrf2 activation. Future experiments should quantify intracellular ROS (e.g., 2′,7′-Dichlorodihydrofluorescein diacetate (DCFH-DA) staining) and examine Nrf2 pathway activation (e.g., nuclear translocation, ARE-luciferase reporter) in IQ-treated chondrocytes.*”

**Location of the changes:** Row 553-565.

• Show a co-localization of TOM20 (a mitochondrial marker) and 8-OHdG (a marker of oxidative DNA damage).

**Response:** We thank the reviewer for this valuable suggestion, which helps strengthen the mechanistic link between mitochondrial dysfunction and oxidative DNA damage. We have now added immunofluorescence co-localization staining of TOM20 (mitochondrial marker) and 8-OHdG (oxidative DNA damage marker) to directly visualize this association, as shown in the revised **Fig S11**.

Briefly, IL-1β stimulation induced severe mitochondrial fragmentation and significantly increased 8-OHdG fluorescence intensity, with prominent co-localization of TOM20 and 8-OHdG, indicating robust mitochondrial oxidative DNA damage. IQ treatment notably restored mitochondrial morphology and reduced 8-OHdG accumulation, accompanied by decreased co-localization between TOM20 and 8-OHdG. Importantly, AKT1 overexpression completely abrogated the protective effects of IQ, re-inducing mitochondrial fragmentation and 8-OHdG enrichment, thus restoring high levels of co-localization. These results provide direct visual evidence that IQ attenuates IL-1β-induced mitochondrial oxidative DNA damage in an AKT1-dependent manner.

Revised sentence added to Results: “*To directly assess mitochondrial oxidative DNA damage, we performed immunofluorescence co-localization staining for 8-OHdG (a marker of oxidative DNA damage) and TOM20 (a mitochondrial membrane marker, Fig S11). Strong 8-OHdG fluorescence was observed in IL-1β-treated cells, with prominent co-localization with TOM20, indicating severe mitochondrial oxidative damage. IQ treatment significantly reduced 8-OHdG signals and their co-localization with TOM20, confirming that IQ attenuates IL-1β-induced mtDNA oxidation. However, AKT1 overexpression reversed these protective effects, restoring high levels of 8-OHdG and its co-localization with TOM20.”*

Revised sentence added to Materials and Methods: “*Immunofluorescence staining for TOM20 and 8-OHdG
C28/I2 were seeded on glass coverslips and treated with vehicle (Con), IL-1β (10 ng/mL), IL-1β + IQ (40 μM), or IL-1β + IQ + AKT1 overexpression. After treatment, cells were fixed with 4% paraformaldehyde for 15 min, permeabilized with 0.5% Triton X-100 in PBS for 10 min, and blocked with 5% bovine serum albumin (BSA) for 1 h at room temperature. Cells were then incubated overnight at 4°C with primary antibodies: rabbit anti-TOM20 (1:200, Abcam) and mouse anti-8-OHdG (1:500, Abcam). After washing, cells were incubated with Alexa Fluor 555-conjugated anti-rabbit IgG (1:500, Thermo Fisher) and Alexa Fluor 488-conjugated anti-mouse IgG (1:500, Thermo Fisher) for 1 h at room temperature. Nuclei were counterstained with DAPI (1 μg/mL) for 5 min. Images were acquired using an inverted fluorescence microscope (Leica DMIL LED, Leica Microsystems, Wetzlar, Germany). The mean fluorescence intensity (MFI) of 8-OHdG was quantified using ImageJ software (National Institutes of Health, Bethesda, MD, USA; https://imagej.net/ij/) [34].”*

**Fig. S11. IQ reduces IL-1β-induced mitochondrial oxidative DNA damage in chondrocytes via AKT1.** (A) Representative immunofluorescence images of TOM20 (red, mitochondria), 8-OHdG (green, oxidized DNA), and DAPI (blue, nuclei) in chondrocytes under different treatments. Scale bar: 100 μm. (B) Quantitative analysis of mean 8-OHdG fluorescence intensity. Data are mean ± SD (n = 3 independent experiments), with ****P < 0.0001 vs. Con; ####p < 0.0001 vs. IL-1β+IQ group. One-way analysis of variance (ANOVA) with Tukey’s post hoc test was used for multiple comparisons. **Location of the changes:** Row 174-186; 499-506; Fig. S11.

• Since your core claim involves mitochondrial dynamics, I suggest (if possible) to add a functional assay like membrane potential or ATP production via cytometry or fluorescence microscopy

**Response:** We appreciate this constructive suggestion. While we did not perform additional cytometry-based assays in the current study, we have already functionally validated mitochondrial integrity using JC-1 staining (**Figs 3I-J**), which directly reflects mitochondrial membrane potential—a core readout of mitochondrial function. Our Western blot results further demonstrated that IQ significantly restored the expression of fusion proteins (OPA1, MFN1/2) and suppressed fission-related proteins (FIS1, p-DRP1) (**Figs 3A-H**), consistent with improved mitochondrial homeostasis. Accumulating evidence confirms that restoring fusion-fission balance directly rescues mitochondrial function in chondrocytes (refs., *Kidney International*. **2021**, *99*, 870–84; *Phytomedicine.* **2025**, *141*, 156672). AKT1 overexpression fully reversed these protective effects (**Figs 7**), establishing a causal link between AKT1 inhibition and mitochondrial recovery. We agree that ATP production and ROS measurement would further strengthen the functional evidence, and these will be included in our future work.

Revised sentence added to Discussion: “*Future experiments should quantify intracellular ROS (e.g., 2′,7′-Dichlorodihydrofluorescein diacetate (DCFH-DA) staining) and examine Nrf2 pathway activation (e.g., nuclear translocation, ARE-luciferase reporter) in IQ-treated chondrocytes.*”

**Location of the changes:** Row 563-565.

PLOS ONE strongly encourages the upload of Original Uncropped Western Blot Images as Supporting Information, please add them, if available.

**Response:** All original Western blot images have been sorted and uploaded as supplementary files as required.

**Fig. S12. Original Western blot images for Fig 2.**

**Fig. S13. Original Western blot images for Fig 3.**

**Fig. S14. Original Western blot images for Fig 6.**

**Fig. S15. Original Western blot images for Fig 7.**

**Location of the changes:** Figs. S12-15.

Authors should contextualize their findings within broader nutraceutical or combinatorial anti-inflammatory strategies, just for example: In Vitro Effects of Low Doses of β-Caryophyllene, Ascorbic Acid and d-Glucosamine on Human Chondrocyte Viability and Inflammation, Mattiuzzo et al 2021.

**Response:** We appreciate this valuable suggestion. We supplemented concise contextual discussion of nutraceutical combinatorial therapy in Introduction, citing Mattiuzzo et al. (2021) as a typical combinatorial anti-inflammatory study to situate our IQ-related findings within this research field.

Revised sentence added to Introduction: “*Given this therapeutic gap, nutraceutical-derived small natural molecules have emerged as promising disease-modifying OA drug (DMOAD) candidates due to their superior biosafety and multitarget anti-inflammatory properties [18,19]. For instance, the combinatorial low-dose administration of β-caryophyllene, ascorbic acid, and D-glucosamine has been shown to alleviate inflammatory chondrocyte injury [20]*”

**Location of the changes:** Row 50-54.

Reviewer #2: The manuscript “Isoquercitrin Ameliorates Chondrocyte Dysfunction in Osteoarthritis by Targeting AKT1-Mediated Mitochondrial Dynamics Regulation” drew my attention because it investigates in detail the mechanisms of isoquercitrin protection for chondrocytes. The topic is relevant for the potential use of isoquercitrin as a therapeutic agent and adds to the evidence of its anti-inflammatory and protective activity.

**General response:** We sincerely thank Reviewer #2 for the positive evaluation, constructive comments, and valuable suggestions regarding our manuscript. We greatly appreciate the recognition of our work’s relevance and mechanistic depth, as well as the acknowledgment of isoquercitrin’s therapeutic potential in osteoarthritis. We have carefully addressed all raised points and revised the manuscript accordingly to further strengthen the clarity, rigor, and scientific soundness of our findings. Below, we provide our detailed, point-by-point responses.

Throughout the manuscript the word isoquercitrin and its abbreviation IQ are used interchangeably without obeying any apparent rule. Please correct this situation.

**Response:** We thank the reviewer for pointing out this issue. We have now standardized the usage of “isoquercitrin” and its abbreviation “IQ” throughout the manuscript following standard scientific writing conventions: “isoquercitrin” is written in full with the abbreviation “(IQ)” indicated at its first appearance in the main text, and “IQ” is used consistently thereafter. All inconsistent usages have been corrected in the revised manuscript.

Some abbreviations in the text, such as DMM, MIA, OPA and others are not explained. Please explain them.

**Response:** We appreciate the reviewer’s careful attention. We have now defined all previously unexplained abbreviations at their first occurrence in the text, including DMM, MIA, OPA1 and other relevant abbreviations. Beyond the above-mentioned terms, we have thoroughly reviewed the full text line by line to identify all abbreviations. Every acronym and shorthand term is now clearly annotated with its complete name at the first time of appearance, in strict compliance with PLOS ONE journal formatting requirements.

**Location of the changes:** Row 127-146; 148, 154, 191-194; 202; 209; 219-221; 224-228; 235-241; 244-248; 262; 390-392; 554; 556-557; 564; 572-573.

Materials and methods need to be explained in better detail, particularly in the situations mentioned bellow:

L75: please describe the model of microplate reader used, the wavelength at which measurements were made and the formula employed to calculate viability based on OD.

**Response:** Absorbance was quantified at 490 nm using a microplate reader (Multiskan FC, Thermo Fisher Scientific, USA).

Revised sentence added to Materials and Methods: “*After 2-4 h of incubation in the dark, optical density (OD) values were measured via a Multiskan FC microplate reader (Thermo Fisher Scientific, USA). Cell viability was calculated using the formula:*

$Viability\left( \% \right) = \left[ \frac{\left. {\left( {OD}_{Treated} \right.-OD}_{Blank} \right)}{\left( {OD}_{Control} \right.-\left. {OD}_{Blank} \right)} \right]$	*(1)”*

p

**Location of the changes:** Row 109-112.

L82: please describe the model of microscope you used; please explain in detail how you used the fluorescence images to calculate cell proliferation

**Response:** We greatly appreciate the reviewer’s valuable and constructive comments. Fluorescence images were acquired using an inverted fluorescence microscope (DMIL LED, Leica). We sincerely apologize for the large error bars and discrete data distribution in the original **Fig 1C**. This issue was caused by inappropriate statistical processing of the original datasets. We have re-analyzed data from independent biological replicates with correct statistical methods and updated **Fig 1C** accordingly. The exact number of replicates has also been clearly marked in the updated figure legend. Cell proliferation was quantified by counting the number of EdU‑positive cells per field with ImageJ; 3 random non‑overlapping fields were counted per sample in a blinded manner. The y‑axis label of **Fig** **1C** has been revised to Number of EdU‑positive cells per field for clarity. This counting method reliably reflects proliferation changes and supports our conclusion.

Revised sentence added to Materials and Methods: “*Fluorescence images were captured using an inverted fluorescence microscope (DMIL LED, Leica Microsystems, Wetzlar, Germany) equipped with a 10× objective lens. The number of EdU-positive proliferative cells were counted using ImageJ software (National Institutes of Health, Bethesda, MD, USA; https://imagej.net/ij/) [34].”*

**Fig 1. Preliminary chondroprotective evaluation of IQ. A.** DAPI/EDU co-staining of C28/I2 chondrocytes. DAPI (blue) stains cell nuclei, and EDU (green) labels proliferating cells (scale bar: 200 μm). **B.** Cell viability of C28/I2 chondrocytes treated with gradient concentrations of IQ without IL-1β stimulation. **p < 0.01, ****p < 0.0001 vs. 0 μM group. **C.** Quantification of EdU-positive cells. Data are presented as mean ± standard deviation (SD). **p < 0.01, ****p < 0.0001 vs. Con; ###p < 0.001 vs. IL-1β group. One-way ANOVA with Tukey’s post hoc test was used for multiple comparisons.

**Location of the changes:** Fig 1C; Row 121-124.

L97: please explain the ImageJ protocol you used for this analysis.

**Response:** We greatly appreciate the reviewer’s valuable and constructive comments. the membranes were observed by an enhanced chemiluminescence kit. Unmodified original images were quantified with ImageJ: images were converted to grayscale, background was subtracted uniformly, and relative protein expression was calculated as the integrated density of target band divided by that of β‑actin (internal control).

Revised sentence added to Materials and Methods: “*Finally, the membranes were observed by an enhanced chemiluminescence (ECL) kit (Millipore, Billerica, MA, USA) and quantified by ImageJ software (National Institutes of Health, Bethesda, MD, USA; https://imagej.net/ij/) [34]. β-actin was used as an internal control.”*

**Location of the changes:** Row 147-150.

Tables and figures are descriptive and effective in explaining the results. The conclusion is consistent with the results and arguments detailed in the Discussion section.

**Response:** We sincerely appreciate the reviewer’s positive evaluation of our tables, figures, conclusions and Discussion. We have carefully double-checked all figure legends, table annotations and concluding statements one more time to further ensure consistent logic, clear description and rigorous presentation throughout the manuscript, complying with the journal’s formatting standards.

Reviewer #3: Several citations appear to be incorrect or mismatched with the statements they are intended to support. A thorough and systematic revision of the reference list is required. The authors should carefully verify all citations, ensure correct matching between references and statements. Abstract needs to be revised. Methods should be added.

**General response:** We appreciate the reviewer’s careful comments. We have comprehensively checked and systematically revised all in-text citations and reference list to eliminate mismatches between content and references. Meanwhile, the Abstract has been thoroughly rewritten, and missing experimental details have been supplemented in the Methods section as required. All corresponding revisions are marked in the revised manuscript.

Introduction

Introduction on OA should be improved. It should be explained that OA is a whole joint disease involving all joint tissues. OA is characterized by subchondral bone remodelling), meniscal degeneration, inflammation of the synovial membrane and inflammation and biomechanical changes of the infrapatellar fat pad and ((doi.org/10.3389/fcell.2020.607764, DOI: 10.4081/ejh.2019.2998, DOI: 10.1080/03008207.2025.2502591 etc). Risk factors for OA should be added.

**Response:** We thank the reviewer for valuable suggestions. We have fully restructured the Introduction section by referencing multiple high-quality published OA articles following standard high-impact journal writing logic:

1. We supplemented the modern whole-joint disease conception of OA, detailed four characteristic intra-articular pathological lesions and mainstream risk factors as required, and cited the three recommended literatures.

2. We built rigorous logical linkage: pathological mediators derived from multi-tissue lesions of whole-joint OA induce chondrocyte mitochondrial dysfunction, which bridges systemic joint inflammation and final cartilage degeneration, reasonably consolidating the rationality of our mitochondrial-targeted research.

3. We contextualized our IQ findings within the broader nutraceutical combinatorial anti-inflammatory field. All revisions are highlighted in revised manuscript.

Revised sentence added to Introduction: “*Osteoarthritis (OA) is one of the most widespread whole-joint degenerative diseases globally, imposing a substantial socioeconomic burden due to chronic joint pain and locomotor disability in aging populations [1–3]. Contemporary pathological consensus has advanced beyond the outdated "single-cartilage wear" view, defining OA as a heterogeneous whole-joint disorder characterized by abnormal subchondral bone remodelling, progressive meniscal matrix deterioration, persistent synovial inflammation, and inflammatory infiltration of the infrapatellar fat pad [4–7]. Major predisposing risk factors include advanced age, obesity, mechanical overloading, articular trauma, and systemic metabolic dysfunction [8]. Importantly, the traditional wear-driven theory fails to account for progressive cartilage loss in a significant subset of OA patients without excessive joint loading, suggesting that non-mechanical inflammatory and intracellular pathological cascades play a dominant role in disease progression [9,10].”*

**Location of the changes:** Row 26-36.

Lines 29-32: please check the references as they seem to be not appropriate.

**Response:** We sincerely appreciate the reviewer’s careful inspection. We have entirely removed the original mismatched citations and revised the corresponding introductory description strictly following modern OA whole-joint disease consensus. Meanwhile, all reference formats have been standardized in full compliance with PLOS ONE’s citation guidelines. All updated references (Ref 1–10, published mostly from 2019–2025) are well-matched to each textual statement:

Refs 4–7 directly cover four core intra-articular pathological changes (subchondral bone remodeling, meniscal degeneration, synovitis, infrapatellar fat pad inflammatory/biomechanical abnormality), consistent with our description;

Ref 8 supports the listed major OA risk factors including aging, obesity, trauma and metabolic dysfunction;

Refs 9–10 provide solid evidence that non-inflammatory intracellular pathways (oxidative stress, programmed cell death etc.) drive OA progression independent of mechanical overload.

Revised references: “

[1] Berenbaum F, Walker C. Osteoarthritis and inflammation: a serious disease with overlapping phenotypic patterns. Postgrad Med. 2020 May 18;132(4):377–84. doi:10.1080/00325481.2020.1730669 PubMed PMID: 32100608.
[2] Zhang W, Qi L, Chen R, He J, Liu Z, Wang W, et al. Circular RNAs in osteoarthritis: indispensable regulators and novel strategies in clinical implications. Arthritis Res Ther. 2021 Jan 12;23(1):23. doi:10.1186/s13075-021-02420-2
[3] Wakale S, Wu X, Sonar Y, Sun A, Fan X, Crawford R, et al. How are aging and osteoarthritis related? Aging Dis. 2023;14(3):592. doi:10.14336/AD.2022.0831
[4] Najar M, Fahmi H. Of mesenchymal stem/stromal cells and osteoarthritis: Time to merge the latest breakthroughs. Stem Cell Rev Rep. 2020 Oct 1;16(5):1016–8. doi:10.1007/s12015-020-10001-0
[5] Pettenuzzo S, Berardo A, Belluzzi E, Pozzuoli A, Ruggieri P, Carniel EL, et al. Mechanical insights into fat pads: a comparative study of infrapatellar and suprapatellar fat pads in osteoarthritis. Connect Tissue Res. 2025 Jul 4;66(4):272–83. doi:10.1080/03008207.2025.2502591 PubMed PMID: 40340764.
[6] Battistelli M, Favero M, Burini D, Trisolino G, Dallari D, De Franceschi L, et al. Morphological and ultrastructural analysis of normal, injured and osteoarthritic human knee menisci. Eur J Histochem. 2019 Feb 11;63(1). doi:10.4081/ejh.2019.2998
[7] Zhu X, Chan YT, Yung PSH, Tuan RS, Jiang Y. Subchondral bone remodeling: A therapeutic target for osteoarthritis. Front Cell Dev Biol. 2021 Jan 21;8:607764. doi:10.3389/fcell.2020.607764
[8] Martel-Pelletier J, Maheu E, Pelletier JP, Alekseeva L, Mkinsi O, Branco J, et al. A new decision tree for diagnosis of osteoarthritis in primary care: international consensus of experts. Aging Clin Exp Res. 2019 Jan 1;31(1):19–30. doi:10.1007/s40520-018-1077-8
[9] Liu S, Zhang G, Li N, Wang Z, Lu L. The interplay of aging and PANoptosis in osteoarthritis pathogenesis: Implications for novel therapeutic strategies. J Inflamm Res. 2025 Feb 10;18:1951–67. doi:10.2147/JIR.S489613
[10] Yadav S, Yadav J, Humphrey MB. Emerging role of TRP channels in osteoarthritis pathogenesis. Cells. 2026 Jan;15(3):299. doi:10.3390/cells15030299*”*

**Location of the changes:** Refs 1–10.

Lines 32-34: reference 7 is paper focused on the role of pirfenidone in attenuating synovial fibrosis. Please correct. The same for reference 8.

**Response:** We sincerely apologize for the mismatched citations in the original Lines 32–34. We have thoroughly revised the relevant introductory text and fully replaced the inappropriate Reference 7 and Reference 8 with two well-aligned literatures.

The newly updated references systematically illustrate that non-mechanical inflammatory signals and intracellular cell death pathways drive OA development independent of mechanical overloading, which strongly supports our revised narrative. The modified sentence in the Introduction now reads: “*Importantly, the traditional wear-driven theory fails to account for progressive cartilage loss in a significant subset of OA patients without excessive joint loading, suggesting that non-mechanical inflammatory and intracellular pathological cascades play a dominant role in disease progression [9,10].*”

The two updated supporting references are listed below in journal-standard format:

[9] Liu S, Zhang G, Li N, Wang Z, Lu L. The interplay of aging and PANoptosis in osteoarthritis pathogenesis: Implications for novel therapeutic strategies. J Inflamm Res. 2025 Feb 10;18:1951–67. https://doi.org/10.2147/JIR.S489613

[10] Yadav S, Yadav J, Humphrey MB. Emerging role of TRP channels in osteoarthritis pathogenesis. Cells. 2026 Jan;15(3):299. https://doi.org/10.3390/cells15030299

**Location of the changes:** Row 33-36; Refs 9 and 10.

Lines 35-37: please check the references as they do not appear to support the concept.

**Response:** We apologize for previous mismatched citations. We have removed two irrelevant references and updated Refs.11–13 with three validated articles shown above, among which we retain the 2023 Arthritis Res Ther paper (Prokineticin 2-related study) plus the 2021 Sci Rep and 2022 Cell Death Dis articles to fully support this sentence:

Our manuscript: “*The core pathological hallmark of OA lies in chondrocyte anabolic-catabolic dysregulation: marked by diminished extracellular matrix (ECM) production and heightened secretion of matrix-degrading enzymes [11–13].”*

Supporting evidence extracted verbatim from cited references:

1. Yang et al., 2023 (*Arthritis Res Ther*): a hallmark of OA is progressive articular cartilage destruction, primarily regulated by chondrocytes through the production of matrix-degrading enzymes and/or the downregulation of cartilage extracellular matrix (ECM) molecules.

2. Li et al., 2022 (*Cell Death Dis*): The hallmark of OA is cartilage matrix breakdown, which is caused by excessive matrix-degrading enzyme activity and/or downregulation of chondrocyte extracellular matrix (ECM) molecules.

3. Hwang et al., 2021 (*Sci Rep*): The hallmark of osteoarthritis (OA) is destruction of the articular cartilage, which results from the disturbance of the equilibrium between the anabolic and catabolic activities of chondrocytes and loss of the extracellular matrix (ECM).

All three original introduction excerpts perfectly match our description of reduced ECM synthesis and elevated matrix-degrading enzymes resulting from anabolic-catabolic imbalance of chondrocytes. All citation revisions and corresponding text proof are highlighted in red within the revised manuscript.

Revised references: “

[11] Yang J, Shin Y, Kim HJ, Kim HE, Chun JS. Prokineticin 2 is a catabolic regulator of osteoarthritic cartilage destruction in mouse. Arthritis Res Ther. 2023 Dec 6;25(1):236. doi:10.1186/s13075-023-03206-4
[12] Li R, Guan Z, Bi S, Wang F, He L, Niu X, et al. The proton-activated G protein-coupled receptor GPR4 regulates the development of osteoarthritis via modulating CXCL12/CXCR7 signaling. Cell Death Dis. 2022 Feb 14;13(2):152. doi:10.1038/s41419-021-04455-4
[13] Hwang HS, Lee MH, Kim HA. The TLR-2/TonEBP signaling pathway regulates 29-kDa fibronectin fragment-dependent expression of matrix metalloproteinases. Sci Rep. 2021 Apr 26;11(1):8891. doi:10.1038/s41598-021-87813-8*”*

**Location of the changes:** Refs 11 -13.

Line 37: authors should explain that infrapatellar fat pad and synovial membrane are inflamed and secretes pro-inflammatory cytokines.

**Response:** Thank you for the valuable suggestion. We have supplemented relevant descriptions in the manuscript as required. The revised content clarifies that inflamed synovial membrane and infrapatellar fat pad (IFP) release core pro-inflammatory cytokines (IL-1β, TNF-α) to aggravate chondrocyte metabolic imbalance, forming a complete pathological chain linking soft tissue inflammation, cytokine release, chondrocyte catabolism and mitochondrial injury.

Revised sentence added to Introduction: “*Crucially, this dysregulation is exacerbated by the inflamed infrapatellar fat pad (IFP) and synovial membrane, which secrete a plethora of pro-inflammatory cytokines, such as interleukin-1β (IL-1β) and tumor necrosis factor-α (TNF-α), into the joint cavity [14, 15].”*

Revised references: “

[14] Harvanova D, Matejova J, Slovinska L, Lacko M, Gulova S, Fecskeova LK, et al. The role of synovial membrane in the development of a potential in vitro model of osteoarthritis. Int J Mol Sci. 2022 Jan;23(5):2475. doi:10.3390/ijms23052475
[15] Mallia I, Fioravanti A, Guiducci S. Infrapatellar fat pad in knee osteoarthritis: A comprehensive review of pathophysiology and targeted therapeutic strategies. Int J Mol Sci. 2025 Oct 26;26(21):10408. doi:10.3390/ijms262110408

**Location of the changes:** Row 39-42; Refs 14 and 15.

Lines 39-40: please provide appropriate references.

**Response:** Thank you for your careful suggestion. We have removed irrelevant citations and substituted them with two well-matched published articles to provide solid evidence for this statement. The original core content extracted verbatim from the two references is summarized below to confirm citation validity:

Our manuscript: *Parallel to this, pro-inflammatory cytokines perpetuate this imbalance by stimulating catabolic signaling cascades, while disrupted mitochondrial dynamics further worsen chondrocyte dysfunction via excessive reactive oxygen species (ROS) generation [16,17].*

Supporting evidence extracted verbatim from cited references:

1. Wei et al., 2020 (Chin Med): IL-1beta is known in reducing mitochondrial oxidative phosphorylation by switching to glycolysis and producing excessive reactive oxygen species (ROS) in chondrocytes through damaging mitochondrial structure, dynamics, and genome stability, resulting in mitochondrial dysfunction and oxidative stress, which are two main hallmarks of OA.

2. Liu et al., 2026 (Front Immunol): mitochondrial dysfunction-including impaired oxidative phosphorylation (OXPHOS), excessive reactive oxygen species (ROS) generation, disrupted mitochondrial dynamics, and dysregulated mitophagy-as an early and pivotal driver of OA pathogenesis. These bioenergetic failures not only disrupt chondrocyte metabolism but also amplify inflammation, matrix degradation, and cell death.

Both excerpts collectively validate our description: pro-inflammatory cytokine (IL‑1β) disrupts mitochondrial dynamics to induce excess ROS and chondrocyte dysfunction, and aberrant mitochondrial homeostasis further aggravates cartilage catabolism and metabolic imbalance in OA. All updated citation labels [16,17] are highlighted in red in the revised manuscript.

Revised references: “

[16] Liu Y, Liu Y, Zhang N, An H, Mi L, Gao Y, et al. Mitochondrial transplantation for osteoarthritis: from molecular mechanisms to clinical translation. Front Immunol. 2026 Mar 23;17. doi:10.3389/fimmu.2026.1716906
[17] Wei Z, Dong C, Guan L, Wang Y, Huang J, Wen X. A metabolic exploration of the protective effect of Ligusticum wallichii on IL-1β-injured mouse chondrocytes. Chin Med. 2020 Feb 1;15(1):12. doi:10.1186/s13020-020-0295-0*”*

**Location of the changes:** Refs 16 and 17.

Lines 43-44: please provide appropriate references.

**Response:** Thank you for your valuable suggestion. We have polished the original sentence into: “*Given this therapeutic gap, nutraceutical-derived small natural molecules have emerged as promising disease-modifying OA drug (DMOAD) candidates due to their superior biosafety and multitarget anti-inflammatory properties [18,19].*” and updated citations to the two matching literatures: Ahmed et al. (2026, Biomed Pharmacother) and Sukhikh et al. (2021, Molecules). These two up-to-date publications strongly support our viewpoint regarding natural flavonoids as prospective DMOAD candidates with good safety and multi-target anti-inflammatory advantages.

Revised references: “

[18] Ahmed S, Comolli NK. Delivery of potential natural disease-modifying osteoarthritis drugs: Beyond polyphenols and flavonoids. Biomed Pharmacother. 2026 May 1;198:119154. doi:10.1016/j.biopha.2026.119154
[19] Sukhikh S, Noskova S, Ivanova S, Ulrikh E, Izgaryshev A, Babich O. Chondroprotection and molecular mechanism of action of phytonutraceuticals on osteoarthritis. Molecules. 2021 Jan;26(8):2391. doi:10.3390/molecules26082391*”*

**Location of the changes:** Row 50-52; Refs 18 and 19.

Line 46: please define “IQ”.

**Response:** Thanks for the reminder. We have supplemented the full definition of IQ in the revised manuscript as: “*Isoquercitrin (IQ), a naturally occurring flavonol glycoside, exhibits potent antioxidant and anti-inflammatory capacities and protects against various pathological states (e.g., ischemia-reperfusion injury, neurodegeneration).*”

**Location of the changes:** Row 54.

Lines 46-48: please verify that all references are focused on OA.

**Response:** Thank you for your careful check. Our manuscript: “*IQ has better bioavailability than its parent molecule, yet its function in OA has been scarcely investigated [28–30].”*

We aim to illustrate two points: IQ possesses superior bioavailability compared with quercetin, and relevant research exploring IQ’s role in OA remains limited. To optimize citation matching, we have removed two irrelevant articles focusing on cardiovascular allergy and intestinal injury, and supplemented one newly published OA-focused literature into the reference list.

After revision, references for IQ’s general pharmacological activity match non-OA disease studies reasonably, while updated citations supporting bioavailability partly include OA-related research, consistent with our statement that IQ-related OA research is still insufficient.

Revised references: “

[30] Li H, Chen G, Ma C, Hu Y, Zhou P, Zhou S, et al. Isoquercitrin attenuates osteoarthritis progression by targeting P53-mediated ferroptosis: A mechanistic study integrating network pharmacology and experimental validation. J Nutr Biochem. 2026 Jul 1;153:110306. doi:10.1016/j.jnutbio.2026.110306*”*

**Location of the changes:** Refs 30.

Line 50: please define AKT1.

**Response:** Thank you for the comment. We have supplemented the definition of AKT1 in the sentence as required, revised text is shown below: “*Notably, RAC-alpha serine/threonine-protein Kinase (AKT1) is a master upstream modulator governing chondrocyte mitochondrial dynamics and metabolic homeostasis [31–33].*”

**Location of the changes:** Row 63.

Line 52: please verify that these references are appropriate.

**Response:** Thank you for your careful verification. We have revised the original sentence and updated supporting references to ensure full content matching, the optimized sentence reads: “*Notably, RAC-alpha serine/threonine-protein Kinase (AKT1) is a master upstream modulator governing chondrocyte mitochondrial dynamics and metabolic homeostasis [31–33]. Persistent IL-1β stimulation triggers pathological sustained hyperactivation of AKT1, inducing excessive mitochondrial fission [32]. Whether IQ directly targets AKT1 as an allosteric buffer to normalize its overactive signaling and alleviate IL-1β-mediated mitochondrial and cartilage catabolic defects has not been experimentally verified.*”

The rationality of three newly replaced citations is detailed below:

Ref.31 (Manning BD, Cell 2017): This authoritative review systematically defines AKT (PKB) as a core serine/threonine kinase regulating cellular metabolism across multiple tissues, laying the canonical molecular background for AKT1 biological function.

Ref.32 (Xie J, Bone Res 2019): This OA-focused original paper confirms aberrant sustained AKT activation triggers chondrocyte oxidative stress, mitochondrial dysfunction and subsequent OA lesion, directly validating AKT1’s regulatory role on chondrocyte mitochondrial homeostasis in osteoarthritic cartilage.

Ref.33 (Xu C, Phytomedicine 2025): The literature demonstrates small-molecule natural compound binds AKT1 to modulate mitochondrial dynamics and improve organ injury, supporting our core logic that phytochemicals exert mitochondrial-protective effects via AKT1 targeting.

All three literatures jointly back our description of AKT1 function and the pending research gap regarding IQ-AKT1 interaction.

Revised sentence added to references: “

[31] Manning BD, Toker A. AKT/PKB signaling: Navigating the network. Cell. 2017 Apr 20;169(3):381–405. doi:10.1016/j.cell.2017.04.001 PubMed PMID: 28431241.
[32] Xie J, Lin J, Wei M, Teng Y, He Q, Yang G, et al. Sustained Akt signaling in articular chondrocytes causes osteoarthritis via oxidative stress-induced senescence in mice. Bone Res. 2019 Aug 5;7(1):23. doi:10.1038/s41413-019-0062-y
[33] Xu C, Wang H, Wang H, Man J, Deng Y, Li Y, et al. Schisandrin B regulates mitochondrial dynamics via AKT1 activation and mitochondrial targeting to ameliorate renal ischemia-reperfusion injury. Phytomedicine. 2025 Jun 1;141:156672. doi:10.1016/j.phymed.2025.156672

**Location of the changes:** Row 63-68; Refs 31-33.

At the end of the introduction, authors should report the aim/s of the study rather that the results.

**Response:** We have completely revised the concluding paragraph of Introduction to exclusively describe the research objectives instead of final experimental outcomes as requested, revised text is shown below: “*To fill the above-mentioned research gap that the direct interaction between IQ and AKT1 as well as the downstream chondroprotective cascade remains unclarified in OA pathogenesis, the present study was designed to explore the chondroprotective efficacy and underlying molecular mechanism of IQ against OA progression. Specifically, we will employ an IL-1β-stimulated OA-like chondrocyte model to evaluate the impact of IQ on metabolic homeostasis, inflammatory responses, and mitochondrial integrity. Furthermore, we will utilize molecular docking and dynamic simulation analyses to identify high-affinity targets of IQ, followed by gain-of-function experiments to validate AKT1 as a key mediator. Ultimately, we seek to elucidate the mechanistic link between AKT1 inhibition, restoration of mitochondrial dynamics, and rebalancing of chondrocyte metabolism. Our findings are expected to establish IQ as a DMOAD candidate and provide a novel mechanistic framework for OA therapeutic intervention.*”

**Location of the changes:** Row 69-79.

Methods

Supplier of all substances and kits used should be provided.

**Response:** We are grateful for this insightful comment regarding reagent information. We have supplemented complete supplier information for all experimental reagents, kits, cell lines, lentivirus and consumables throughout the Materials and Methods section following standard SCI formatting specifications, including manufacturer name, city/country for all chemicals, antibodies, detection kits and biological materials.

Revised sentence added to Materials and Methods: “*Materials
C28/I2 human chondrocyte was purchased from Shanghai Yaji Biotechnology (Shanghai, China). Isoquercitrin (IQ, Cat.SM2187, ≥98% purity) was obtained from Beyotime Biotechnology (Shanghai, China). Recombinant human IL-1β was sourced from R&D Systems (MN, USA). DMEM medium, FBS and penicillin-streptomycin were supplied by Gibco (USA); trypsin, Polybrene and Triton X-100 were from Sigma-Aldrich (USA). CCK-8, EdU, JC-1 (Cat.D-9113), RIPA lysis buffer and DAPI staining kits were purchased from Dojindo, Beyotime and Bioss Biotechnology (Beijing, China), respectively. SDS-PAGE kit, BCA quantification kit and PVDF membrane were from Epizyme and Millipore (USA). All primary antibodies were acquired from Proteintech, MedChemExpress and Abcam; HRP-conjugated secondary antibodies were obtained from Jackson ImmunoResearch (USA). RNA extraction, reverse transcription and qPCR related kits were products of Takara (Japan) and GenStar (China). Lentiviruses for AKT1 overexpression and empty vector were constructed by Shanghai Jima Pharmaceutical Technology (Shanghai, China). GraphPad Prism, R v4.3.3, Cytoscape 3.8.2 and Gromacs 2022 software were used for statistical calculation, network construction and molecular dynamics simulation.”*

**Location of the changes:** Row 81-96.

Line 75: please add the instrument used.

**Response:** We have added the corresponding instrument information as required, and supplemented relevant details for OD value detection.

Revised sentence added to Materials and Methods: “*After 2-4 h of incubation in the dark, optical density (OD) values were measured via a Multiskan FC microplate reader (Thermo Fisher Scientific, USA). Cell viability was calculated using the formula:*

$Viability\left( \% \right) = \left[ \frac{\left. {\left( {OD}_{Treated} \right.-OD}_{Blank} \right)}{\left( {OD}_{Control} \right.-\left. {OD}_{Blank} \right)} \right]$	*(1)”*

p

**Location of the changes:** Row 109-112.

Line 77: please add seeded cells.

**Response:** Thank you for your careful review. In accordance with the standard terminology for cell experiments, we have revised the sentence and used the professional expression "seeded cells" to describe cell inoculation.

Revised sentence added to Materials and Methods: “*C28/I2 cells were seeded at a density of 3 × 10³ cells per well in 96-well plates containing 100 μL medium and incubated for 24 h.*

*C28/I2 cells were seeded in 96-well plates and treated with IL-1β and/or IQ at 70% confluence for 24 h per group allocation.”*

**Location of the changes:** Row 105-106; 114.

Lines 82 and 111: please add microscope used.

**Response:** We thank the reviewer for pointing out these missing details in the Materials and Methods section. According to your suggestion, we have added the detailed information of the fluorescence microscope used for image observation. The updated text reads as follows: “*Fluorescence images were captured using an inverted fluorescence microscope (DMIL LED, Leica Microsystems, Wetzlar, Germany) equipped with a 10× objective lens.*

*Fluorescence signals were visualized using an inverted fluorescence microscope (Leica DMIL LED, Leica Microsystems, Wetzlar, Germany), with red fluorescence (JC-1 aggregates) and green fluorescence (JC-1 monomers) detected at excitation/emission wavelengths of 525/590 nm and 490/530 nm, respectively.*”

**Location of the changes:** Row 121-122;169-173.

Line 84: please add buffer and inhibitors used.

**Response:** We appreciate your careful review. Considering that protease and phosphatase inhibitors are essential to prevent protein degradation and maintain phosphorylation levels during protein extraction, we have added the relevant information of RIPA lysis buffer and matched inhibitors.

Revised sentence added to Materials and Methods: “*Total protein was isolated using radio-immunoprecipitation assay (RIPA) lysis buffer containing protease and phosphatase inhibitor cocktails. C28/I2 chondrocytes were collected, rinsed with cold PBS, lysed on ice, and centrifuged to harvest supernatants. Protein concentration was quantified via the bicinchoninic acid (BCA) assay. Equal amounts of 30 µg protein per sample were separated on two distinct SDS-PAGE gels according to target molecular weights: 6% constant gels for high-molecular-weight proteoglycans (e.g., aggrecan (ACAN)), and 12% constant gels for low-molecular-weight proteins.”*

**Location of the changes:** Row 126-133.

Line 85: how did authors quantify the proteins? How many micrograms of protein were loaded? Did authors use SDS-PAGE? Percentage?

**Response:** Thank you for your careful check. Relevant details about protein quantification, loading quantity and SDS-PAGE gels have been clearly stated at Line 85. We used the BCA assay to measure protein concentration, and 30 μg of protein was loaded into each lane of 6% or 12% non-gradient SDS-PAGE gels.

Revised sentence added to Materials and Methods: “*T Total protein was isolated using radio-immunoprecipitation assay (RIPA) lysis buffer containing protease and phosphatase inhibitor cocktails. C28/I2 chondrocytes were collected, rinsed with cold PBS, lysed on ice, and centrifuged to harvest supernatants. Protein concentration was quantified via the bicinchoninic acid (BCA) assay. Equal amounts of 30 µg protein per sample were separated on two distinct SDS-PAGE gels according to target molecular weights: 6% constant gels for high-molecular-weight proteoglycans (e.g., aggrecan (ACAN)), and 12% constant gels for low-molecular-weight proteins.”*

**Location of the changes:** Row 126-133.

Line 86: please specify the membrane used.

**Response:** We appreciate your careful review. As suggested, we have explicitly indicated that polyvinylidene fluoride (PVDF) membranes were used for protein transfer.

Revised sentence added to Materials and Methods: “*After transfer to polyvinylidene fluoride (PVDF) membranes, membranes were blocked with 5% (w/v) bovine serum albumin (BSA) dissolved in TBST for 1 hour at room temperature, rinsed with TBST, and incubated overnight at 4 °C with primary antibodies.”*

**Location of the changes:** Row 133-136.

Why did authors used skim milk to block the membranes? This is no appropriate when using phosphoantibodies.

**Response:** We greatly appreciate your careful inspection and professional comment. We apologize sincerely for this inappropriate description in the manuscript.As is well known, skim milk is not suitable for phosphoprotein detection. Fully aware of this experimental principle, our research team never used skim milk for membrane blocking in any WB experiment. The mention of skim milk was a pure writing mistake when organizing the Methods section, which did not reflect the actual experimental protocol. We have now corrected this mistake in the revised manuscript.

Revised sentence added to Materials and Methods: “*After transfer to polyvinylidene fluoride (PVDF) membranes, membranes were blocked with 5% (w/v) bovine serum albumin (BSA) dissolved in TBST for 1 hour at room temperature, rinsed with TBST, and incubated overnight at 4 °C with primary antibodies.”*

**Location of the changes:** Row 133-136.

Line 95” dilution unspecified” is unclear. Please specify the antibodies and the dilution.

**Response:** We sincerely appreciate the reviewer for these constructive suggestions. We have supplemented all missing experimental details for the Western blotting section in the revised manuscript. All relevant parameters have been added and clearly described to ensure experimental reproducibility.

Revised sentence added to Western Blotting:“*After transfer to polyvinylidene fluoride (PVDF) membranes, membranes were blocked with 5% (w/v) bovine serum albumin (BSA) dissolved in TBST for 1 hour at room temperature, rinsed with TBST, and incubated overnight at 4 °C with primary antibodies (β-actin: 66009-1-Ig, 1:10000, Proteintech; matrix metallopeptidase 1 (MMP-1): HY-P80754, 1:1000, MedChemExpress; matrix metallopeptidase 13 (MMP-13): YA3143, 1:1000, MedChemExpress; SRY-box transcription factor 9 (SOX9): HY-P80355, 1:500, MedChemExpress; type II collagen (COL2): HY-P81047, 1:2000, MedChemExpress; aggrecan (ACAN): ab3778, 1:1000, Abcam; interleukin 6 (IL-6): ab233706, 1:1000, Abcam; interleukin 1 beta (IL-1β): HY-P80503, 1:1000, MedChemExpress; tumor necrosis factor alpha (TNF-α): HY-P80914, 1:800, MedChemExpress; optic atrophy 1 (OPA1): ab42364, 1:1000, Abcam; mitofusin 1 (MFN1): ab221661, 1:1000, Abcam; mitofusin 2 (MFN2): ab56889, 1:1000, Abcam; mitochondrial fission 1 protein (FIS1): ab156865, 1:10000, Abcam; dynamin-related protein 1 (DRP1): 12957-1-AP, 1:5000, Proteintech; DRP1 (phospho S616): ab314755, 1:1000, Abcam). Then, membranes were incubated with horseradish peroxidase (HRP)-conjugated anti-rabbit or anti-mouse secondary antibodies (1:5000) for 1 h at room temperature.*”

**Location of the changes:** Row 133-147.

Line 96: please add kit used for the developing.

**Response:** We have added the name and manufacturer of the ECL detection kit used for signal development at Line 96 to complete the experimental details.

Revised sentence added to Western Blotting:“*Finally, the membranes were observed by an enhanced chemiluminescence (ECL) kit (Millipore, Billerica, MA, USA) and quantified by ImageJ software (National Institutes of Health, Bethesda, MD, USA; https://imagej.net/ij/) [34].*”

**Location of the changes:** Row 147-150.

Line 97: Please ensure that the imagej software is properly cited and referenced.

The primers sequence should be added.

**Response:** We have revised the description of ImageJ software and added the corresponding reference to meet the citation requirements. In addition, we noticed that the primer sequences were only available in the supporting information before. Following your suggestion, we have relocated these sequences to the main text and compiled them as Table 1 for better readability.

Revised sentence added to Western Blotting:“*Finally, the membranes were observed by an enhanced chemiluminescence (ECL) kit (Millipore, Billerica, MA, USA) and quantified by ImageJ software (National Institutes of Health, Bethesda, MD, USA; https://imagej.net/ij/) [34].*”

Revised sentence added to Materials and Methods:“*The relative gene expression was calculated by the 2^(-ΔΔCt) method and normalized to GAPDH. The primer sequences used for qRT-PCR are provided in Table 1.*”

**Table 1. All primer sequences were used for qRT-PCR in this work.**

| **Name** | **Forward** | **Reverse** |
| --- | --- | --- |
| ***MMP1*** | 5’-AAAATTACACGCCAGATTTGCC-3’ | 5’-GGTGTGACATTACTCCAGAGTTG-3’ |
| ***MMP13*** | 5’-ACTGAGAGGCTCCGAGAAATG-3’ | 5’-GAACCCCGCATCTTGGCTT-3’ |
| ***SOX9*** | 5’-AGCGAACGCACATCAAGAC-3’ | 5’-CTGTAGGCGATCTGTTGGGG-3’ |
| ***COL2*** | 5’-TGGACGATCAGGCGAAACC-3’ | 5’-GCTGCGGATGCTCTCAATCT-3’ |
| ***ACAN*** | 5’-GTGCCTATCAGGACAAGGTCT-3’ | 5’-GATGCCTTTCACCACGACTTC-3’ |
| ***IL6*** | 5’-ACTCACCTCTTCAGAACGAATTG-3’ | 5’-CCATCTTTGGAAGGTTCAGGTTG-3’ |
| ***IL1B*** | 5’-ATGATGGCTTATTACAGTGGCAA-3’ | 5’-GTCGGAGATTCGTAGCTGGA-3’ |
| ***TNF*** | 5’-CCTCTCTCTAATCAGCCCTCTG-3’ | 5’-GAGGACCTGGGAGTAGATGAG-3’ |
| ***OPA1*** | 5’-TGTGAGGTCTGCCAGTCTTTA-3’ | 5’-TGTCCTTAATTGGGGTCGTTG-3’ |
| ***MFN1*** | 5’-TGGCTAAGAAGGCGATTACTGC-3’ | 5’-GCAGGCTGGTGGTATTCTTG-3’ |
| ***MFN2*** | 5’-CTCTCGATGCAACTCTATCGTC-3’ | 5’-TCCTGTACGTGTCTTCAAGGAA-3 |
| ***FIS1*** | 5’-GTCCAAGAGCACGCAGTTTG-3’ | 5’-GCTGGTGATGGTCTTGGTG-3’ |
| ***GAPDH*** | 5’-GGAGCGAGATCCCTCCAAAAT-3' | 5’-GGCTGTTGTCATACTTCTCATGG-3’ |

**Location of the changes:** Row 147-150; 159-162. Table 1

Line 108: how many cells?

**Response:** Thanks for your valuable reminder. We have supplemented the specific cell seeding densities for all cell-based functional assays to ensure experimental repeatability. Specifically, we added the cell number for the EdU staining assay (96-well plates, 3 × 10³ cells/well) and JC-1 staining assay (24-well plates, 1 × 10⁵ cells/well).

Revised sentence added to Cell counting kit‑8 (CCK-8) assay for IQ treatment concentration:“*C28/I2 cells were seeded at a density of 3 × 10³ cells per well in 96-well plates containing 100 μL medium and incubated for 24 h.*”

Revised sentence added to EdU Staining for Cell Proliferation:“*C28/I2 cells were seeded in 96-well plates at 3×10³ cells/well and treated with IL-1β and/or IQ at 70% confluence for 24 h per group allocation.*”

Revised sentence added to JC-1 Staining:“*C28/I2 cells were seeded in 24-well plates at 1 × 10⁵ cells per well and treated with IL-1β and/or IQ at 70% confluence for 24 h.*”

**Location of the changes:** Row 105-106; 114-115; 164-165.

The section target collection isoquercitrin should be improved.

**Response:** We have fully revised and supplemented the section of isoquercitrin target collection. According to our actual experimental records, we added the PubChem ID of isoquercitrin, specific screening thresholds for each database, predicted target numbers and total non-redundant targets. We also clarified the human species limitation and data processing procedures. This part is now complete and reproducible.

Revised sentence added to Target Collection of IQ:“*The chemical structure of IQ was retrieved from the PubChem database (CID: 5280804). Five online databases were used to predict potential human targets of isoquercitrin with specific screening thresholds: bioinformatics analysis tool for molecular mechanism of traditional chinese medicine (BATMAN-TCM) (http://bionet.ncpsb.org.cn/batman-tcm/) (score cutoff > 0.84), SwissTargetPrediction (http://www.swisstargetprediction.ch/) (probability > 0), a high‑throughput experiment- and reference-guided database of traditional Chinese medicine (HERB) (http://herb.ac.cn/), PharmMapper (PM, https://lilab-ecust.cn/pharmmapper/submitfile.html), and TargetNet (http://targetnet.scbdd.com/calcnet/index/).
In total, 4, 22, 15, 287 and 59 putative targets were obtained from BATMAN-TCM, SwissTargetPrediction, HERB, PharmMapper and TargetNet, respectively. All target genes were unified into standard human gene symbols. After data integration and manual deduplication, a total of 347 non-redundant targets of isoquercitrin were acquired for subsequent bioinformatics analysis.*”

**Location of the changes:** Row 187-199.

Lines 161-162: please add vectors used.

**Response:** We apologize for the insufficient information provided in the original description of the lentiviral vectors used for AKT1 overexpression. As suggested, we have now clarified the specific constructs, namely the pLVX-puro empty control vector and the pLVX-puro-AKT1 overexpression lentivirus, to ensure full transparency and reproducibility of the experiment.

Revised sentence added to Lentiviral Transduction:“*The pLVX-puro empty control vector and pLVX-puro-AKT1 overexpression lentivirus were synthesized by Shanghai Jima Pharmaceutical Technology Co., Ltd.*”

**Location of the changes:** Row 250-252.

Line 165: please add concentration of polybrene.

**Response:** We apologize for the omission of the Polybrene concentration in the original manuscript. As requested, we have supplemented the working concentration of Polybrene used during lentiviral transduction, with a final concentration of 5 μg/mL.

Revised sentence added to Lentiviral Transduction:“*For infection, the experimental group was treated with 500 μL fresh complete medium containing lentivirus and Polybrene at a final concentration of 5 μg/mL, while control groups received equal volumes of empty vector lentivirus suspension or uninfected complete medium.*”

**Location of the changes:** Row 254-257.

Line 172: please add post hoc test used for multiple comparison.

**Response:** We appreciate the reviewer’s reminder. We have supplemented the specific post hoc test in the statistical analysis section. In this study, one-way ANOVA was followed by Tukey’s post hoc test for multiple comparisons, which is unified with the description in all figure legends.

Revised sentence added to Statistical Analysis:“*One-way analysis of variance (ANOVA) combined with Tukey’s post hoc test was applied for multiple group comparisons using GraphPad Prism 9.5.0 for Windows (GraphPad Software, Inc., San Diego, CA, USA; https://www.graphpad.com); significance was defined as *P < 0.05 and **P < 0.01.*”

**Location of the changes:** Row 262-265.

Line 173: Please ensure that graphpad software is properly cited

**Response:** We apologize for the incomplete citation of GraphPad Prism. Following the formatting requirements of PLOS ONE and the official citation guidelines of the software, we have supplemented the full version, operating system, developer, location and official website of GraphPad Prism.

Revised sentence added to Statistical Analysis:“*One-way analysis of variance (ANOVA) combined with Tukey’s post hoc test was applied for multiple group comparisons using GraphPad Prism 9.5.0 for Windows (GraphPad Software, Inc., San Diego, CA, USA; https://www.graphpad.com); significance was defined as *P < 0.05 and **P < 0.01.*”

**Location of the changes:** Row 262-265.

Results

Lines 177-184: this part should be moved to the introduction.

**Response:** Thank you for your valuable suggestion. We have moved the background introduction of isoquercitrin from the current section to the Introduction part, where it provides a more logical and appropriate context for the study.

Revised sentence:“*IQ also known as quercetin-3-O-glucoside, is an extensively distributed natural flavonol glycoside that accumulates in the roots, stems, leaves, and fruits of diverse plant species—including Mangifera indica, Rheum nobile, Annona squamosa, and Camellia sinensis [26,27].*”

**Location of the changes:** Row 56-59.

Line 179: Scientific names (binomial nomenclature) must be italicized.

**Response:** We appreciate the reviewer’s reminder regarding binomial nomenclature formatting. We have italicized the plant scientific names as required. Furthermore, we have conducted a full-manuscript inspection and unified the italic format following the journal’s requirements and standard biological nomenclature:

**Plant Latin binomial names**: All botanical scientific names throughout the text are italicized uniformly, *Mangifera indica, Rheum nobile, Annona squamosa* and *Camellia sinensis.*

**Gene symbols**: All gene symbols including *AKT1, SOX9, COL2, ACAN, IL-6, IL-1β, TNF-α, OPA1, MFN1, MFN2, FIS1, DRP1, TNF, ALB and CASP3* are italicized in contexts referring to genes, mRNA expression, gene targets, bioinformatics analysis, gene overexpression and genetic regulation.

**Protein names**: Corresponding protein products detected by Western blot and described in protein functional analysis remain in regular font to distinguish genes from proteins clearly.

Revised sentence:“*IQ also known as quercetin-3-O-glucoside, is an extensively distributed natural flavonol glycoside that accumulates in the roots, stems, leaves, and fruits of diverse plant species—including Mangifera indica, Rheum nobile, Annona squamosa, and Camellia sinensis [26,27].*”

**Location of the changes:** Row 56-59.

Fig 1C: authors should calculate IC50.

**Response:** Thank you for your valuable comment. We have calculated the IC₅₀ of isoquercitrin on chondrocytes using a four-parameter logistic regression model. The IC₅₀ value is 95.05 μM (95% CI: 77.63–629.2 μM). We have added the fitted dose-response curve to **Fig S1** and included the IC₅₀ value in both the main text and figure legend. This result confirms that the working concentration of 40 μM selected in our study is well below the cytotoxic threshold, validating the rationality of our experimental design.

Revised sentence added to Statistical Analysis:“*We further calculated the half-maximal inhibitory concentration (IC₅₀) of IQ using a four-parameter logistic model, and the value was 95.05 μM (95% CI: 77.63–629.2 μM), as presented in Fig S1. Given that 40 μM was well below the cytotoxic IC₅₀, this concentration was selected for all subsequent experiments.*”

**Fig. S1.** Cell viability of *C28/I2* chondrocytes treated with gradient concentrations of IQ (0–160 μM). The dose-response curve was fitted using a four-parameter logistic model, and the IC₅₀ value was determined to be 95.05 μM (95% CI: 77.63–629.2 μM).

**Location of the changes:** Fig S1; Row 284-287.

Figure 1D: statistical analysis is unclear. It is not clear which experimental conditions are being compared. Please include statistical significance indicators (e.g., bars with asterisks) to clearly show the comparisons between groups. Please check all the figures.

Figures 2,3, 6, and 7: please correct “beta-action” to “beta-actin”.

**Response:** Thank you very much for your careful review and valuable suggestions. We apologize for the unclear presentation of the statistical comparisons in our original figures. We have carefully revised all figures and their corresponding legends based on your comments, as follows:

1. **Clarification of statistical comparisons**: We have re-analyzed all data and added clear statistical significance indicators to the graphs to explicitly show which groups are being compared. For example, in **Fig 2**, we now explicitly state:" *Data are mean ± SD (n = 3 independent experiments). *p < 0.05, **p < 0.01, ***p < 0.001 vs. Con group; #p < 0.05, ##p < 0.01, ###p < 0.001 vs. IL-1β group. One-way ANOVA with Tukey’s post hoc test was used for multiple comparisons.*”

All other figures have been updated similarly, with revised legends that clearly define the meaning of each symbol and the comparisons indicated by the connecting bars.

2. **Correction of typographical errors:** As suggested, we have corrected the typo “beta-action” to “β-actin” in **Figs 2, 3, 6,** and **7**, as well as in all relevant figure legends and supplementary data.

We believe these revisions have significantly improved the clarity and readability of our results.

**Fig 2. IQ regulates catabolic, anabolic, and inflammatory protein expression in IL-1β-stimulated chondrocytes. A.** Representative Western blot bands of catabolic enzymes (MMP-1, MMP-13). **B-C.** Quantitative analysis of MMP-1 and MMP-13 protein expression based on Western blot results. **D.** Representative Western blot bands of anabolic matrix markers (SOX9, COL2, ACAN) **E-G.** Quantitative analysis of SOX9, COL2, and ACAN protein expression based on Western blot results. **H.** Representative Western blot bands of pro-inflammatory cytokines (IL-6, IL-1β, TNF-α); β-actin serves as the loading control. **I-K.** Quantitative analysis of IL-6, IL-1β, and TNF-α protein expression based on Western blot results. Data are mean ± SD (n = 3 independent experiments). *p < 0.05, **p < 0.01, ***p < 0.001 vs. Con group; #p < 0.05, ##p < 0.01, ###p < 0.001 vs. IL-1β group. One-way ANOVA with Tukey’s post hoc test was used for multiple comparisons.

**Location of the changes:** Figs 1, 2, 3, 6, and 7, Figure legends of Figs 1, 2, 3, 6, and 7.

Line 319: please add a figure demonstrating the overexpression of AKT1.

**Response:** As suggested, we have now added both Western blot and qRT-PCR data to verify AKT1 overexpression efficiency in chondrocytes, and integrated these results into **Fig 6** and **Fig S7**:

1. In the revised **Fig 6**: We have added AKT1 protein expression data as a new sub-panel (**Fig 6A**). Quantitative analysis confirmed that, compared with the Control, IL-1β, and IL-1β+IQ groups, AKT1 protein level was significantly upregulated in the IL-1β+IQ+AKT1 overexpression group, verifying the successful construction of AKT1-overexpressing cell lines. This new result is directly linked to the subsequent functional assays in the same figure, providing clear evidence for the validity of our rescue experiments.
2. In **Fig S7**: We further confirmed AKT1 overexpression at the mRNA level by qRT-PCR. Consistent with the Western blot results, AKT1 mRNA expression was significantly increased in the IL-1β+IQ+AKT1 group compared with the other three groups, confirming that the lentiviral transfection system worked effectively.

Revised sentence added to Results:“*First, we confirmed the efficiency of lentivirus-mediated AKT1 overexpression: both Western blot (Figs 6 A and B) and qRT-PCR (Fig S7A) results showed that AKT1 protein and mRNA levels were significantly upregulated in the IL-1β+IQ+AKT1 group compared with the Con, IL-1β, and IL-1β+IQ groups, while endogenous AKT1 expression was not significantly altered by IL-1β stimulation or IQ treatment alone.*”

**Fig 6. *AKT1* overexpression abrogates IQ-mediated regulation of chondrocyte catabolism, anabolism, and inflammation.** (**A**) Representative Western blot bands of catabolic enzymes (AKT1, MMP-1, MMP-13). (**B-D**) Quantitative analysis of AKT1, MMP-1 and MMP-13 protein expression based on Western blot results. (**E-G**) Quantitative analysis of SOX9, COL2, and ACAN protein expression based on Western blot results. (**H**) Representative Western blot bands of anabolic matrix markers (SOX9, COL2, ACAN). (**I-K**) Quantitative analysis of IL-6, IL-1β, and TNF-α protein expression based on Western blot results. (**L**) Representative Western blot bands of pro-inflammatory cytokines (IL-6, IL-1β, TNF-α). Data are mean ± SD (n = 3 independent experiments). *p < 0.05, **p < 0.01, ***p < 0.001, ****p < 0.0001 vs. Con group; #p < 0.05, ##p < 0.01, ###p < 0.001, ####p < 0.0001, as indicated by the horizontal line connecting the two groups being compared. One-way ANOVA with Tukey’s post hoc test was used for multiple comparisons.

**Fig. S7**. The qRT-PCR analysis of catabolic enzyme mRNA levels in *C28/I2* chondrocytes: (**A**) *AKT1*, (**B**) *MMP-1* and (**C**) *MMP-13*. Data are mean ± SD (n = 3 independent experiments), with **p < 0.01, ****p < 0.0001 vs. Con group; ####p < 0.0001 as indicated by the horizontal line connecting the two groups being compared. One-way analysis of variance (ANOVA) with Tukey’s post hoc test was used for multiple comparisons.

**Location of the changes:** Fig 6, Fig. S7; Row 451-456.

Why did authors evaluate cytokine protein expression in cells instead of in supernatant?

**Response:** Thank you for this constructive comment. Your suggestion is very helpful for us to further improve and enrich the mechanistic evidence of this study.

Our research primarily aims to explore the intracellular regulatory network between IQ and AKT1 in chondrocytes. Accordingly, we adopted whole-cell lysates for Western blot to detect pro-inflammatory cytokines, which directly reflects the endogenous protein synthesis levels of IL-6, IL-1β, and TNF-α (**Figs 2H-K** and **Figs 6I-L Figs S4A-C**). Combined with qRT-PCR analysis (**Figs S4A-C** and **Figs S9A-C**), our results clearly demonstrate that IQ suppresses both the transcription and translation of pro-inflammatory genes via AKT1, thereby alleviating chondrocyte inflammation. Collectively, our current data sufficiently support that IQ inhibits chondrocyte inflammation via AKT1.

We acknowledge that these cytokines can be secreted into the extracellular space, and that measuring intracellular protein levels provides a direct indicator of the inflammatory state of chondrocytes themselves. Nonetheless, we fully agree that detecting secreted cytokines in the cell supernatant would better capture the paracrine inflammatory effects of chondrocytes within the microenvironment. To address this, in our follow-up studies, we plan to perform ELISA assays to quantify the extracellular secretion of IL-6, IL-1β, and TNF-α.

Revised sentence added to Results:“*Here we analyzed intracellular cytokine expression to reflect inflammatory gene synthesis, and related secretory function will be explored in our future research.*”

**Location of the changes:** Row 469-470.

The Discussion section should be further developed and deepened. Currently, it tends to repeat the description of the results rather than providing critical interpretation and contextualization.

**Response:** Thank you for your insightful and constructive comments on our manuscript. In response to your concern that the Discussion section was overly descriptive and tended to repeat the results rather than providing critical interpretation and contextualization, we have undertaken a thorough revision. Specifically, we have made the following major changes:

1. Shifted from description to interpretation: Instead of restating experimental findings, we now focus on explaining the biological significance of our results. For example, we discuss why the triple mechanism of IQ (restoring proliferation arrest, mitigating inflammation, and normalizing mitochondrial dynamics) represents a coordinated therapeutic strategy rather than isolated effects.
2. Provided mechanistic depth: We elaborated on the signaling network downstream of AKT1, including its regulation of DRP1 phosphorylation (Ser616).
3. Contextualized findings within the field: We now compare our results with existing literature on quercetin/isoquercitrin in OA and other diseases, highlighting novel aspects.
4. Acknowledged limitations and future directions: We have added a paragraph explicitly discussing the multi-tissue nature of OA and the need for future studies in ex vivo joint explant models or in vivo OA models. We also mention the need to directly measure ROS and examine Nrf2 pathway activation in IQ-treated chondrocytes.

We believe these revisions fully address your concern and have transformed the Discussion from a repetitive summary into a critical, forward-looking interpretation that strengthens the manuscript's scientific impact.

Revised sentence added to Results: “*OA is increasingly recognized as a whole-joint disease, involving not only articular cartilage but also the synovium, subchondral bone, ligaments, and periarticular muscles [39,40]. The pathological remodeling of these tissues—synovial inflammation, osteophyte formation, subchondral bone sclerosis, and ligamentous laxity—collectively drives pain and functional decline [41]. Our study focuses on chondrocytes as a central cellular target, but we acknowledge that effective disease-modifying OA therapies must ultimately address this multi-tissue pathology. While IQ demonstrates pronounced chondroprotective effects in vitro, its potential impact on synovial fibroblasts (which contribute to inflammatory cytokine production) or osteoclast/osteoblast activity in subchondral bone remains unexplored. Future studies should evaluate IQ’s effects in ex vivo joint explant models or in vivo OA models that recapitulate this complex tissue crosstalk.
The present work reveals that IQ restores chondrocyte functional homeostasis through a triple mechanism: (1) reversing IL-1β-induced proliferation arrest and shifting metabolism toward anabolism, (2) mitigating pro-inflammatory responses, and (3) normalizing mitochondrial dynamics. Critically, we identify AKT1 as a central node mediating these effects. Molecular docking predicts high-affinity binding of IQ to AKT1 (-10.4 kcal/mol), and AKT1 overexpression abrogates IQ’s protective benefits, confirming its necessity. However, the precise mechanism by which AKT1 coordinates mitochondrial dynamics deserves further elaboration. AKT1 bidirectionally regulates DRP1 phosphorylation: Ser616 modification promotes mitochondrial fission, while Ser637 modification restrains it, with the net effect dependent on cellular pathological status [42]. In our IL-1β-stimulated OA model, chronic inflammation triggers sustained aberrant AKT1 hyperactivation, upregulating DRP1 Ser616 phosphorylation and driving excessive pathological mitochondrial fission. IQ binds AKT1 to function as an allosteric modulator that dampens its pathological overactivation to balance mitochondrial fission and fusion. This molecular pathway accounts for the recovered mitochondrial membrane potential observed after IQ treatment. The excess unbound AKT1 from lentiviral overexpression exceeds IQ’s binding saturation limit, retaining pathological hyperactivity and eliminating IQ’s protective capacity, further confirming that IQ acts to normalize overactive AKT1.
Oxidative stress is a hallmark of OA chondrocytes, driven by mitochondrial dysfunction and nicotinamide adenine dinucleotide phosphate (NADPH) oxidase (NOX) activation [43,44]. We did not directly measure ROS levels in this study, but the literature strongly suggests that IQ is a potent antioxidant. Quercetin, the aglycone of IQ, directly scavenges ROS and upregulates nuclear factor erythroid 2-related factor 2/ antioxidant response element (Nrf2/ARE) pathway, while IQ itself reduces ROS in other cell types (e.g., hepatocytes, neurons) [25,45,46]. Given that IL-1β elevates ROS via NOX4 and mitochondrial electron transport chain leakage, IQ’s mitochondrial-stabilizing effect likely attenuates ROS production indirectly [46]. Furthermore, IQ binds AKT1 as an allosteric buffer to dampen pathological AKT1 hyperactivity and reset AKT1 signaling to physiological levels. Therefore, IQ may suppress ROS through both direct scavenging and AKT1-mediated Nrf2 activation. Future experiments should quantify intracellular ROS (e.g., 2′,7′-Dichlorodihydrofluorescein diacetate (DCFH-DA) staining) and examine Nrf2 pathway activation (e.g., nuclear translocation, ARE-luciferase reporter) in IQ-treated chondrocytes.
In conclusion, this study establishes IQ as a promising candidate for OA intervention by targeting AKT1 to restore chondrocyte anabolic-catabolic balance, suppress inflammation, and stabilize mitochondrial dynamics. The multi-target nature of IQ—acting on proliferation, metabolism, inflammation, and mitochondria—offers advantages over single-target agents. However, translation to clinical application requires several steps: (1) evaluation of IQ bioavailability and joint pharmacokinetics, (2) assessment of efficacy in post-traumatic and age-related OA animal models (e.g., destabilization of the medial meniscus (DMM) or monosodium iodoacetate (MIA)-induced rats), and (3) investigation of potential off-target effects on other joint tissues. Our findings provide a mechanistic framework linking AKT1 to chondroprotection, opening avenues for developing AKT1-targeted DMOADs.*”

**Location of the changes:** Row 525-575

Lines 350-352: this part should be improved. Please explain that OA is a whole joint disease involving all joint tissue and not only cartilage.

**Response:** Thank you for your insightful comment. We agree with the reviewer’s suggestion and have revised Lines 350-352 accordingly to clearly emphasize that OA is a whole joint disease involving all joint tissues, not only cartilage.

Revised sentence added to Discussion: “*OA is increasingly recognized as a whole-joint disease, involving not only articular cartilage but also the synovium, subchondral bone, ligaments, and periarticular muscles [39,40]. The pathological remodeling of these tissues—synovial inflammation, osteophyte formation, subchondral bone sclerosis, and ligamentous laxity—collectively drives pain and functional decline [41]. Our study focuses on chondrocytes as a central cellular target, but we acknowledge that effective disease-modifying OA therapies must ultimately address this multi-tissue pathology.*”

**Location of the changes:** Row 525-530.

Lines 353-356, line 357: please add specific references.

Lines 360-361: please check and correct the references adding references on OA instead of cancer.

Lines 361-363: please check and correct the references.

Lines 383-386: please delete the references.

**Response:** Thank you for your careful reading and valuable suggestions regarding our manuscript. We have taken your comments into full consideration.

Regarding the references, we have deleted all the original references from the previous version. In the revised manuscript, we have added new, specific references that are directly relevant to the statements made in the manuscript. These newly added references are from the latest research in the field and are accurate and authoritative, providing strong support for the claims made in the text. We hope these revisions meet your expectations.

Revised References:

[39] Huang X, Wang Z, Wang H, Chen D, Tong L. Novel strategies for the treatment of osteoarthritis based on biomaterials and critical molecular signaling. J Mater Sci Technol. 2023 Jun 20;149:42–55. doi:10.1016/j.jmst.2022.11.027
[40] Umar M, Wang Z, Li J, Li Z, Li G, Tong L, et al. Novel therapeutic strategies for osteoarthritis: from mechanistic insights to precision medicine. Bone Res. 2026 Jun 11;14(1):64. doi:10.1038/s41413-026-00540-6
[41] Liao L, Zhang S, Zhao L, Chang X, Han L, Huang J, et al. Acute synovitis after trauma precedes and is associated with osteoarthritis onset and progression. Int J Biol Sci. 2020;16(6):970–80. doi:10.7150/ijbs.39015
[42] Adhikary A, Mukherjee A, Banerjee R, Nagotu S. DRP1: At the crossroads of dysregulated mitochondrial dynamics and altered cell signaling in cancer cells. ACS Omega. 2023 Dec 5;8(48):45208–23. doi:10.1021/acsomega.3c06547
[43] Liu L, Luo P, Yang M, Wang J, Hou W, Xu P. The role of oxidative stress in the development of knee osteoarthritis: A comprehensive research review. Front Mol Biosci. 2022 Sep 20;9:1001212. doi:10.3389/fmolb.2022.1001212
[44] Han J, Park D, Park JY, Han S. Inhibition of NADPH oxidases prevents the development of osteoarthritis. Antioxidants. 2022 Nov 27;11(12):2346. doi:10.3390/antiox11122346
[45] Treml J, Leláková V, Šmejkal K, Paulíčková T, Labuda Š, Granica S, et al. Antioxidant activity of selected stilbenoid derivatives in a cellular model system. Biomolecules. 2019 Sep 9;9(9):468. doi:10.3390/biom9090468
[46] Dai Y, Zhang H, Zhang J, Yan M. Isoquercetin attenuates oxidative stress and neuronal apoptosis after ischemia/reperfusion injury via Nrf2-mediated inhibition of the NOX4/ROS/NF-κB pathway. Chem Biol Interact. 2018 Mar;284:32–40. doi:10.1016/j.cbi.2018.02.017

**Location of the changes:** Refs 39-46.

Fundings are lacking.

**Response:** Thank you for pointing this out. We have now included the funding information in the separate Financial Disclosure Statement, as required by the journal:

“*The authors received no specific funding for this work.*”

Abbreviations should be consistently used throughout the manuscript, for example OA.

**Response:** Thanks for the constructive suggestion. We have thoroughly reviewed the entire manuscript and unified the usage of all abbreviations. Taking OA as an example, we have defined its full name when it first appears in the text, and only use the abbreviation afterwards without alternating between the full term and shorthand. All abbreviations including OA are applied consistently across all texts, figures and tables. Any non-standard or inconsistent expressions have been revised accordingly.
