# Supplementary material for: Isoquercitrin ameliorates chondrocyte dysfunction in osteoarthritis by targeting AKT1-mediated mitochondrial dynamics regulation
Source: PLoS One. 2026 Sep 17;21(9):e0357989. doi: 10.1371/journal.pone.0357989 (PMC13585277; doi:10.1371/journal.pone.0357989)
Supplement: Peer review history

# Peer review: Decision Letter 0

**DOI:** 10.1371/journal.pone.0357989.r001

**Authors:** David Chau

6 May 2026

Dear Dr. Zhang,

Thank you for submitting your manuscript to PLOS ONE. After careful consideration, we feel that it has merit but does not fully meet PLOS ONE’s publication criteria as it currently stands. Therefore, we invite you to submit a revised version of the manuscript that addresses the points raised during the review process.

Please submit your revised manuscript by Jun 20 2026 11:59PM. If you will need more time than this to complete your revisions, please reply to this message or contact the journal office at plosone@plos.org. When you're ready to submit your revision, log on to https://www.editorialmanager.com/pone/ and select the 'Submissions Needing Revision' folder to locate your manuscript file.

- A letter that responds to each point raised by the academic editor and reviewer(s). You should upload this letter as a separate file labeled 'Response to Reviewers'.
- A marked-up copy of your manuscript that highlights changes made to the original version. You should upload this as a separate file labeled 'Revised Manuscript with Track Changes'.
- An unmarked version of your revised paper without tracked changes. You should upload this as a separate file labeled 'Manuscript'.

If you would like to make changes to your financial disclosure, please include your updated statement in your cover letter. Guidelines for resubmitting your figure files are available below the reviewer comments at the end of this letter.

If applicable, we recommend that you deposit your laboratory protocols in protocols.io to enhance the reproducibility of your results. Protocols.io assigns your protocol its own identifier (DOI) so that it can be cited independently in the future. For instructions see: https://journals.plos.org/plosone/s/submission-guidelines#loc-laboratory-protocols. Additionally, PLOS ONE offers an option for publishing peer-reviewed Lab Protocol articles, which describe protocols hosted on protocols.io. Read more information on sharing protocols at https://plos.org/protocols?utm_medium=editorial-email&utm_source=authorletters&utm_campaign=protocols.

As the corresponding author, your ORCID iD is verified in the submission system and will appear in the published article. PLOS supports the use of ORCID, and we encourage all coauthors to register for an ORCID iD and use it as well. Please encourage your coauthors to verify their ORCID iD within the submission system before final acceptance, as unverified ORCID iDs will not appear in the published article. *Only*  the individual author can complete the verification step; PLOS staff *cannot*  verify ORCID iDs on behalf of authors.

We look forward to receiving your revised manuscript.

Kind regards,

David Chau

Academic Editor

PLOS One

Journal Requirements:

When submitting your revision, we need you to address these additional requirements.

1. Please ensure that your manuscript meets PLOS ONE's style requirements, including those for file naming. The PLOS ONE style templates can be found at

https://journals.plos.org/plosone/s/file?id=wjVg/PLOSOne_formatting_sample_main_body.pdf and https://journals.plos.org/plosone/s/file?id=ba62/PLOSOne_formatting_sample_title_authors_affiliations.pdf

2. PLOS ONE now requires that authors provide the original uncropped and unadjusted images underlying all blot or gel results reported in a submission’s figures or Supporting Information files. This policy and the journal’s other requirements for blot/gel reporting and figure preparation are described in detail at https://journals.plos.org/plosone/s/figures#loc-blot-and-gel-reporting-requirements and https://journals.plos.org/plosone/s/figures#loc-preparing-figures-from-image-files. When you submit your revised manuscript, please ensure that your figures adhere fully to these guidelines and provide the original underlying images for all blot or gel data reported in your submission. See the following link for instructions on providing the original image data: https://journals.plos.org/plosone/s/figures#loc-original-images-for-blots-and-gels.

In your cover letter, please note whether your blot/gel image data are in Supporting Information or posted at a public data repository, provide the repository URL if relevant, and provide specific details as to which raw blot/gel images, if any, are not available. Email us at plosone@plos.org if you have any questions.

3. PLOS requires an ORCID iD for the corresponding author in Editorial Manager on papers submitted after December 6th, 2016. Please ensure that you have an ORCID iD and that it is validated in Editorial Manager. To do this, go to ‘Update my Information’ (in the upper left-hand corner of the main menu), and click on the Fetch/Validate link next to the ORCID field. This will take you to the ORCID site and allow you to create a new iD or authenticate a pre-existing iD in Editorial Manager.

4. Please note that PLOS One has specific guidelines on code sharing for submissions in which author-generated code underpins the findings in the manuscript. In these cases, we expect all author-generated code to be made available without restrictions upon publication of the work. Please review our guidelines at https://journals.plos.org/plosone/s/materials-and-software-sharing#loc-sharing-code and ensure that your code is shared in a way that follows best practice and facilitates reproducibility and reuse.

5. We note that Figure(s) 1, S5 in your submission contain copyrighted images. All PLOS content is published under the Creative Commons Attribution License (CC BY 4.0), which means that the manuscript, images, and Supporting Information files will be freely available online, and any third party is permitted to access, download, copy, distribute, and use these materials in any way, even commercially, with proper attribution. For more information, see our copyright guidelines: http://journals.plos.org/plosone/s/licenses-and-copyright.

We require you to either (1) present written permission from the copyright holder to publish these figures specifically under the CC BY 4.0 license, or (2) remove the figures from your submission:

a. You may seek permission from the original copyright holder of Figure(s) 1, S5 to publish the content specifically under the CC BY 4.0 license.

We recommend that you contact the original copyright holder with the Content Permission Form (http://journals.plos.org/plosone/s/file?id=7c09/content-permission-form.pdf) and the following text:

“I request permission for the open-access journal PLOS ONE to publish XXX under the Creative Commons Attribution License (CCAL) CC BY 4.0 (http://creativecommons.org/licenses/by/4.0/). Please be aware that this license allows unrestricted use and distribution, even commercially, by third parties. Please reply and provide explicit written permission to publish XXX under a CC BY license and complete the attached form.”

Please upload the completed Content Permission Form or other proof of granted permissions as an "Other" file with your submission.

In the figure caption of the copyrighted figure, please include the following text: “Reprinted from [ref] under a CC BY license, with permission from [name of publisher], original copyright [original copyright year].”

b. If you are unable to obtain permission from the original copyright holder to publish these figures under the CC BY 4.0 license or if the copyright holder’s requirements are incompatible with the CC BY 4.0 license, please either i) remove the figure or ii) supply a replacement figure that complies with the CC BY 4.0 license. Please check copyright information on all replacement figures and update the figure caption with source information. If applicable, please specify in the figure caption text when a figure is similar but not identical to the original image and is therefore for illustrative purposes only.

6. If the reviewer comments include a recommendation to cite specific previously published works, please review and evaluate these publications to determine whether they are relevant and should be cited. There is no requirement to cite these works unless the editor has indicated otherwise.

[Note: HTML markup is below. Please do not edit.]

Reviewers' comments:

Reviewer's Responses to Questions

**Comments to the Author**

1. Is the manuscript technically sound, and do the data support the conclusions?

Reviewer #1: Partly

Reviewer #2: Yes

Reviewer #3: Partly

**********

2. Has the statistical analysis been performed appropriately and rigorously?

Reviewer #1: Yes

Reviewer #2: Yes

Reviewer #3: Yes

**********

3. Have the authors made all data underlying the findings in their manuscript fully available?

The PLOS Data policy

Reviewer #1: No

Reviewer #2: Yes

Reviewer #3: Yes

**********

4. Is the manuscript presented in an intelligible fashion and written in standard English?

Reviewer #1: Yes

Reviewer #2: Yes

Reviewer #3: Yes

**********

Reviewer #1: Overall, this is a well-structured study with a clear logical flow from bioinformatic prediction to experimental validation. The combination of molecular dynamics and rescue experiments provides a solid mechanistic foundation. I found this a nice work.

However: it can be improved, especially in the material and methods section, here my suggestions.

please recheck for misspelled words, in particular in the graphs and pictures.

Several critical parameters are missing from the Materials and Methods section

• EdU Staining for Cell Proliferation, line 80: missing details for “click reaction” and for about the microscope

• Western Blotting: % of gels? Were them gradient or not? equivalent protein …how much? Please describe the ”proper conditions” also; blocked with 5% (w/v) skim milk and in what did you dissolved the milk powder? Please add details

You listed primary antibodies but omitted the dilutions for secondary antibodies.

• JC-1 Staining: please add details

• Line 162: “ cells were digested, resuspended, and seeded” would you say “detached”?

just to be clear: "Digested" usually refers to the process of breaking down tissue to get cells. For moving cells from one plate to another, "detached" or "trypsinized" is the standard terminology.

• Fig 1C: Cell viability assay of C28/I2 chondrocytes treated with gradient IQ concentrations (0-160 μM) under IL-1β stimulation (or without??), please add in the caption the concentration for IL1b, please confirm that you obtained 100% viability after exposing chondrocyte to IL1B. How did you decide the 10ng/ml IL1B amount?

I think there is a confusion in data representation: put all the graphs

surviaval for isoquercitine only, surviaval for IQ + IL1B, surviaval for IL1B only

IL1B: error bar is a bit big, how many replicates did you do? They appears like 2 distinct groups.

• Please indicate clearly in the main manuscript that qPCR primers are listed in the supporting material as well as that you normalized per GAPDH.

• Fig 2 e fig 3 e fig 6: B-action….you would say B-actine?

• Graphical abstract: dOgradation.

• In my opinion, qPCR data should be in the main document, to discuss with the editor

Some suggestions to improve discussion or the manuscript in general:

• You link AKT1 to mitochondrial dynamics but don't explain the "how."

• What about enquiring/discussing effects of IQ on ROS?

• Show a co-localization of TOM20 (a mitochondrial marker) and 8-OHdG (a marker of oxidative DNA damage).

• Since your core claim involves mitochondrial dynamics, I suggest (if possible) to add a functional assay like membrane potential or ATP production via cytometry or fluorescence microscopy

PLOS ONE strongly encourages the upload of Original Uncropped Western Blot Images as Supporting Information, please add them, if available.

Authors should contextualize their findings within broader nutraceutical or combinatorial anti-inflammatory strategies, just for example: In Vitro Effects of Low Doses of β-Caryophyllene, Ascorbic Acid and d-Glucosamine on Human Chondrocyte Viability and Inflammation, Mattiuzzo et al 2021

Reviewer #2: The manuscript “Isoquercitrin Ameliorates Chondrocyte Dysfunction in Osteoarthritis by Targeting AKT1-Mediated Mitochondrial Dynamics Regulation” drew my attention because it investigates in detail the mechanisms of isoquercitrin protection for chondrocytes. The topic is relevant for the potential use of isoquercitrin as a therapeutic agent and adds to the evidence of its anti-inflammatory and protective activity.

Throughout the manuscript the word isoquercitrin and its abbreviation IQ are used interchangeably without obeying any apparent rule. Please correct this situation.

Some abbreviations in the text, such as DMM, MIA, OPA and others are not explained. Please explain them.

Materials and methods need to be explained in better detail, particularly in the situations mentioned bellow:

L75: please describe the model of microplate reader used, the wavelength at which measurements were made and the formula employed to calculate viability based on OD.

L82: please describe the model of microscope you used; please explain in detail how you used the fluorescence images to calculate cell proliferation

L97: please explain the ImageJ protocol you used for this analysis.

Tables and figures are descriptive and effective in explaining the results. The conclusion is consistent with the results and arguments detailed in the Discussion section.

Reviewer #3: Several citations appear to be incorrect or mismatched with the statements they are intended to support. A thorough and systematic revision of the reference list is required. The authors should carefully verify all citations, ensure correct matching between references and statements.

Abstract needs to be revised. Methods should be added.

Introduction

Introduction on OA should be improved. It should be explained that OA is a whole joint disease involving all joint tissues. OA is characterized by subchondral bone remodelling), meniscal degeneration, inflammation of the synovial membrane and inflammation and biomechanical changes of the infrapatellar fat pad and ((doi.org/10.3389/fcell.2020.607764, DOI: 10.4081/ejh.2019.2998, DOI: 10.1080/03008207.2025.2502591 etc).

Risk factors for OA should be added.

Lines 29-32: please check the references as they seem to be not appropriate.

Lines 32-34: reference 7 is paper focused on the role of pirfenidone in attenuating synovial fibrosis. Please correct. The same for reference 8.

Lines 35-37: please check the references as they do not appear to support the concept.

Line 37: authors should explain that infrapatellar fat pad and synovial membrane are inflamed and secretes pro-inflammatory cytokines.

Lines 39-40: please provide appropriate references.

Lines 43-44: please provide appropriate references.

Line 46: please define “IQ”.

Lines 46-48: please verify that all references are focused on OA.

Line 50: please define AKT1.

Line 52: please verify that these references are appropriate.

At the end of the introduction, authors should report the aim/s of the study rather that the results.

Methods

Supplier of all substances and kits used should be provided.

Line 75: please add the instrument used.

Line 77:please add seeded cells.

Lines 82 and 111 :please add microscope used.

Line 84:please add buffer and inhibitors used.

Line 85: how did authors quantify the proteins? How many micrograms of protein were loaded? Did authors use SDS-PAGE? Percentage?

Line 86:please specify the membrane used.

Why did authors used skim milk to block the membranes? This is no appropriate when using phosphoantibodies.

Line 95” dilution unspecified” is unclear. Please specify the antibodies and the dilution.

Line 96:please add kit used for the developing.

Line 97: Please ensure that the imagej software is properly cited and referenced.

The primers sequence should be added.

Line 108: how many cells?

The section target collection isoquercitrin should be improved.

Lines 161-162: please add vectors used.

Line 165: please add concentration of polybrene.

Line 172: please add post hoc test used for multiple comparison.

Line 173: : Please ensure that graphpad software is properly cited

Results

Lines 177-184: this part should be moved to the introduction.

Line 179: Scientific names (binomial nomenclature) must be italicized.

Figure 1C: authors should calculate IC50.

Figure 1D: statistical analysis is unclear. It is not clear which experimental conditions are being compared. Please include statistical significance indicators (e.g., bars with asterisks) to clearly show the comparisons between groups. Please check all the figures.

Figures 2,3, 6, and 7 : please correct “beta-action” to “beta-actin”.

Line 319: please add a figure demonstrating the overexpression of AKT1.

Why did authors evaluate cytokine protein expression in cells instead of in supernatant?

The Discussion section should be further developed and deepened. Currently, it tends to repeat the description of the results rather than providing critical interpretation and contextualization.

Lines 350-352: this part should be improved. Please explain that OA is a whole joint disease involving all joint tissue and not only cartilage.

Lines 353-356, line 357: please add specific references.

Lines 360-361: please check and correct the references adding references on OA instead of cancer.

Lines 361-363: please check and correct the references.

Lines 383-386: please delete the references.

Fundings are lacking.

Abbreviations should be consistently used throughout the manuscript, for example OA.

**********

what does this mean?). If published, this will include your full peer review and any attached files.

If you choose “no”, your identity will remain anonymous but your review may still be made public.

**Do you want your identity to be public for this peer review?**  For information about this choice, including consent withdrawal, please see our Privacy Policy

Reviewer #1: No

Reviewer #2: No

Reviewer #3: No

**********

[NOTE: If reviewer comments were submitted as an attachment file, they will be attached to this email and accessible via the submission site. Please log into your account, locate the manuscript record, and check for the action link "View Attachments". If this link does not appear, there are no attachment files.]

To ensure your figures meet our technical requirements, please review our figure guidelines: https://journals.plos.org/plosone/s/figures

You may also use PLOS’s free figure tool, NAAS, to help you prepare publication quality figures: https://journals.plos.org/plosone/s/figures#loc-tools-for-figure-preparation.

NAAS will assess whether your figures meet our technical requirements by comparing each figure against our figure specifications.

# Author comment: Author response to Decision Letter 1

**DOI:** 10.1371/journal.pone.0357989.r002

2 Jul 2026

Reviewer #1: Overall, this is a well-structured study with a clear logical flow from bioinformatic prediction to experimental validation. The combination of molecular dynamics and rescue experiments provides a solid mechanistic foundation. I found this a nice work. However: it can be improved, especially in the material and methods section, here my suggestions. please recheck for misspelled words, in particular in the graphs and pictures.

General response: We thank Reviewer #1 for his/her positive evaluation of our work on isoquercitrin ameliorating chondrocyte dysfunction in osteoarthritis by targeting AKT1-mediated mitochondrial dynamics, as well as the highly constructive and meticulous comments that have greatly helped us improve the manuscript. In response to the constructive comments, we have revised the manuscript thoroughly. We carefully checked and corrected all spelling errors throughout the manuscript, particularly in figures and legends. In addition, we have further refined and supplemented the Materials and Methods section, adding detailed descriptions of experimental procedures and parameters as requested. Below, we provide our detailed point-by-point responses to all comments.

Several critical parameters are missing from the Materials and Methods section

• EdU Staining for Cell Proliferation, line 80: missing details for “click reaction” and for about the microscope.

Response: We thank the reviewer for pointing out these missing details in the Materials and Methods section. We have supplemented the detailed experimental procedures of EdU staining in the revised manuscript. Specifically, we added a complete description of the click reaction and relevant parameters of the fluorescence microscope. In addition, we have also supplemented the full information of the analysis software including version number and official website in accordance with the journal requirements.

Location of the changes: Row 118-124.

• Western Blotting: % of gels? Were them gradient or not? equivalent protein …how much? Please describe the” proper conditions” also; blocked with 5% (w/v) skim milk and in what did you dissolved the milk powder? Please add details You listed primary antibodies but omitted the dilutions for secondary antibodies.

Response: We apologize for the lack of detailed experimental parameters in the original manuscript, which compromised the rigor of our work. We have now fully revised and supplemented the Western blotting section as requested, with detailed additions listed as follows:

1. We clearly stated that 6% or 12% non-gradient SDS-PAGE gels were used for protein separation, and the loading quantity of each protein sample was set at 30 μg.

2. We revised the blocking solution: instead of skim milk, we used 5% (w/v) bovine serum albumin (BSA) diluted in TBST for membrane blocking. BSA was chosen because skim milk contains endogenous immunoglobulins, which may cause non-specific binding and interfere with the detection of target proteins.

3. We supplemented the dilution ratio (1:5000) for HRP-conjugated secondary antibodies.

4. We added complete information of the image analysis software ImageJ, including its developer, official website and corresponding reference, in accordance with journal requirements.

All optimized experimental details have been incorporated into the revised manuscript to guarantee experimental repeatability.

Location of the changes: Row 126-149.

• JC-1 Staining: please add details

Response: We have now supplemented all critical experimental parameters in the revised manuscript, including the kit information, working solution preparation, incubation conditions, washing steps, fluorescence detection settings, and imaging system specifications, as shown below:

“The working solution was prepared by diluting the JC-1 stock (200×) with ultrapure water and JC-1 staining buffer (5×) according to the standard protocol. Post-treatment, cells were rinsed with pre-warmed PBS, incubated with JC-1 working solution at 37 °C for 20 min in the dark, then washed twice with ice-cold JC-1 staining buffer (1×). Fluorescence signals were visualized using an inverted fluorescence microscope (Leica DMIL LED, Leica Microsystems, Wetzlar, Germany), with red fluorescence (JC-1 aggregates) and green fluorescence (JC-1 monomers) detected at excitation/emission wavelengths of 525/590 nm and 490/530 nm, respectively.”

Location of the changes: Row 165-172

• Line 162: “cells were digested, resuspended, and seeded” would you say “detached”?

just to be clear: "Digested" usually refers to the process of breaking down tissue to get cells. For moving cells from one plate to another, "detached" or "trypsinized" is the standard terminology.

Response: Thank you for this helpful comment on terminology clarity. We agree that “digested” is more commonly used for dissociating cells from tissues, whereas “detached” or “trypsinized” is the standard term for releasing adherent cells from culture plates. We have revised the manuscript accordingly, replacing the phrase “cells were digested, resuspended, and seeded” with “cells were detached with trypsin-EDTA, resuspended, and seeded”. This correction improves the precision and clarity of the experimental description.

Location of the changes: Row 250, 251.

• Fig 1C: Cell viability assay of C28/I2 chondrocytes treated with gradient IQ concentrations (0-160 μM) under IL-1β stimulation (or without??), please add in the caption the concentration for IL-1β, please confirm that you obtained 100% viability after exposing chondrocyte to IL-1β. How did you decide the 10ng/ml IL-1β amount?

I think there is a confusion in data representation: put all the graphs surviaval for isoquercitine only, surviaval for IQ + IL-1β, surviaval for IL-1β only. IL-1β: error bar is a bit big, how many replicates did you do? They appears like 2 distinct groups.

Response: We greatly appreciate the reviewer’s valuable comments and suggestions. We have addressed all points one by one in detail as follows:

1. Supplementation of IL-1β concentration and selection rationale

We have added relevant descriptions in the Results section. C28/I2 chondrocytes were treated with 10 ng/mL IL-1β to establish an OA-mimetic in vitro model. This concentration is widely validated and commonly adopted in published related studies (refs., J Extracell Vesicles. 2021, 10, e12160; Nat Commun. 2020, 11, 3427), so we directly applied it without additional dose optimization. Revised sentence added to Results:“To explore the influence of IQ on the proliferation of OA-like chondrocytes, C28/I2 cells were stimulated with 10 ng/mL IL-1β (a standard concentration used in prior studies) to construct an in vitro model [35,36].

2. Explanation for cell viability assay design and data classification

Prior to functional experiments, we performed CCK-8 assays to screen the working concentration of IQ. This assay was conducted in chondrocytes without IL-1β stimulation. We further calculated the IC₅₀ of IQ using a four-parameter logistic model, and the value was 95.05 μM (95% CI: 77.63–629.2 μM), as shown in Fig S1. Since 40 μM was well below the cytotoxic concentration, we selected 40 μM IQ for all subsequent experiments. We did not perform separate CCK-8 tests for the IL-1β-only group and IQ + IL-1β group. The 10 ng/mL IL-1β adopted here is a mature and well-recognized concentration in chondrocyte research, and its influence on cell viability has been fully verified in previous work. We apologize that we did not optimize IQ concentration under IL-1β treatment via cell viability assays. However, subsequent functional experiments fully demonstrated that 40 μM IQ exerted favorable protective effects against IL-1β-induced chondrocyte damage. Given that 40 μM IQ was used uniformly across the entire study, re-running full concentration-gradient tests under IL-1β stimulation would require a substantial amount of extra work. We hereby promise to explore the optimal IQ concentration in the presence of IL-1β in our future investigations. Revised sentence added to Results:“Prior to functional assays, we assessed the cytotoxicity of IQ in C28/I2 chondrocytes without 10 ng/mL IL-1β stimulation using the CCK-8 assay, so as to determine the optimal working concentration for subsequent experiments.

We further calculated the half-maximal inhibitory concentration (IC₅₀) of IQ using a four-parameter logistic model, and the value was 95.05 μM (95% CI: 77.63–629.2 μM), as presented in Fig S1. Given that 40 μM was well below the cytotoxic IC₅₀, this concentration was selected for all subsequent experiments.”

Revised the legend for Fig 1C: “Cell viability of C28/I2 chondrocytes treated with gradient concentrations of IQ without IL-1β stimulation. **p < 0.01, ****p < 0.0001 vs. 0 μM group.”

3. Revision of Fig 1D and explanation for error bars and replicates

We sincerely apologize for the large error bars and discrete data distribution in the original Fig 1D. This issue was caused by inappropriate statistical processing of the original datasets, rather than insufficient biological replicates. We have re-analyzed data from independent biological replicates with correct statistical methods and updated Fig 1D accordingly. The revised graph shows obviously reduced variability, and the statistical significance of IQ’s protective effect remains robust. The exact number of replicates has also been clearly marked in the updated figure legend.

Location of the changes: Row 275-278; 282-284; 286-289; 301-304; Fig 1D; Fig. S1.

• Please indicate clearly in the main manuscript that qPCR primers are listed in the supporting material as well as that you normalized per GAPDH.

Response: We sincerely apologize for this oversight in the original submission. We have now clearly stated in the main manuscript that all qPCR gene expression data were normalized to the housekeeping gene GAPDH, and the complete sequences of all primers used in this study are explicitly listed in Table 1 in the Supporting Information. These additions ensure full transparency and reproducibility of the qPCR experiments, as requested.

Location of the changes: Row 158-161.

• Fig 2 e fig 3 e fig 6: β-action…. you would say β-actine?

Response: Thank you very much for pointing out this typographical error. We sincerely apologize for the confusion caused by the misspelling of “β-actin” in the figure labels. We have now carefully reviewed and corrected all instances of “β-action” to the correct term “β-actin” in Figs 2, 3, 6 and 7 as well as in the corresponding figure legends and main text. All corrections are clearly marked in the revised manuscript.

Location of the changes: Figs 2, 3, 6 and 7.

• Graphical abstract: dOgradation.

Response: We sincerely apologize for this spelling error, which was caused by our carelessness during the final check. We greatly appreciate the reviewer’s careful observation and helpful feedback. We have corrected “dOgradation” to the correct spelling “degradation” in the revised graphical abstract. The corrected graphical abstract has been updated in the revised manuscript.

• In my opinion, qPCR data should be in the main document, to discuss with the editor

Response: We fully respect the reviewer’s opinion and agree that including the key qPCR data in the main manuscript can strengthen the presentation of our findings and facilitate discussion. To address this, we have moved the core qPCR results into the main document, where they are now presented alongside the corresponding protein expression data.

Location of the changes: Row 158-161.

Some suggestions to improve discussion or the manuscript in general:

• You link AKT1 to mitochondrial dynamics but don't explain the "how."

Response: Thank you for this critical comment. We have comprehensively elaborated the regulatory mechanism between AKT1 and mitochondrial dynamics in the revised Discussion section. In this study, both molecular docking and AKT1 overexpression experiments verified that AKT1 is an essential molecular target of IQ. Beyond its roles in apoptosis and mitochondrial biogenesis via mTORC1/PGC-1α (refs., Aging Dis. 2022, 13, 157; Front Med. 2025, 12, 1635219), AKT1 controls mitochondrial fission through site-specific phosphorylation of DRP1 (refs., ACS Omega. 2023, 8, 45208–23). In IL‑1β-induced OA chondrocytes, chronic inflammatory stimulation triggers sustained, pathological hyperactivation of AKT1 (refs., Bone Res. 2019, 7, 23). In our OA model, IL-1β activates AKT1 to increase DRP1 Ser616 phosphorylation and drive mitochondrial fission. IQ inhibits AKT1 activity, and restores the balance of mitochondrial fusion and fission.

Location of the changes: Row 548-564.

• What about enquiring/discussing effects of IQ on ROS?

Response: We appreciate the reviewer’s important comment. We have fully discussed the potential relationship between IQ and oxidative stress in the revised Discussion section. Oxidative stress driven by mitochondrial impairment and NOX activity is prominent in OA chondrocytes (refs., Front Mol Biosci. 2022, 9, 1001212; Antioxidants. 2022, 11, 2346). While ROS was not directly measured in this study, published studies have demonstrated the antioxidant property of IQ. Its aglycone quercetin can scavenge ROS and activate the Nrf2/ARE pathway, and IQ has been confirmed to lower ROS levels in hepatocytes and neurons (refs., Int J Mol Sci. 2025, 26, 2717; Biomolecules. 2019, 9, 468; Chem Biol Interact. 2018, 284, 32–40).

IL-1β triggers ROS production through NOX4 and damaged mitochondrial electron transport chain. The mitochondrial protective effect of IQ can indirectly restrain ROS release. Meanwhile, AKT1 modulates Nrf2 signaling and antioxidant gene expression (refs., Antioxidants. 2021, 10, 554). We therefore infer that IQ relieves oxidative stress via direct ROS clearance and AKT1-mediated Nrf2 activation. We have also put forward corresponding experimental plans, including DCFH-DA staining and Nrf2 pathway detection, for further validation in future work.

Location of the changes: Row 565-577.

• Show a co-localization of TOM20 (a mitochondrial marker) and 8-OHdG (a marker of oxidative DNA damage).

Response: We thank the reviewer for this valuable suggestion, which helps strengthen the mechanistic link between mitochondrial dysfunction and oxidative DNA damage. We have now added immunofluorescence co-localization staining of TOM20 (mitochondrial marker) and 8-OHdG (oxidative DNA damage marker) to directly visualize this association, as shown in the revised Fig S12.

Briefly, IL-1β stimulation induced severe mitochondrial fragmentation and significantly increased 8-OHdG fluorescence intensity, with prominent co-localization of TOM20 and 8-OHdG, indicating robust mitochondrial oxidative DNA damage. IQ treatment notably restored mitochondrial morphology and reduced 8-OHdG accumulation, accompanied by decreased co-localization between TOM20 and 8-OHdG. Importantly, AKT1 overexpression completely abrogated the protective effects of IQ, re-inducing mitochondrial fragmentation and 8-OHdG enrichment, thus restoring high levels of co-localization. These results provide direct visual evidence that IQ attenuates IL-1β-induced mitochondrial oxidative DNA damage in an AKT1-dependent manner.

Location of the changes: Row 173-185; 511-518; Fig. S12.

• Since your core claim involves mitochondrial dynamics, I suggest (if possible) to add a functional assay like membrane potential or ATP production via cytometry or fluorescence microscopy

Response: We appreciate this constructive suggestion. While we did not perform additional cytometry-based assays in the current study, we have already functionally validated mitochondrial integrity using JC-1 staining (Figs 3I-J), which directly reflects mitochondrial membrane potential—a core readout of mitochondrial function. Our Western blot results further demonstrated that IQ significantly restored the expression of fusion proteins (OPA1, MFN1/2) and suppressed fission-related proteins (FIS1, p-DRP1) (Figs 3A-H), consistent with improved mitochondrial homeostasis. Accumulating evidence confirms that restoring fusion-fission balance directly rescues mitochondrial function in chondrocytes (refs., Kidney International. 2021, 99, 870–84; Phytomedicine. 2025, 141, 156672). AKT1

Attachment Submitted filename: Response to reviewers.docx

# Peer review: Decision Letter 1

**DOI:** 10.1371/journal.pone.0357989.r003

**Authors:** David Chau

23 Jul 2026

Dear Dr. Zhang,

Thank you for submitting your manuscript to PLOS ONE. After careful consideration, we feel that it has merit but does not fully meet PLOS ONE’s publication criteria as it currently stands. Therefore, we invite you to submit a revised version of the manuscript that addresses the points raised during the review process.

Please submit your revised manuscript by Sep 06 2026 11:59PM. If you will need more time than this to complete your revisions, please reply to this message or contact the journal office at plosone@plos.org. When you're ready to submit your revision, log on to https://www.editorialmanager.com/pone/ and select the 'Submissions Needing Revision' folder to locate your manuscript file.

- A letter that responds to each point raised by the academic editor and reviewer(s). You should upload this letter as a separate file labeled 'Response to Reviewers'.
- A marked-up copy of your manuscript that highlights changes made to the original version. You should upload this as a separate file labeled 'Revised Manuscript with Track Changes'.
- An unmarked version of your revised paper without tracked changes. You should upload this as a separate file labeled 'Manuscript'.

If you would like to make changes to your financial disclosure, please include your updated statement in your cover letter. Guidelines for resubmitting your figure files are available below the reviewer comments at the end of this letter.

If applicable, we recommend that you deposit your laboratory protocols in protocols.io to enhance the reproducibility of your results. Protocols.io assigns your protocol its own identifier (DOI) so that it can be cited independently in the future. For instructions see: https://journals.plos.org/plosone/s/submission-guidelines#loc-laboratory-protocols. Additionally, PLOS ONE offers an option for publishing peer-reviewed Lab Protocol articles, which describe protocols hosted on protocols.io. Read more information on sharing protocols at https://plos.org/protocols?utm_medium=editorial-email&utm_source=authorletters&utm_campaign=protocols.

As the corresponding author, your ORCID iD is verified in the submission system and will appear in the published article. PLOS supports the use of ORCID, and we encourage all coauthors to register for an ORCID iD and use it as well. Please encourage your coauthors to verify their ORCID iD within the submission system before final acceptance, as unverified ORCID iDs will not appear in the published article. *Only*  the individual author can complete the verification step; PLOS staff *cannot*  verify ORCID iDs on behalf of authors.

We look forward to receiving your revised manuscript.

Kind regards,

David Chau

Academic Editor

PLOS One

Journal Requirements:

If the reviewer comments include a recommendation to cite specific previously published works, please review and evaluate these publications to determine whether they are relevant and should be cited. There is no requirement to cite these works unless the editor has indicated otherwise.

Please review your reference list to ensure that it is complete and correct. If you have cited papers that have been retracted, please include the rationale for doing so in the manuscript text, or remove these references and replace them with relevant current references. Any changes to the reference list should be mentioned in the rebuttal letter that accompanies your revised manuscript. If you need to cite a retracted article, indicate the article’s retracted status in the References list and also include a citation and full reference for the retraction notice.

[Note: HTML markup is below. Please do not edit.]

Reviewers' comments:

Reviewer's Responses to Questions

**Comments to the Author**

Reviewer #1: (No Response)

Reviewer #2: All comments have been addressed

Reviewer #3: All comments have been addressed

**********

2. Is the manuscript technically sound, and do the data support the conclusions?

Reviewer #1: Partly

Reviewer #2: Yes

Reviewer #3: Yes

**********

3. Has the statistical analysis been performed appropriately and rigorously?

Reviewer #1: No

Reviewer #2: Yes

Reviewer #3: Yes

**********

4. Have the authors made all data underlying the findings in their manuscript fully available?

The PLOS Data policy

Reviewer #1: Yes

Reviewer #2: Yes

Reviewer #3: Yes

**********

5. Is the manuscript presented in an intelligible fashion and written in standard English?

Reviewer #1: Yes

Reviewer #2: Yes

Reviewer #3: Yes

**********

Reviewer #1: 1. The dose of IQ (40 µM) is therefore still unvalidated in the presence of IL 1β (Fig 1);

is clearly evident that 40 μM is a tolerated concentration, but No dose–response for IQ under IL-1β was ever performed.

I think this is important

2. “Results showed that IQ concentrations ≤ 80 μM had no adverse impact on chondrocyte viability, whereas 160 μM IQ induced slight cytotoxicity (Fig 1B).”

an IC₅₀ of 95.05 µM requires that viability at 160 µM be well below 50%; So "slight cytotoxicity" and "≤80 µM had no adverse impact" cannot both be true alongside the reported IC₅₀

3. The wavelength (490 nm) appears only in the rebuttal, not in the manuscript (Rows 109–112 give the instrument only).

4. “Explain the ImageJ protocol for blot quantification”, you did not added in the manuscript a clear protocol The grayscale / background-subtraction / integrated-density description exists only in the rebuttal

5. Statistical analysis: defines significance only as *P<0.05 and **P<0.01, while every figure legend uses *** and ****.

6. I was wondering, why there is not IQ-alone group (IQ without IL‑1β) in any functional assay, so IQ's baseline effect on ECM, cytokines and mitochondrial proteins is unknown. an IQ-only arm would also strengthen the safety claim you make in the opening Results paragraph.

7. The opening Results paragraph on ADMET/ecotoxicity ("low toxicity to aquatic and terrestrial organisms, no bioaccumulation") is uncited, is not a result of this study

8. “Other common chemical reagents including PFA and SKIM MILK were purchased from domestic qualified suppliers.” Still in the manuscript, but in the rebuttal <<our any="" blocking="" experiment.="" for="" in="" membrane="" milk="" never="" research="" skim="" team="" used="" wb="">>

9. I suggest a a 2–3 point dose–response (e.g. 10 / 40 / 80 μM) for p-DRP1/DRP1 for IQ under IL-1β (a single western blot gel)

10. Fig 7 E, DRP1 in those with IQ is visible a doublet.

Please state which band was used for total DRP1 quantification, and whether both were included. If the relative intensity of the two bands changes across conditions, this should be commented on.

11. Pay attention for the claims that exist in rebuttal, but are absent from manuscript; double check all.</our>

Reviewer #2: (No Response)

Reviewer #3: No additional comments. Authors have replied to all my previuos comments.

**********

what does this mean?). If published, this will include your full peer review and any attached files.

If you choose “no”, your identity will remain anonymous but your review may still be made public.

**Do you want your identity to be public for this peer review?**  For information about this choice, including consent withdrawal, please see our Privacy Policy

Reviewer #1: No

Reviewer #2: No

Reviewer #3: No

**********

[NOTE: If reviewer comments were submitted as an attachment file, they will be attached to this email and accessible via the submission site. Please log into your account, locate the manuscript record, and check for the action link "View Attachments". If this link does not appear, there are no attachment files.]

To ensure your figures meet our technical requirements, please review our figure guidelines: https://journals.plos.org/plosone/s/figures

You may also use PLOS’s free figure tool, NAAS, to help you prepare publication quality figures: https://journals.plos.org/plosone/s/figures#loc-tools-for-figure-preparation.

NAAS will assess whether your figures meet our technical requirements by comparing each figure against our figure specifications.

# Author comment: Author response to Decision Letter 2

**DOI:** 10.1371/journal.pone.0357989.r004

16 Aug 2026

Reviewer #1:

1. The dose of IQ (40 µM) is therefore still unvalidated in the presence of IL 1β (Fig 1); is clearly evident that 40 μM is a tolerated concentration, but No dose–response for IQ under IL-1β was ever performed. I think this is important.

Response: To address this issue completely, we supplemented a new CCK‑8 viability experiment where C28/I2 chondrocytes were co‑treated with fixed 10 ng/mL IL‑1β and serial concentrations of IQ (0, 5, 10, 20, 40, 80 μM) for 24 h (Fig. S1C). Consistent with our functional phenotypes, IL‑1β treatment markedly suppressed chondrocyte viability compared with the normal control group. IQ improved cell viability in a concentration‑dependent manner from 5 μM to 40 μM, reaching the maximal protective effect at 40 μM. When the concentration rose to 80 μM, the protective efficacy of IQ declined, accompanied by obvious cytotoxicity under inflammatory conditions.

These supplementary data robustly validate that 40 μM is the optimal therapeutic concentration for subsequent functional and mechanistic assays under IL‑1β stimulation.

Revised sentence added to Results: “Second, we performed a targeted dose–response assay under standardized 10 ng/mL IL‑1β inflammatory stimulation (Figs S1C). IL‑1β alone drastically reduced chondrocyte viability. Co‑administration of IQ produced gradual, concentration‑dependent rescue of cell viability from 5 μM up to 40 μM, which yielded the maximum protective effect. However, the protective capacity declined sharply at 80 μM IQ, demonstrating obvious cytotoxicity in the inflammatory microenvironment. Collectively, the dual CCK‑8 datasets confirm that 40 μM IQ achieves maximal chondroprotection without observable toxicity under IL‑1β injury, justifying the use of this concentration for all subsequent functional experiments.”

Revised sentence added to Materials and Methods:“Second, dose–response detection under IL‑1β-induced inflammatory injury: chondrocytes were co-incubated with 10 ng/mL IL‑1β and serial IQ concentrations (0, 5, 10, 20, 40, 80 μM) for 24 h. Subsequently, 10 μL of CCK-8 reagent was supplemented to each well. After 2–4 h of incubation in the dark, optical density (OD) values were measured via a Multiskan FC microplate reader (Thermo Fisher Scientific, USA).”

Location of the changes: Fig. S1; Raw113-117; 289-297; 598-605.

2. “Results showed that IQ concentrations ≤ 80 μM had no adverse impact on chondrocyte viability, whereas 160 μM IQ induced slight cytotoxicity (Fig 1B).”

an IC₅₀ of 95.05 µM requires that viability at 160 µM be well below 50%; So "slight cytotoxicity" and "≤80 µM had no adverse impact" cannot both be true alongside the reported IC₅₀

Response: We apologize for the contradictory description inconsistent with the IC₅₀ value. After careful inspection, we identified inappropriate qualitative summaries in the original manuscript caused by coarse concentration gradients. The statement “IQ concentrations ≤ 80 μM had no adverse impact on chondrocyte viability, whereas 160 μM IQ induced slight cytotoxicity” conflicts with the IC₅₀ fitting result and has therefore been fully deleted. We optimized the IQ concentration layout and re-performed four-parameter logistic regression analysis based on updated viability data. The revised IC₅₀ is 91.76 μM (Figs. S1B, C). In the revised Results section, we only objectively describe the trend that cell viability decreases gradually as IQ concentration increases, avoiding inaccurate subjective judgments.

Revised sentence added to Results: “Prior to functional assays, two independent CCK‑8 viability tests were conducted to identify the optimal IQ dosage. First, broad-range gradient treatment without IL‑1β was used to evaluate intrinsic IQ cytotoxicity. Four‑parameter nonlinear regression fitted an IC₅₀ of 91.76 μM for resting chondrocytes; mild growth inhibition emerged above 80 μM, while concentrations ≤40 μM exerted negligible toxic effects on normal chondrocytes (Fig 1B and Figs S1A, B).”

Revised sentence added to Materials and Methods:“Two parallel CCK‑8 assays were performed separately: First, cytotoxic screening of IQ without IL‑1β stimulation: cells were exposed to IQ at concentrations of 0, 20, 40, 60, 80, 100, 120, 140, 160 μM for another 24 h to calculate the IC₅₀ value of IQ under normal culture conditions.”

Location of the changes: Fig. S1; Raw110-112; 285-289; 598-605.

3. The wavelength (490 nm) appears only in the rebuttal, not in the manuscript (Rows 109–112 give the instrument only).

Response: We appreciate the reviewer’s meticulous reading. As pointed out, the absorbance wavelength (490 nm) for the CCK‑8 measurement was only mentioned in our response letter and omitted within the main text. To fix this omission, we have added the wavelength parameter to the CCK‑8 experimental description in the Materials and methods section.

Revised sentence added to Materials and Methods: “After 2-4 h of incubation in the dark, optical density (OD) values were measured at 490 nm via a Multiskan FC microplate reader (Thermo Fisher Scientific, USA).”

Location of the changes: Raw108-109.

4. “Explain the ImageJ protocol for blot quantification”, you did not added in the manuscript a clear protocol The grayscale / background-subtraction / integrated-density description exists only in the rebuttal

Response: We appreciate this meticulous comment. The detailed ImageJ blot quantification workflow was only mentioned in our previous rebuttal and absent from the original manuscript. We have supplemented the full quantitative operation steps in the Western blotting subsection of Materials and Methods. The added content clarifies grayscale conversion, unified background subtraction and integrated density normalization against β‑actin internal control, and all revisions are highlighted in the tracked-changes manuscript for easy checking.

Revised sentence added to Materials and Methods: “Unmodified original blot images were quantified with ImageJ software (National Institutes of Health, Bethesda, MD, USA; https://imagej.net/ij/) [34]: images were converted to grayscale, background was subtracted uniformly, and relative protein expression was calculated as the integrated density of target band divided by that of β‑actin (internal control).”

Location of the changes: Raw155-159.

5. Statistical analysis: defines significance only as *P<0.05 and **P<0.01, while every figure legend uses *** and ****.

Response: We sincerely thank the reviewer for this rigorous inspection of our statistical criteria consistency. We have updated the Statistical analysis section to fully supplement the definitions of ***P < 0.001, ****P < 0.0001; #P < 0.05, ##P < 0.01, ###P < 0.001, ####P < 0.0001, matching all labeling standards in each figure. All revisions are marked in the tracked-changes manuscript.

Revised sentence added to Materials and Methods: “One-way analysis of variance (ANOVA) combined with Tukey’s post hoc test was applied for multiple group comparisons using GraphPad Prism 9.5.0 for Windows (GraphPad Software, Inc., San Diego, CA, USA; https://www.graphpad.com); significance was defined as *P < 0.05, **P < 0.01, ***P < 0.001, ****P < 0.0001 and #P < 0.05, ##P < 0.01, ###P < 0.001, ####P < 0.0001.”

Location of the changes: Raw 274-275.

6. I was wondering, why there is not IQ-alone group (IQ without IL‑1β) in any functional assay, so IQ's baseline effect on ECM, cytokines and mitochondrial proteins is unknown. an IQ-only arm would also strengthen the safety claim you make in the opening Results paragraph.

Response: We sincerely appreciate this valuable comment raised by the reviewer. We fully recognize the deficiency that no IQ-only treatment group (without IL‑1β stimulation) was included in our functional detection system, which makes the baseline biological effects of IQ on ECM anabolic proteins, pro-inflammatory cytokines and mitochondrial dynamic proteins unclear under physiological conditions; this omission also weakens the biosafety statement of IQ described at the beginning of the Results section, and we acknowledge this as an inadequate experimental design in our original manuscript.

For our initial experimental layout, we referenced mainstream chondrocyte OA studies from published flavonoid-related literature (refs., J. Nutr. Biochem. 2026, 153, 110306; Molecules 2022, 27, 3459; Aging 2023, 15:4861-4874). The core scientific focus of this study is to clarify the rescue effect and underlying mechanism of IQ against IL‑1β-evoked chondrocyte dysfunction. Therefore, almost all treatment groups were established on the basis of the IL‑1β-induced chondrocyte injury model, with the normal untreated group as the only blank control.

Unfortunately, due to limited remaining materials and tight experimental schedule, we are unable to supplement full sets of WB, qPCR and staining assays with an IQ-only group covering all functional indicators in this revision. To fix this flaw reasonably, we have made two key revisions to the manuscript:

1.We completely deleted the overemphasized descriptive sentences regarding the broad safety profile of IQ in the opening paragraph of Results, removing the unsupported biosafety claim that lacks corresponding IQ-only experimental evidence;

2.We supplemented a dedicated limitation subsection in the Discussion chapter, explicitly stating the absence of the IQ-only group as a present experimental shortcoming, and proposed relevant supplementary detection (ECM, inflammatory and mitochondrial markers under IQ single treatment) as a clear direction for our follow-up in-depth research.

Revised sentence added to Results: “One key limitation of the current work is the absence of an IQ single-treatment experimental group to independently dissect its standalone biological activity; subsequent follow-up work will supplement detections of ECM, inflammatory and mitochondrial markers under IQ monotherapy to fully clarify its intrinsic regulatory effects.”

Location of the changes: Raw 587-591.

7. The opening Results paragraph on ADMET/ecotoxicity ("low toxicity to aquatic and terrestrial organisms, no bioaccumulation") is uncited, is not a result of this study

Response: We fully agree with the reviewer’s valuable suggestion. The original descriptions about ADMET characteristics, aquatic and terrestrial ecotoxicity as well as bioaccumulation are irrelevant background information not generated from our present chondrocyte experiments, and the lack of citations also causes non-compliance with manuscript referencing norms. Therefore, we have completely deleted this paragraph of extraneous content.

8. “Other common chemical reagents including PFA and SKIM MILK were purchased from domestic qualified suppliers.” Still in the manuscript, but in the rebuttal <>

Response: We appreciate the reviewer’s careful reminder. We have fully removed this redundant sentence regarding common chemical reagents from the revised manuscript.

9. I suggest a 2–3 point dose–response (e.g. 10 / 40 / 80 μM) for p-DRP1/DRP1 for IQ under IL-1β (a single western blot gel)

Response: We appreciate the reviewer’s recommendation to test a 3-point dose response of IQ on p-DRP1/DRP1 under IL‑1β. Given the nearly identical molecular weight of p-DRP1 and DRP1, simultaneous detection on the same membrane is not feasible. We thus used sequential stripping and re-probing on one single gel containing all experimental groups.

The newly acquired western blot results consistently verify that IL‑1β significantly increases the p-DRP1 level and elevates the p-DRP1/DRP1 ratio (Fig. S6). IQ treatment reverses DRP1 phosphorylation in a dose-dependent manner: 40 μM IQ exhibits the strongest inhibitory effect, while the protective effect is partially attenuated at 80 μM IQ. These immunoblot data are highly consistent with our CCK-8 cell viability results, jointly confirming that 40 μM IQ represents the optimal working concentration to suppress excessive DRP1 phosphorylation and mitochondrial fission.

Revised sentence added to Results: “To further clarify the optimal working concentration of IQ in regulating mitochondrial fission, we established a 3-point dose-response model under IL‑1β stimulation (Fig. S6). Treatment with IQ dose-dependently suppressed IL‑1β-triggered DRP1 activation. The maximal inhibitory effect was observed at 40 μM IQ, whereas the protective effect was partially weakened at 80 μM IQ. Consistent with our CCK‑8 cell viability data, these results confirm that 40 μM IQ is the optimal concentration to restrain excessive DRP1 phosphorylation.”

Location of the changes: FigS6; S17; Raw 366-372; 626-632; 668.

10. Fig 7 E, DRP1 in those with IQ is visible a doublet.

Please state which band was used for total DRP1 quantification, and whether both were included. If the relative intensity of the two bands changes across conditions, this should be commented on.

Response: We thank the reviewer for this critical observation. For total DRP1 quantification in Fig.7E, we summed the integrated density of both the upper and lower bands of the DRP1 doublet using a unified ROI in ImageJ. This inter-group band variation has been fully counted in our total DRP signal without introducing analytical deviation. A corresponding description of our doublet quantification method has been added to the Figure 7 legend, with all revisions highlighted in the tracked manuscript.

Revised sentence added to the Figure 7 legend: “Note: Total DRP1 appeared as a doublet band on immunoblots. Both upper and lower bands were enclosed in one ROI and their total integrated density was summed for quantification.”

Location of the changes: Raw 533-535.

11. Pay attention for the claims that exist in rebuttal, but are absent from manuscript; double check all.

Response: We thank the reviewer for this important reminder. We have thoroughly cross-referenced all contents in the prior rebuttal against the full manuscript. All key experimental parameters, quantification protocols, statistical rules and supplementary experimental schemes that were only written in the rebuttal have been supplemented into the revised main text. Irrelevant redundant paragraphs have been completely removed.

Attachment Submitted filename: Response_to_reviewers_auresp_2.docx

# Peer review: Decision Letter 2

**DOI:** 10.1371/journal.pone.0357989.r005

**Authors:** David Chau

25 Aug 2026

Isoquercitrin Ameliorates Chondrocyte Dysfunction in Osteoarthritis by Targeting AKT1-Mediated Mitochondrial Dynamics Regulation

PONE-D-26-14316R2

Dear Dr. Zhang,

We’re pleased to inform you that your manuscript has been judged scientifically suitable for publication and will be formally accepted for publication once it meets all outstanding technical requirements.

Within one week, you’ll receive an e-mail detailing the required amendments. When these have been addressed, you’ll receive a formal acceptance letter and your manuscript will be scheduled for publication.

An invoice will be generated when your article is formally accepted. Please note, if your institution has a publishing partnership with PLOS and your article meets the relevant criteria, all or part of your publication costs will be covered. Please make sure your user information is up-to-date by logging into Editorial Manager at Editorial Manager® and clicking the ‘Update My Information' link at the top of the page. For questions related to billing, please contact billing support.

If your institution or institutions have a press office, please notify them about your upcoming paper to help maximize its impact. If they’ll be preparing press materials, please inform our press team as soon as possible -- no later than 48 hours after receiving the formal acceptance. Your manuscript will remain under strict press embargo until 2 pm Eastern Time on the date of publication. For more information, please contact onepress@plos.org.

Kind regards,

David Chau

Academic Editor

PLOS One

Additional Editor Comments (optional):

Reviewers' comments:

Reviewer's Responses to Questions

**Comments to the Author**

Reviewer #1: All comments have been addressed

**********

2. Is the manuscript technically sound, and do the data support the conclusions?

Reviewer #1: Yes

**********

3. Has the statistical analysis been performed appropriately and rigorously?

Reviewer #1: Yes

**********

4. Have the authors made all data underlying the findings in their manuscript fully available?

The PLOS Data policy

Reviewer #1: Yes

**********

5. Is the manuscript presented in an intelligible fashion and written in standard English?

Reviewer #1: Yes

**********

Reviewer #1: (No Response)

**********

what does this mean?). If published, this will include your full peer review and any attached files.

If you choose “no”, your identity will remain anonymous but your review may still be made public.

**Do you want your identity to be public for this peer review?**  For information about this choice, including consent withdrawal, please see our Privacy Policy

Reviewer #1: No

**********

# Editor report: Acceptance letter

**DOI:** 10.1371/journal.pone.0357989.r006

**Authors:** David Chau

PONE-D-26-14316R2

PLOS One

Dear Dr. Zhang,

I'm pleased to inform you that your manuscript has been deemed suitable for publication in PLOS One. Congratulations! Your manuscript is now being handed over to our production team.

At this stage, our production department will prepare your paper for publication. This includes ensuring the following:

* All references, tables, and figures are properly cited

* All relevant supporting information is included in the manuscript submission,

* There are no issues that prevent the paper from being properly typeset

You will receive further instructions from the production team, including instructions on how to review your proof when it is ready. Please keep in mind that we are working through a large volume of accepted articles, so please give us a few days to review your paper and let you know the next and final steps.

Lastly, if your institution or institutions have a press office, please let them know about your upcoming paper now to help maximize its impact. If they'll be preparing press materials, please inform our press team within the next 48 hours. Your manuscript will remain under strict press embargo until 2 pm Eastern Time on the date of publication. For more information, please contact onepress@plos.org.

You will receive an invoice from PLOS for your publication fee after your manuscript has reached the completed accept phase. If you receive an email requesting payment before acceptance or for any other service, this may be a phishing scheme. Learn how to identify phishing emails and protect your accounts at https://explore.plos.org/phishing.

If we can help with anything else, please email us at customercare@plos.org.

Thank you for submitting your work to PLOS One and supporting open access.

Kind regards,

PLOS One Editorial Office Staff

on behalf of

Dr. David Chau

Academic Editor

PLOS One
